# Systematic review and meta-analysis of cardiac neurosis for development of clinical practice guidelines of Korean medicine

**DOI:** 10.3389/fpsyt.2024.1302245

**Published:** 2024-02-12

**Authors:** Hui-Yeong Park, Hyun Woo Lee, Geum-Ju Song, Sunggyu Hong, Sunghee Hong, Hyo-Weon Suh, Seok-In Yoon, Chan Park, Sun-Yong Chung, Jong Woo Kim

**Affiliations:** ^1^ Department of Neuropsychiatry, College of Korean Medicine, Kyung Hee University, Seoul, Republic of Korea; ^2^ Department of Neuropsychiatry, Kyung Hee University Korean Medicine Hospital at Gangdong, Seoul, Republic of Korea; ^3^ Industry-Academic Cooperation Foundation, Kyung Hee University, Seoul, Republic of Korea; ^4^ Health Policy Research Team, Division of Healthcare Research, National Evidence-based Healthcare Collaborating Agency, Gwangjin-gu, Seoul, Republic of Korea

**Keywords:** cardiac neurosis, autonomic dysfunction, Korean medicine, systematic review, meta-analysis

## Abstract

**Background:**

The development of clinical practice guidelines in traditional medicine requires evidence that sufficiently reflects the medical field. Cardiac neurosis is a disease that occurs because of problems in the autonomic nervous system and is characterized by symptoms of the circulatory system that are representative of autonomic dysfunction. In traditional medicine, the heart is considered to be involved in mental health problems, and cardiac neurosis is accompanied by a variety of mental symptoms. Furthermore, there is a categorized diagnosis for cardiac neurosis, and active empirical research is being conducted in China.

**Objective:**

This study aimed to systematically review and quantitatively synthesize the effects of Korean medicine treatments in patients with cardiac neurosis to develop evidence-based clinical practice guidelines for the treatment of autonomic dysfunction.

**Methods:**

Nine databases were searched for articles published before September 13, 2022. The methodological quality of the studies was assessed using the RoB tool. The primary outcomes were somatization, depression, anxiety, and effectiveness rate. The secondary outcome was the rate of adverse effects.

**Results:**

Based on a systematic literature review, 151 randomized controlled trials were selected and analyzed. For patients with cardiac neurosis, herbal medicine, combined treatment of herbal medicine and Western medicine, combined treatment of herbal medicine and acupuncture, acupuncture, and combined treatment of acupuncture and Western medicine showed better overall effects than Western medicine alone. Furthermore, the combined treatment of herbal medicine and psychotherapy and that of herbal medicine, psychotherapy, and Western medicine showed an overall better effect than the combined treatment of Western medicine and psychotherapy.

**Conclusion:**

A meta-analysis of articles revealed the effectiveness of Korean medicine treatments and verified the effectiveness of a Korean medicine treatment alone, Korean medicine combined treatment, and combined treatment of Korean medicine and Western medicine on cardiac neurosis. Limitations included the inability to verify the cause of high heterogeneity between studies and the poor quality of the included studies. Nevertheless, this systematic review and meta-analysis of cardiac neurosis showed that the disease concept of traditional medicine can also be organized based on the latest research. Future research related to traditional diseases such as these should be conducted.

**Systematic review registration:**

https://www.crd.york.ac.uk/prospero/display_record.php?ID=CRD42022347992, identifier CRD42022347992.

## 1 Introduction

Autonomic dysfunction refers to a symptom of poor control of the autonomic nervous system. In general, chronic reactions to stress cause abnormalities in the autonomic nervous system. As this condition progresses, an immune response occurs, and the stage before it is categorized as a specific disease that can be referred to as a state of autonomic dysfunction. Because the autonomic nervous system affects the entire body, autonomic dysfunction also causes various symptoms in various body organs. When strong symptoms appear in a specific area, a different name for the disease may be used (1, 2).

Autonomic dysfunction is not commonly used in Western categorical diagnostic systems but is mainly used in East Asian traditional medicine. Autonomic dysfunction is used as a disease containing a traditional disease concept in East Asia. In Japan, autonomic dysfunction is treated as a subclass of psychosomatic disease; in China, it is explained in connection with cardiac neurosis; and in Korea, it is explained as a disharmony between yin and yang. Autonomic dysfunction, which refers to an imbalance between the sympathetic and parasympathetic nervous systems, reflects traditional medicine’s integrated approach to the mind and body and the position that disorders and diseases occur because of abnormalities in harmony and balance.

The range of disorders caused by abnormalities in the autonomic nervous system is wide, and they are closely related to almost all diseases to the extent that it is difficult to identify a disease that is not medically applicable. Because of the nature of autonomic dysfunction, which causes a variety of symptoms, it is sometimes understood as a collection of medically unexplained symptoms and used as a convenient diagnostic name to convince patients to name symptoms for which no abnormality is found during examination (1, 2).

Currently, in Korea, heart rate variability (HRV) is used as an oriental medical device, and its use for autonomic dysfunction is increasing. However, little research has been conducted to categorize autonomic dysfunction as a specific diagnosis, and it is difficult to establish consistent standards for the definition of autonomic dysfunction, diagnostic criteria, and treatment. Accordingly, the development of clinical practice guidelines that include a standardized approach to autonomic dysfunction is necessary.

This study attempted to derive this concept by organizing the literature on autonomic dysfunction in order to develop clinical practice guidelines for Korean medicine. Three factors were considered during this process. The first is the cause of autonomic nervous system imbalances. In clinical practice, overactivity of the sympathetic nervous system is often problematic. Autonomic dysfunction can be understood as overactivity of the sympathetic nervous system due to a stress response. The second is the representative physical symptom of autonomic dysfunction. Among the various body parts governed by the autonomic nervous system, the circulatory system is the most significantly affected. Therefore, the basic problems related to the circulatory system in autonomic dysfunction can be addressed. The third factor is the variety of mental symptoms caused by autonomic dysfunction. Anxiety is the most common symptom, and a variety of other emotional and mental symptoms, such as depression, may appear. Accordingly, in this study, autonomic dysfunction was defined as ‘autonomic dysfunction caused by stress without organic disorder’.

In the development of clinical practice guidelines for traditional medicine, it is important to make efforts to secure evidence that sufficiently reflects the medical field. To collect empirical evidence on autonomic dysfunction, this study sought to select target diseases that reflected the concept of autonomic dysfunction described above and had a categorized diagnosis. Although various candidate diseases existed, cardiac neurosis was selected as the main target disease for autonomic dysfunction.

Cardiac neurosis is a term first used by White in 1951, and was mainly studied in the West in the early and mid-20th century. However, little recent research has been conducted on cardiac neurosis in the West, and accurate definitions and diagnostic criteria are difficult to find. Meanwhile, in China, cardiac neurosis is being actively treated and is viewed as a disease of the autonomic nervous system with a focus on circulatory system symptoms.

According to Chinese literature, cardiac neurosis is a psychogenic disease and functional neurosis that is mainly characterized by heart discomfort. Patients usually show complex and diverse subjective symptoms, along with anxiety, depression, fear, obsessive-compulsive disorder, hypochondria, and other psychological disorders (3).

Cardiac neurosis is a disease name mainly used in China and accounts for 10% of the cardiovascular diseases in the country. It occurs in young people and adults, often in people in their 20s and 40s (4). It is especially common in postmenopausal women. Recently, the incidence of the disease has increased as the pace of life accelerates and social pressure increases (5).

The primary prerequisite for cardiac neurosis is the exclusion of organic heart diseases. According to the literature, the diagnosis of cardiac neurosis can be divided into two types: Western medicine and traditional Chinese medicine (6). Western medical diagnostic criteria include cardiovascular symptoms, such as palpitations and anterior chest pain, and autonomic dysfunction symptoms, such as dyspnea and sweating (7, 8).

The traditional Chinese medicine standards are similar to those mentioned above. The main symptoms include subjective palpitations, anxiety, chest discomfort, and pain. Dyspnea, other symptoms of the autonomic nervous system, and psychiatric symptoms are secondary symptoms. When the pulse on the wrist was examined, the pattern was as follows: thin and fast, thin and gritty, hollow and irregular (9).

Thus, cardiac neurosis reflects circulatory system symptoms that are representative of autonomic dysfunction, and various other systemic autonomic dysfunction symptoms. Considering that the heart is traditionally regarded as an organ that controls mental health (10), cardiac neurosis reflects the traditional medical concept of autonomic dysfunction. In addition, because cardiac neurosis is a disease for which systematic diagnostic criteria exist (6), it may be easy to collect empirical evidence. Therefore, to develop clinical practice guidelines of Korean medicine for autonomic dysfunction, this study systematically reviewed and quantitatively summarized the effects of various Korean medicine treatments for cardiac neurosis that reflect the concept of autonomic dysfunction and have a categorized diagnosis.

## 2 Methods

### 2.1 Study design and registration

We conducted a systematic review and meta-analysis in accordance with the Preferred Reporting Items for Systematic Reviews and Meta-Analyses (PRISMA) guidelines (11). The predefined protocol was registered in PROSPERO (registration number: CRD42022347992).

### 2.2 Eligibility criteria

We defined the following inclusion criteria: (1) published papers and dissertations that performed randomized controlled trials (RCTs); (2) studies on adult cardiac neurosis patients; (3) studies on Korean medicine interventions (herbs, acupuncture, manual therapy, psychotherapy, and self-management methods) as a test group; and (4) studies measuring somatization symptoms, depression, anxiety, effectiveness rate, or adverse effects. RCTs that complied with the following criteria were excluded: (a) concomitant severe mental disorder (e.g., schizophrenia, delusional disorder, intellectual disability), (b) Korean medicine intervention as the sole control group, (c) auricular acupuncture as a stand-alone intervention, and (d) insufficient data.

### 2.3 Literature search

A systematic search was conducted using three English databases (MEDLINE/PubMed, CENTRAL, and EMBASE), one Chinese database (CNKI), one Japanese database (CiNii), and four Korean databases [Korean Medical Database (KMbase), Korean Studies Information Service System (KISS), Oriental Medicine Advanced Searching Integrated System (OASIS), and ScienceON] on September 13, 2022. Medical subject heading (MeSH) terms and free words were used in the search (**Supplementary Table 1**).

### 2.4 Primary and secondary outcomes

The primary outcomes were somatization, anxiety, depression, and effectiveness rates. The effectiveness rate is an indicator frequently used in the Chinese medical literature. The therapist evaluated the therapeutic effect of the intervention on four levels: basically cured, markedly improved, improved, and unimproved. The effectiveness rate was calculated as the percentage of patients who were basically cured, markedly improved, or improved out of the total number of patients included.

The secondary outcome was the incidence of adverse effects. The adverse effect rate was calculated as the proportion of patients experiencing adverse effects among all patients.

### 2.5 Data extraction

Two reviewers (HWS and SHH) independently screened the titles and abstracts of all the identified articles. The studies selected for the first screening were divided in half, and four authors (SGH, SHH, HYP, and HWL) paired two each independently reviewed the full text and conducted a second screening. Four authors extracted data from eligible full-text articles. Discrepancies were resolved through discussion with a third reviewer (HWS). The data collected included the first author, year of publication, country, effect size calculation indicators (e.g., mean and standard deviation, sample size of experimental and control groups), primary and secondary outcomes, quality of research, age, sex, inclusion criteria, intervention, control group, and duration of the intervention (weeks).

### 2.6 Assessment of study quality

According to the RoB 1.0 assessment tool in the Cochrane Handbook, the included studies were divided into two sets, and the quality of the studies was assessed by four authors (SGH, SHH, HYP, and HWL), with two independent raters in each set. The risk of bias included the following aspects: (1) random sequence generation, (2) allocation concealment, (3) blinding of participants and personnel, (4) blinding of outcome assessment, (5) incomplete outcome data, (6) selective reporting, and (7) other biases. Based on the Cochrane Assessment Tool. The judgments for these domains were categorized as “low risk of bias,” “high risk of bias,” or “unclear risk of bias.” Disagreements between the two raters were resolved through discussion, and when a consensus could not be reached, a decision was made by another author (HWS).

### 2.7 Data synthesis and analysis

RevMan software (version 5.4) was used for statistical analysis. Somatization, depression, and anxiety were continuous variables, whereas effectiveness rates and adverse effects were dichotomous variables. When the same scale was used among continuous variables, the mean difference (MD) was used, and when different scales were mixed, the standard mean difference (SMD) was used. The risk ratio (RR) was used as a dichotomous variable.

We hypothesized heterogeneity between the studies, considering that the included studies had different characteristics. Therefore, the summary effect size was estimated using a random-effects model rather than a fixed-effects model. However, if the number of studies was less than 10, a fixed-effects model was used.

First, the categories were divided according to the type of experimental and control groups, and analysis was performed for each variable. If there was only one study in this category, the analysis was not performed.

Next, the *I* ^2^ test was performed to evaluate the degree of heterogeneity among the studies. The *I* ^2^ value of 0% to less than 20% is considered homogeneous; 20% to less than 50%, to have low heterogeneity; 50% to less than 75%, to have moderate heterogeneity; and 75% or more, highly heterogeneous (12).

Finally, potential publication bias was examined by visual inspection of funnel plots. If there were fewer than 10 studies, the funnel plot was not evaluated. A *p* < 0.05 was considered statistically significant in all outcomes.

## 3 Results

### 3.1 Study selection and characteristics

**Figure 1:**
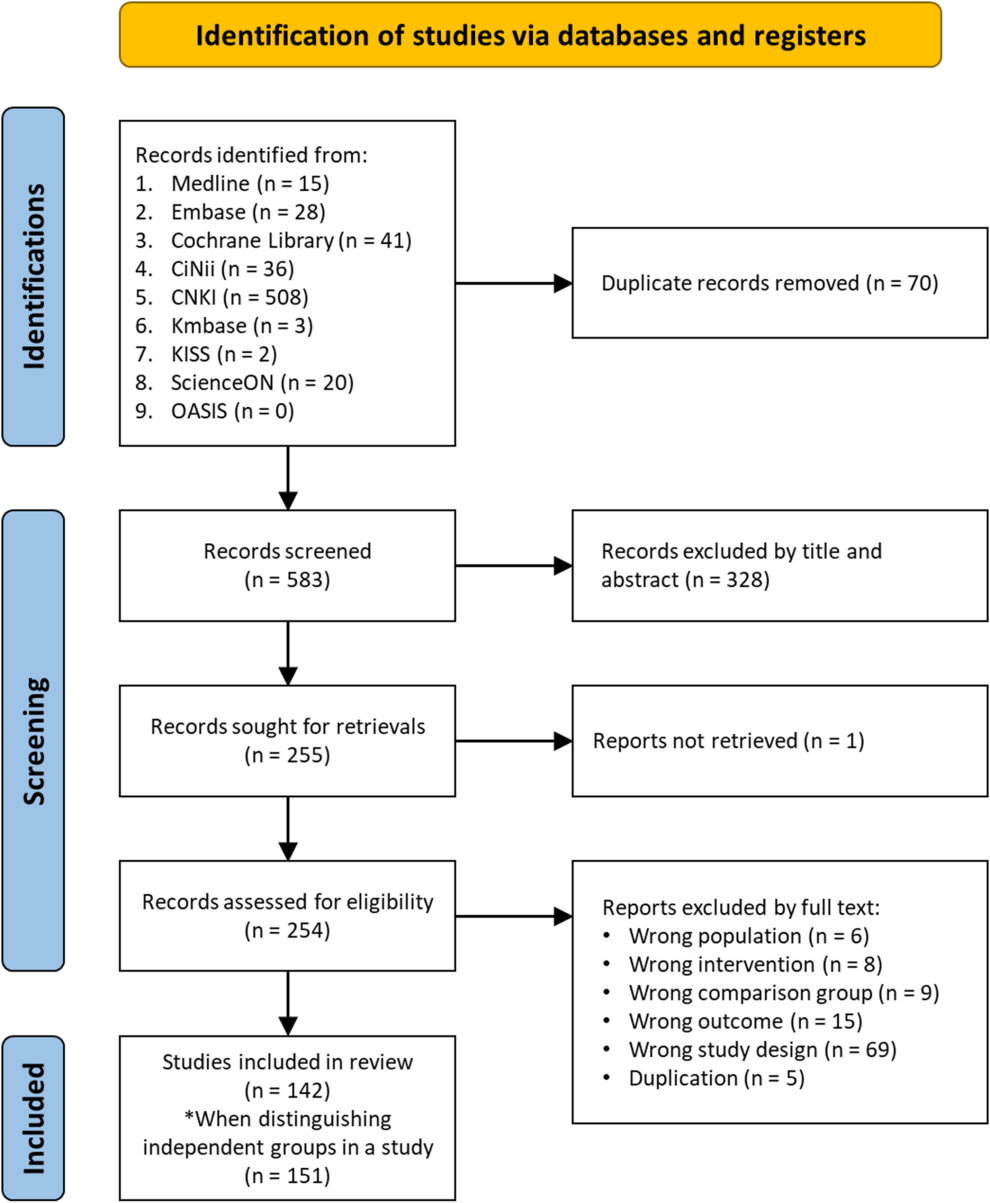
PRISMA flow chart.

Our electronic search yielded 653 articles. A total of 583 potential articles remained after duplicates were removed. We excluded 328 that failed to meet the inclusion criteria after title and abstract screening. After the full-text review, 112 articles were excluded and 142 articles were finally included (**Figure 1**). In addition, in the case of a three-armed design and two experimental groups meeting the intervention conditions of this study, the study was considered two independent studies sharing one control group. Based on these studies, 151 were analyzed.

The included studies were published from 1994 to 2022. All the studies were conducted in China. A total of 128 studies reported a mean age, with the mean being 41.01 years (SD = 6.38). There were 5 studies that reported median age, and the average median age was 40.56 years (SD = 7.75). Fourteen studies reported an age range of 18–69 years. Four studies did not report age. There were 13 cases in which there were more men in the group, 137 cases in which there were more or equal proportions of women, and 1 case in which it was unknown. The average proportion of women was 66.23% (SD = 13.44). The sample size ranged from 30 to 290, with an average of 88.38 (SD = 43.22).

Many studies used one standard diagnostic criterion (n = 51), while a corresponding number used two or more standard diagnostic criteria (n = 48). There were 18 cases in which detailed diagnostic criteria were revealed and used without reporting references and 34 cases in which diagnostic criteria were not reported. The most frequently used diagnostic criteria were Practical Internal Medicine (实用内科学) (n = 72), followed by Diagnostic and Therapeutic Criteria of TCM Syndromes (中医病证诊断疗效标准) (n = 21), Guiding Principles for Clinical Research of New TCM (中药新药临床研究指导原则) (n = 13), Chinese Internal Medicine (中医内科学) (n = 12), and Chinese Classification of Mental Disorders (CCMD. 中国精神障碍分类及诊断标准) (n = 11).

In terms of treatment methods, there were 67 cases of herbal medicine alone; 48 cases of combination treatment of herbal medicine and Western medicine; 2 cases of combination treatment of herbal medicine and acupuncture; 1 case of combination treatment of herbal medicine, acupuncture, and Western medicine; 8 cases of combination treatment of herbal medicine and psychotherapy; 9 cases of combination treatment of herbal medicine, psychotherapy, and Western medicine; 9 cases of acupuncture alone; 5 cases of combination treatment of acupuncture and Western medicine; 1 case of combination treatment of acupuncture and chuna; and 1 case of combination treatment of acupuncture and cupping. In most cases, Western medicine treatment was used as the control group; however, in the combination treatment of herbal medicine and psychotherapy and the combination treatment of herbal medicine, psychotherapy, and Western medicine, the combination treatment of Western medicine and psychotherapy was used as the control group. For the treatment of cardiac neurosis, the Korean medicine prescriptions used mainly herbal medicines for soothing the liver and relieving depression, such as Soyosan and Shihosogansan, and various types of calm, mind-type prescriptions. The Western medicine prescriptions used mainly autonomic dysfunction treatments such as r-oryzanol, beta-blockers, and anti-anxiety and antidepressant drugs. The treatment duration varied from 1 to 3 months (mean = 36.29, SD = 16.53).

Specific details of each study are described in the **Supplementary Material** (**Supplementary Table 2**).

### 3.2 Risk of bias in individual studies

The risk of bias in the included studies was evaluated according to the type of experimental and control groups. The results of the 67 studies comparing herbal medicine and Western medicine are shown in **Figure 2**, and other results are presented in the **Supplementary Material** (**Supplementary Figures 1-19**).

**Figure 2:**
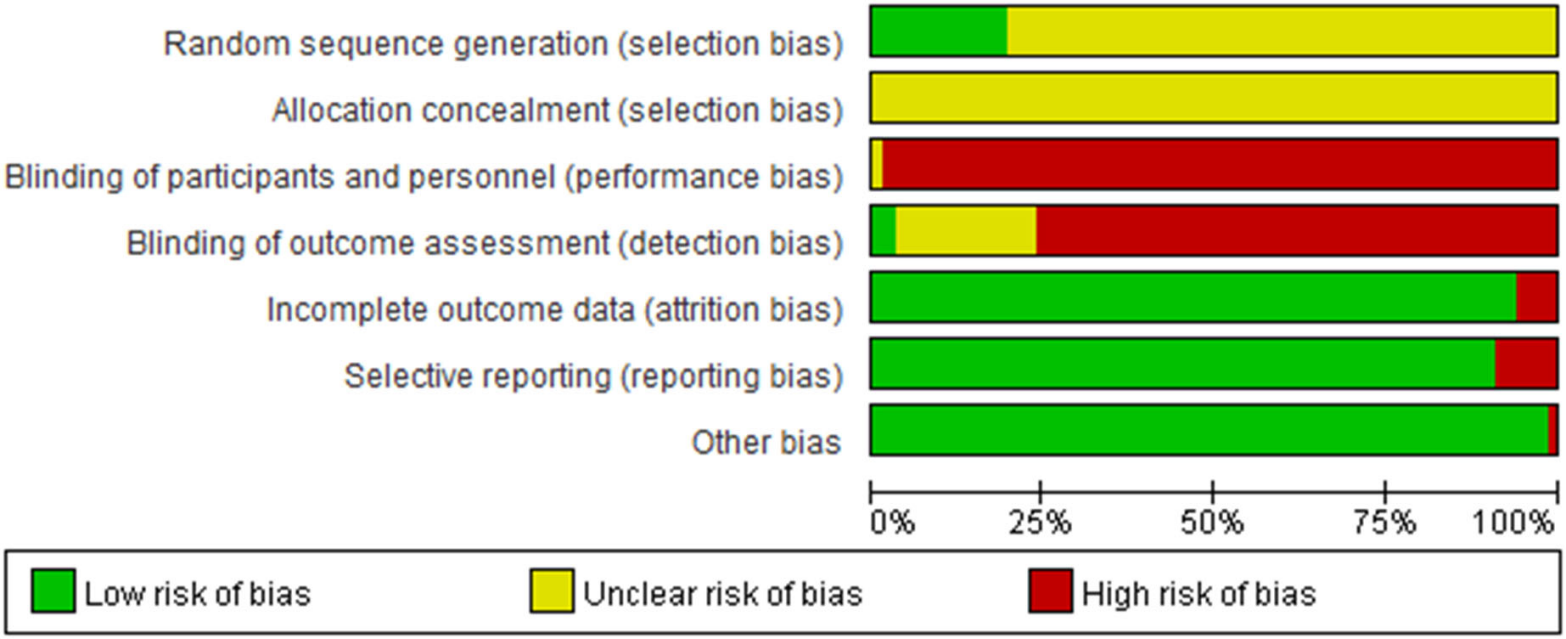
The risk of bias for 67 studies comparing herbal medicine and Western medicine.

#### 3.2.1 Random sequence generation

Among the 151 studies, 22 were evaluated as having a low risk of bias because the randomization method was well-performed, and the rest were evaluated as unclear, as there was no mention of this. No high risk of bias was observed.

#### 3.2.2 Allocation concealment

Only two cases had appropriate descriptions of allocation concealment; therefore, they were evaluated as having a low risk of bias. The rest were evaluated as unclear because they did not mention this. No high risk of bias was observed.

#### 3.2.3 Blinding of participants and personnel

Only one case was evaluated as unclear because there was no detailed information regarding blinding. Since the remaining studies were comparisons of herbal medicine, acupuncture, and psychotherapy with Western medicine, blinding of participants was deemed impossible and evaluated as having a high risk of bias.

#### 3.2.4 Blinding of outcome assessment

Four cases were evaluated as having a low risk of bias because there was an independent evaluator and blinding was judged to have been maintained. Thirty-eight studies were evaluated as unclear owing to a lack of detailed information, and the remaining studies were evaluated as having a high risk of bias because they used self-report scales without blinding the participants.

#### 3.2.5 Incomplete outcome data

Seven cases were evaluated as having a high risk of bias because appropriate statistical processing was not performed despite the difference in the number of dropouts between the groups. The remaining patients were evaluated as having a low risk of bias because there was no difference in the number of dropouts.

#### 3.2.6 Selective reporting

As the protocols of most studies could not be found, the items were evaluated according to whether the scale described in the Methods and the scale reported in the Results matched. Of the 151 studies, 9 had a high risk of bias, and the rest had a low risk of bias.

#### 3.2.7 Other potential sources

One study was evaluated as having a high risk of bias because the prior homogeneity was disrupted. All other studies had a low risk of bias.

### 3.3 Effects of Korean medicine interventions

#### 3.3.1 Herbal medicine vs. Western medicine

**Figure 3:**
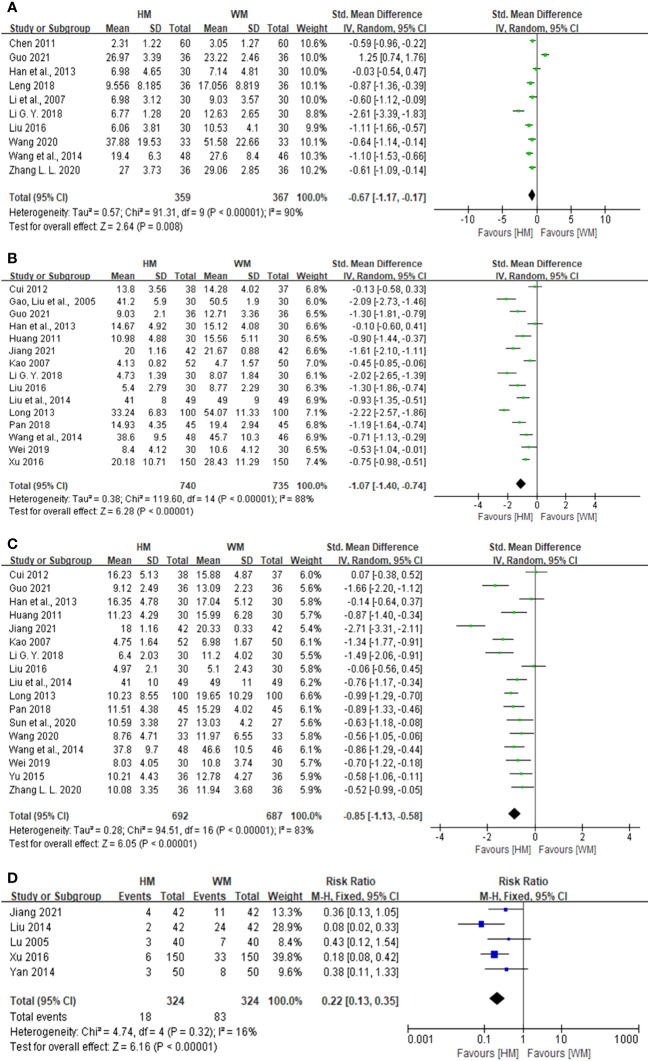
Forest plot showing the effect of herbal medicine versus Western medicine. **(A)** Forest plot for somatization, **(B)** forest plot for depression, **(C)** forest plot for anxiety, and **(D)** forest plot for adverse effect rates.

To compare the effects of herbal medicine with those of Western medicine on somatization in patients with cardiac neurosis, 10 studies involving 726 patients were analyzed. The results showed a significant difference in the reduction of somatization scores between herbal medicine and Western medicine [SMD: -0.67, 95% confidence interval (CI): -1.17 to -0.17, *p* = 0.008; **Figure 3A**]. There was a high heterogeneity in the results (*I* ^2^ = 90%).

To compare the effects of herbal medicine with those of Western medicine on depression in patients with cardiac neurosis, 15 studies involving 1,475 patients were analyzed. The results showed a significant difference in the reduction of depression scores between herbal medicine and Western medicine (SMD: -1.07, 95% CI: -1.40 to -0.74, *p* < 0.001; **Figure 3B**). There was a high heterogeneity in the results (*I* ^2^ = 88%).

To compare the effects of herbal medicine with those of Western medicine on anxiety in patients with cardiac neurosis, 17 studies involving 1379 patients were analyzed. The results showed a significant difference in the reduction of anxiety scores between herbal medicine and Western medicine (SMD: -0.85, 95% CI: -1.13 to -0.58, *p* < 0.001; **Figure 3C**). There was a high heterogeneity in the results (*I* ^2^ = 83%).

**Figure 4:**
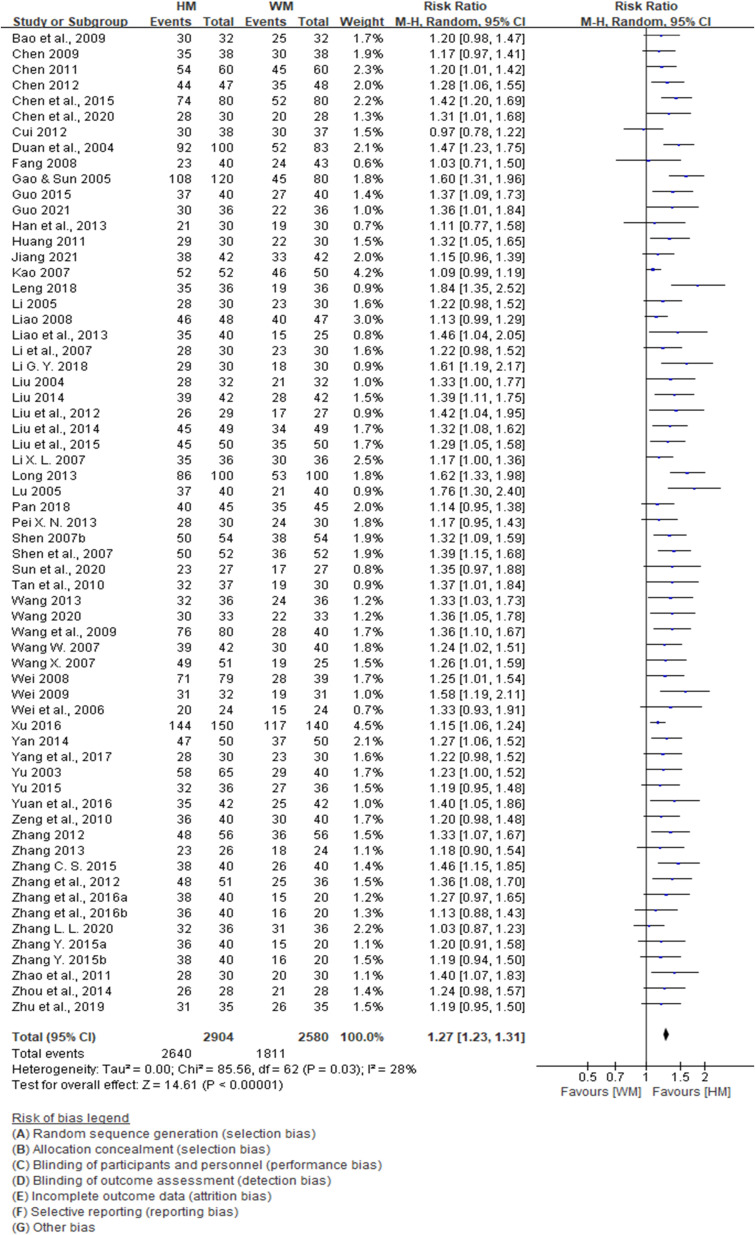
Forest plot showing the effect of herbal medicine versus Western medicine. Forest plot for effective rates.

To compare the effectiveness rates of herbal medicine with those of Western medicine in patients with cardiac neurosis, 63 studies involving 5584 patients were analyzed. The results showed a significant difference in the effectiveness rates between herbal medicine and Western medicine (RR: 1.27, 95% CI: 1.23 to 1.31, *p* < 0.001; **Figure 4**). There was a low heterogeneity in the results (*I* ^2^ = 28%).

To compare the effects of herbal medicine and Western medicine on adverse effect rates in patients with cardiac neurosis, five studies involving 648 patients were analyzed. The results showed a significant difference in the adverse effect rates between herbal medicine and Western medicine (RR: 0.22, 95% CI: 0.13 to 0.35, *p* < 0.001; **Figure 3D**). Heterogeneity was not observed (*I* ^2^ = 16%).

**Figure 5:**
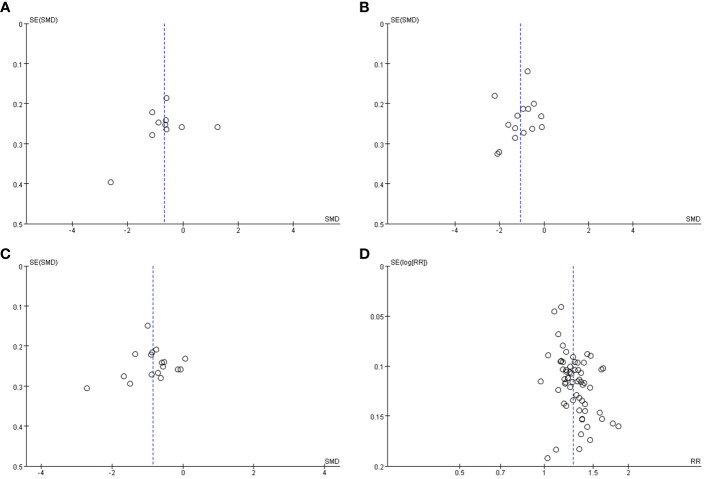
Funnel plot of the studies comparing herbal medicine with Western medicine. **(A)** Funnel plot for somatization, **(B)** funnel plot for depression **(C)** funnel plot for anxiety, and **(D)** funnel plot for effective rate.

When checking the funnel plot, publication bias was not noticeable in other variables but was suspected in the effectiveness rates (**Figure 5**).

#### 3.3.2 Herbal medicine + Western medicine vs. Western medicine

To compare the effects of combined treatment of herbal medicine and Western medicine with those of Western medicine alone on somatization in patients with cardiac neurosis, five studies involving 469 patients were analyzed. The results showed a significant difference in the reduction of somatization scores between the combined treatment of herbal medicine and Western medicine and the treatment of Western medicine alone (SMD: -0.24, 95% CI: -0.44 to -0.05, *p* = 0.01; **Supplementary Figure 20A**). There was a high heterogeneity in the results (*I* ^2^ = 97%).

To compare the effects of combined treatment of herbal medicine and Western medicine with those of Western medicine alone on depression in patients with cardiac neurosis, 15 studies involving 1,372 patients were analyzed. The results showed a significant difference in the reduction of the depression score between the combined treatment of herbal medicine and Western medicine and the treatment of Western medicine alone (SMD: -0.88, 95% CI: -1.21 to -0.54, *p* < 0.001; **Supplementary Figure 20B**). There was a high heterogeneity in the results (*I* ^2^ = 88%).

To compare the effects of combined treatment of herbal medicine and Western medicine with those of Western medicine alone on anxiety in patients with cardiac neurosis, 16 studies involving 1364 patients were analyzed. The results showed a significant difference in the reduction of anxiety scores between the combined treatment of herbal medicine and Western medicine and the treatment of Western medicine alone (SMD: -1.43, 95% CI: -1.21 to -0.54, *p* < 0.001; **Supplementary Figure 20C**). There was a high heterogeneity in the results (*I* ^2^ = 93%).

To compare the effectiveness rates of combined treatment of herbal medicine and Western medicine with those of Western medicine alone in patients with cardiac neurosis, 46 studies involving 3743 patients were analyzed. The results showed a significant difference in the effectiveness rates between the combined treatment of herbal medicine and Western medicine and the treatment of Western medicine alone (RR: 1.24, 95% CI: 1.20 to 1.28, *p* < 0.001; **Supplementary Figure 21E**). Heterogeneity was not observed (*I* ^2^ = 16%).

To compare the effects of combined treatment of herbal medicine and Western medicine with those of Western medicine alone on the adverse effect rates in patients with cardiac neurosis, 11 studies involving 847 patients were analyzed. The results showed a significant difference in the adverse effect rates between the combined treatment of herbal medicine and Western medicine and the treatment of Western medicine alone (RR: 0.38, 95% CI: 0.27 to 0.52, *p* < 0.001; **Supplementary Figure 20D**). Heterogeneity was not observed (*I* ^2^ = 0%).

When checking the funnel plot, publication bias was not noticeable in other variables but was suspected in the effectiveness rates (**Supplementary Figure 22**).

#### 3.3.3 Herbal medicine + acupuncture vs. Western medicine

To compare the effects of the combined treatment of herbal medicine and acupuncture with those of Western medicine alone on somatization in patients with cardiac neurosis, a study involving 157 patients was analyzed. The results showed a significant difference in the reduction of somatization scores between the combined treatment of herbal medicine and acupuncture and the treatment of Western medicine alone (MD: -7.64, 95% CI: -9.48 to -5.80, *p* < 0.001; **Supplementary Figure 23A**).

To compare the effects of combined treatment of herbal medicine and acupuncture with those of Western medicine alone on depression in patients with cardiac neurosis, two studies involving 229 patients were analyzed. The results showed a significant difference in the reduction of depression scores between the combined treatment of herbal medicine and acupuncture and the treatment of Western medicine alone (MD: -4.27, 95% CI: -5.13 to -3.42, *p* < 0.001; **Supplementary Figure 23B**). Heterogeneity was not observed (*I* ^2^ = 0%).

To compare the effects of combined treatment of herbal medicine and acupuncture with those of Western medicine alone on anxiety in patients with cardiac neurosis, two studies involving 229 patients were analyzed. The results showed a significant difference in the reduction of anxiety scores between the combined treatment of herbal medicine and acupuncture and the treatment of Western medicine alone (MD: -3.98, 95% CI: -4.99 to -2.96, *p* < 0.001; **Supplementary Figure 23C**). There was a high heterogeneity in the results (*I* ^2^ = 76%).

To compare the effectiveness rates of the combined treatment of herbal medicine and acupuncture with those of Western medicine alone in patients with cardiac neurosis, two studies involving 229 patients were analyzed. The results showed a significant difference in the effectiveness rates between the combined treatment of herbal medicine and acupuncture and the treatment of Western medicine alone (RR: 1.42, 95% CI: 1.13 to 1.79, *p* < 0.001; **Supplementary Figure 23D**). Moderate heterogeneity was observed (*I* ^2^ = 71%).

#### 3.3.4 Herbal medicine + psychotherapy vs. Western medicine + psychotherapy

To compare the effects of combined treatment of herbal medicine and psychotherapy with those of combined treatment of Western medicine and psychotherapy on depression in patients with cardiac neurosis, three studies involving 360 patients were analyzed. The results showed a significant difference in the reduction of depression scores between the combined treatment of herbal medicine and psychotherapy and the combination treatment of Western medicine and psychotherapy (SMD: -0.55, 95% CI: -0.76 to -0.34, *p* < 0.001; **Supplementary Figure 24A**). There was a low heterogeneity in the results (*I* ^2^ = 44%).

To compare the effects of combined treatment of herbal medicine and psychotherapy with those of combined treatment of Western medicine and psychotherapy on anxiety in patients with cardiac neurosis, four studies involving 566 patients were analyzed. The results showed a significant difference in the reduction of anxiety scores between the combined treatment of herbal medicine and psychotherapy and the combination treatment of Western medicine and psychotherapy (SMD: -0.68, 95% CI: -0.86 to -0.50, *p* < 0.001; **Supplementary Figure 24B**). There was a high heterogeneity in the results (*I* ^2^ = 98%).

To compare the effectiveness rates of combined treatment of herbal medicine and psychotherapy with those of combined treatment of Western medicine and psychotherapy in patients with cardiac neurosis, eight studies involving 806 patients were analyzed. The results showed a significant difference in the effectiveness rates between the combined treatment of herbal medicine and psychotherapy and the combination treatment of Western medicine and psychotherapy (RR: 1.23, 95% CI: 1.15 to 1.31, *p* < 0.001; **Supplementary Figure 24C**). There was a high heterogeneity in the results (*I* ^2^ = 91%).

To compare the effects of combined treatment of herbal medicine and psychotherapy with those of combined treatment of Western medicine and psychotherapy on the adverse effect rates in patients with cardiac neurosis, one study including 206 patients was analyzed. The results showed that there was no significant difference in the adverse effect rates between the combined treatment of herbal medicine and psychotherapy and the combined treatment of Western medicine and psychotherapy (RR: 4.72, 95% CI: 0.23 to 97.11, *p* = 0.31; **Supplementary Figure 24D**).

#### 3.3.5 Herbal medicine + psychotherapy + Western medicine vs. Western medicine + psychotherapy

To compare the effects of combined treatment of herbal medicine, psychotherapy, and Western medicine with those of combined treatment of Western medicine and psychotherapy on depression in patients with cardiac neurosis, three studies involving 377 patients were analyzed. The results showed a significant difference in the reduction of depression scores between the combined treatment of herbal medicine, psychotherapy, and Western medicine and the combined treatment of Western medicine and psychotherapy (SMD: -1.32, 95% CI: -1.55 to -1.10, *p* < 0.001; **Supplementary Figure 25A**). There was a high heterogeneity in the results (*I* ^2^ = 75%).

To compare the effects of the combined treatment of herbal medicine, psychotherapy, and Western medicine with those of combined treatment of Western medicine and psychotherapy on anxiety in patients with cardiac neurosis, three studies involving 377 patients were analyzed. The results showed a significant difference in the reduction of anxiety scores between the combined treatment of herbal medicine, psychotherapy, and Western medicine and the combined treatment of Western medicine and psychotherapy (SMD: -0.74, 95% CI: -0.95 to -0.53, *p* < 0.001; **Supplementary Figure 25B**). There was a low heterogeneity in the results (*I* ^2^ = 46%).

To compare the effectiveness rates of the combined treatment of herbal medicine, psychotherapy, and Western medicine with those of the combined treatment of Western medicine and psychotherapy in patients with cardiac neurosis, nine studies involving 1,094 patients were analyzed. The results showed a significant difference in the effectiveness rates between the combined treatment of herbal medicine, psychotherapy, and Western medicine and the combined treatment of Western medicine and psychotherapy (RR: 1.18, 95% CI: 1.13 to 1.24, *p* < 0.001; **Supplementary Figure 25C**). There was a high heterogeneity in the results (*I* ^2^ = 86%).

To compare the effects of the combined treatment of herbal medicine, psychotherapy, and Western medicine with those of the combined treatment of Western medicine and psychotherapy on the adverse effect rates in patients with cardiac neurosis, two studies involving 260 patients were analyzed. The results showed a significant difference in the adverse effect rates between the combined treatment of herbal medicine, psychotherapy, and Western medicine and the combined treatment of Western medicine and psychotherapy (RR: 0.15, 95% CI: 0.05 to 0.40, *p* < 0.001; **Supplementary Figure 25D**). Heterogeneity was not observed (*I* ^2^ = 0%).

#### 3.3.6 Acupuncture vs. Western medicine

To compare the effects of acupuncture with those of Western medicine on somatization in patients with cardiac neurosis, four studies involving 245 patients were analyzed. The results showed a significant difference in the reduction of somatization scores between acupuncture and Western medicine (SMD: -0.46, 95% CI: -0.72 to -0.20, *p* < 0.001; **Supplementary Figure 26A**). There was a high heterogeneity in the results (*I* ^2^ = 76%).

To compare the effects of acupuncture with those of Western medicine on depression in patients with cardiac neurosis, three studies involving 120 patients were analyzed. The results showed a significant difference in the reduction of depression scores between acupuncture and Western medicine (MD: -2.02, 95% CI: -3.10 to -0.94, *p* < 0.001; **Supplementary Figure 26B**). There was a high heterogeneity in the results (*I* ^2^ = 84%).

To compare the effects of acupuncture with those of Western medicine on anxiety in patients with cardiac neurosis, five studies involving 305 patients were analyzed. The results showed a significant difference in the reduction of anxiety scores between acupuncture and Western medicine (MD: -2.18, 95% CI: -3.21 to -1.16, *p* < 0.001; **Supplementary Figure 26C**). There was a high heterogeneity in the results (*I* ^2^ = 73%).

To compare the effectiveness rates of acupuncture with those of Western medicine in patients with cardiac neurosis, nine studies involving 746 patients were analyzed. The results showed a significant difference in the effectiveness rates between acupuncture and Western medicine (RR: 1.22, 95% CI: 1.13 to 1.31, *p* < 0.001; **Supplementary Figure 26D**). Heterogeneity was not observed (*I* ^2^ = 8%).

To compare the effects of acupuncture and Western medicine on the adverse effect rates in patients with cardiac neurosis, one study involving 60 patients was analyzed. The results showed that there was no significant difference in the adverse effect rates between acupuncture and Western medicine (RR: 0.67, 95% CI: 0.21 to 2.13, *p* = 0.49; **Supplementary Figure 26E**).

#### 3.3.7 Acupuncture + Western medicine vs. Western medicine

To compare the effects of combined treatment of acupuncture and Western medicine with those of Western medicine alone on somatization in patients with cardiac neurosis, two studies involving 332 patients were analyzed. The results showed a significant difference in the reduction of somatization scores between the combined treatment of acupuncture and Western medicine and Western medicine alone (SMD: -2.13, 95% CI: -2.42 to -1.84, *p* < 0.001; **Supplementary Figure 27A**). There was a high heterogeneity in the results (*I* ^2^ = 99%).

To compare the effects of combined treatment of acupuncture and Western medicine with those of Western medicine alone on depression in patients with cardiac neurosis, three studies involving 240 patients were analyzed. The results showed a significant difference in the reduction of depression scores between the combined treatment of acupuncture and Western medicine and Western medicine treatment alone (MD: -4.62, 95% CI: -5.30 to -3.93, *p* < 0.001; **Supplementary Figure 27B**). Heterogeneity was not observed (*I* ^2^ = 0%).

To compare the effects of combined treatment of acupuncture and Western medicine with those of Western medicine alone on anxiety in patients with cardiac neurosis, three studies involving 240 patients were analyzed. The results showed a significant difference in the reduction of anxiety scores between the combined treatment of acupuncture and Western medicine and Western medicine treatment alone (MD: -4.19, 95% CI: -4.87 to -3.50, *p* < 0.001; **Supplementary Figure 27C**). Heterogeneity was not observed (*I* ^2^ = 0%).

To compare the effectiveness rates of combined treatment of acupuncture and Western medicine with those of Western medicine alone in patients with cardiac neurosis, four studies involving 452 patients were analyzed. The results showed a significant difference in the effectiveness rates between the combined treatment of acupuncture and Western medicine and Western medicine alone (RR: 1.30, 95% CI: 1.17 to 1.46, *p* < 0.001; **Supplementary Figure 27D**). There was a high heterogeneity in the results (*I* ^2^ = 76%).

To compare the effects of combined treatment of acupuncture and Western medicine with those of Western medicine alone on the adverse effect rates in patients with cardiac neurosis, one study involving 60 patients was analyzed. The results showed that there was no significant difference in the adverse effect rates between the combined treatment of acupuncture and Western medicine and Western medicine treatment alone (RR: 0.83, 95% CI: 0.28 to 2.44, *p* = 0.33; **Supplementary Figure 27E**).

## 4 Discussion

### 4.1 Principal findings

To develop clinical practice guidelines for autonomic dysfunction in Korean medicine, this study conducted a systematic review and meta-analysis targeting cardiac neurosis, which reflects the oriental medicine concept of autonomic dysfunction and for which empirical evidence can be found because of the existence of categorized diagnoses.

Most studies on Korean medicine interventions for patients with cardiac neurosis have been of poor methodological quality. In the case of selection bias, there were many unclear cases, and in performance and detection bias, most cases were at a high risk.

Compared to Western medicine, herbal medicine was more effective in somatization, depression, anxiety, and the effectiveness rates and was lower in adverse effect rates. Combined treatment with herbal medicine and Western medicine showed similar results. Compared to Western medicine, the combined treatment of herbal medicine and acupuncture was more effective in somatization, depression, anxiety, and the effectiveness rates. Compared to the combined treatment of Western medicine and psychotherapy, the combined treatment of herbal medicine and psychotherapy and that of herbal medicine, Western medicine, and psychotherapy were more effective in terms of depression, anxiety, and the effectiveness rates. However, while the combined treatment of herbal medicine, Western medicine, and psychotherapy had a lower adverse effect rate than the combined treatment of Western medicine and psychotherapy, there was no difference in the rate of adverse effects between the combined treatment of herbal medicine and psychotherapy and the combined treatment of Western medicine and psychotherapy.

Compared to Western medicine, acupuncture was more effective for somatization, depression, anxiety, and the effectiveness rates, but there was no difference in the rate of adverse effects. These results were also observed in the combined treatment of acupuncture and Western medicine.

In summary, in patients with cardiac neurosis, herbal medicine, the combined treatment of herbal medicine and Western medicine, combined treatment of herbal medicine and acupuncture, acupuncture, and combined treatment of acupuncture and Western medicine showed better effects than Western medicine alone. Overall, the combined treatment of herbal medicine and psychotherapy and that of herbal medicine, psychotherapy, and Western medicine showed better results than the combined treatment of Western medicine and psychotherapy.

When publication bias was checked through a funnel plot, it was suspected only for the effectiveness rate variable in the comparison between Western medicine and herbal medicine, and between the combined treatment of herbal medicine and Western medicine and Western medicine alone.

These results can also be compared to those of previous studies that reviewed the effects of Korean medicine treatment for cardiac neurosis. In a study by Li et al. (13), acupuncture treatment had a better effect than Western medicine on the effectiveness rates and anxiety, and the difference in effect was not significant for physical symptoms, somatization, depression, or the adverse effect rates. These results were compared to those of the present study, which showed significant effects on somatization and depression. This difference appears to have occurred because this study included both normal acupuncture and electroacupuncture as well as various measures of somatization rather than a single measure. Meanwhile, in a study by Wang et al. (14), herbal medicine treatment containing Radix Bupleuri had better effects on the effectiveness rates, anxiety, depression, and adverse effect rates than Western medicine containing anti-inflammatory drugs. These findings are similar to the results of the present study.

### 4.2 Limitations

Originally, this study intended to include a wider range of Korean medicine treatments such as herbal medicine preparations other than decoctions, moxibustion, cupping, herbal acupuncture, manual therapy, and self-management methods. However, there was a limitation in that only specific treatment methods were analyzed because of the lack of studies.

This review had a broad scope of intervention and assumed heterogeneity between studies. There was significant heterogeneity depending on the intervention method and variables. However, we were unable to conduct a moderating effect analysis for this large heterogeneity. Future research could determine whether the effect of treatment varies depending on the characteristics of the intervention method (e.g., type or duration of prescription in herbal medicine treatment, acupuncture points in acupuncture treatment, and type of psychotherapy).

Different diagnostic criteria across studies may also have contributed to the heterogeneity in effect sizes. The diagnostic criteria for cardiac neurosis are mostly similar; however, there are specific differences when broadly divided into Western and Chinese diagnostic criteria. Future research should confirm these differences according to these diagnostic criteria.

In some studies, the addition or subtraction of herbal medicines or acupoints according to patient patterns may have contributed to the heterogeneity of the effect size. Future research could reveal differences according to these patterns or obtain a more precise effect size by excluding studies that consider the pattern.

The poor methodological quality of the studies included in this review may also be another limitation. There were some cases where a specific randomization method was not specified, allocation concealment and blinding methods were not mentioned or were at a high risk of bias, or publication bias was suspected in some variables. To ensure the reliability and validity of the research, studies with a high risk of bias were removed and analyzed. However, in this study, considering that bias inevitably occurred due to the nature of Korean medicine research, and the simple descriptive practices of Chinese medicine research were taken into consideration, high-risk studies were also included in the analysis.

Finally, another limitation is that it is somewhat difficult to generalize the results due to the insufficient number of studies measuring the side effects of Korean medicine interventions. In this study, Korean medicine interventions were found to have a lower incidence of side effects than Western medicine, and the side effects that occurred were not serious. However, sufficient data on side effects has not been obtained and biochemical indicators such as blood tests have not been confirmed. Therefore, when using Korean medicine interventions, these limitations need to be considered and side effects carefully monitored.

A recent study investigated side effects of herbal medicine by text mining approximately 5,700 articles. Most side effects appear as mild symptoms, but in rare cases, serious side effects such as liver damage or pneumonia have occurred. These side effects were mainly experienced in elderly patients over the age of 60 and male patients, but for accurate comparison, information or statistics on the population using herbal medicine are needed (15). Unlike Western medicine, which expects therapeutic efficacy from a single compound, Chinese and Korean herbal medicine prescriptions contain many compounds as constituents, showing diverse and complex effects on patients. In addition, it is difficult to specify the cause of side effects due to lack of standardization of the quality of herbal medicine, medicinal material source, and dosage method. Therefore, more careful attention should be paid to side effects when prescribing herbal medicine, and this should be reflected in future studies.

### 4.3 Clinical implications

This systematic review and meta-analysis has potential clinical value. First, compared to the use of existing drug treatments in patients with cardiac neurosis, the use of herbal medicine was shown to be a more clinically effective intervention with fewer adverse effects. Acupuncture treatment did not significantly improve the adverse effect rates but was more effective in terms of the effectiveness rates. Therefore, herbal medicine can be considered a priority for the treatment of cardiac neurosis, which combines mental symptoms such as depression and anxiety with physical symptoms centered on circulatory system symptoms, and can be considered in combination with other treatments such as acupuncture, Western medicine, and psychotherapy, depending on the symptoms.

Cardiac neurosis is accompanied by psychological disorders, but its core symptoms can be considered to be cardiothoracic symptoms. Therefore, acupuncture can be a direct and useful treatment method for relieving physical discomfort. In this study, most treatments were performed using normal rather than special acupuncture, and the acupuncture points of the heart, pericardium, bladder, and governing vessel meridians were mainly used. Common acupuncture points include Xinshu (BL15), Jueyinshu (BL14), Neiguan (PC6), Shenmen (HT7), Juque (CV14), and Danzhong (CV17), which can relieve discomfort in the chest and abdomen, which are the main symptoms, in addition to stabilizing the mind and body.

Some studies included in this analysis had methodological quality problems. In addition, it is insufficient to know how four diagnostic methods (四診) such as watching, smelling, inquiring, and cutting (望聞問切) were used when using the Korean medicine diagnostic criteria, or what algorithm was used to perform the Korean medicine pattern identification to select and add or subtract the prescription. This affects the validity and reliability of the results of this study and limits their clinical use.

However, the results of this meta-analysis revealed the effectiveness of Korean medicine treatment for cardiac neurosis, and it was so significant that it revealed the effectiveness of both a single treatment and combination treatment of various Korean medicine tools. In addition, this study supplemented the evidence through a systematic review and meta-analysis of cardiac neurosis, showing that the disease concept of traditional medicine can be organized through the latest research. This will provide an opportunity to gain a new understanding of various disease concepts in oriental medicine. We hope that this disease concept of traditional medicine will be widely used in clinical practice.

### 4.4 Conclusion

Currently, diseases with various traditional concepts are treated in Korean medicine clinical settings; however, they are usually not included in the standard diagnostic system. Therefore, research on this topic is limited, and it is easy to fall into a vicious cycle in which standardization of the disease becomes more difficult. Accordingly, to develop clinical practice guidelines for autonomic dysfunction in Korean medicine, this study conducted a systematic review and meta-analysis targeting cardiac neurosis, which reflects the concept of autonomic dysfunction and has a categorized diagnosis. The results revealed the effectiveness of Korean medicine treatment for cardiac neurosis, as well as the effectiveness of both a single treatment and a combination treatment using various Korean medicine tools. This study can be seen as the beginning of research on the disease concept in oriental medicine. Based on this study, we hope that related follow-up research and oriental medicine interventions for traditional diseases will be actively conducted.

## Data availability statement

The raw data supporting the conclusions of this article will be made available by the authors, without undue reservation.

## Author contributions

H-YP : Writing – original draft, Writing – review & editing. HWL : Writing – review & editing. G-JS : Writing – original draft. SH : Writing – review & editing. SH : Writing – review & editing. H-WS: Conceptualization, Methodology, Writing – review & editing. S-IY : Writing – original draft. CP : Writing – original draft. S-YC : Supervision, Writing – review & editing. JWK : Conceptualization, Project administration, Writing – review & editing.
