# Supplementary material for: Systematic review and meta-analysis of cardiac neurosis for development of clinical practice guidelines of Korean medicine
Source: Front Psychiatry. 2024 Feb 12;15:1302245. doi: 10.3389/fpsyt.2024.1302245 (PMC10895026; doi:10.3389/fpsyt.2024.1302245)
Supplement: Supplementary file 1 [file DataSheet_1.docx]

Supplementary Material

Effect of Korean medicine interventions on cardiac neurosis: A systematic review and meta-analysis

Hui-Yeong Park^†^, Hyun Woo Lee^†^, Geum-Ju Song, Sunggyu Hong, Sunghee Hong, Hyo-Weon Suh, Seok-In Yoon, Chan Park, Sun-Yong Chung, Jong Woo Kim^*^

*** Correspondence:** Jong Woo Kim: [aromaqi@khu.ac.kr](mailto:aromaqi@khu.ac.kr)

# Supplementary Figures and Tables

## Supplementary Tables

**Supplementary Table 1.** Characteristics of included articles.

|  | **PubMed** | | **CENTRAL** | | | **Embase (Ovid)** | |
| --- | --- | --- | --- | --- | --- | --- | --- |
| #1 | Neurocirculatory Asthenia[MeSH Terms] | | MeSH descriptor: [Neurocirculatory Asthenia] explode all trees | | | exp cardiac anxiety/ | |
| #2 | "Da Costa syndrome"[Title/Abstract] | | ("Da Costa syndrome"):ti,ab,kw | | | "Da Costa syndrome".mp. | |
| #3 | "Da Costa syndrome"[Title/Abstract] | | ("Da Costa's syndrome"):ti,ab,kw | | | "Da Costa's syndrome".mp. | |
| #4 | "cardiovascular neurosis"[Title/Abstract] | | ("cardiovascular neurosis"):ti,ab,kw | | | "cardiovascular neurosis".mp. | |
| #5 | "cardiac neurosis"[Title/Abstract] | | ("cardiac neurosis"):ti,ab,kw | | | "cardiac neurosis".mp. | |
| #6 | #1 OR #2 OR #3 OR #4 OR #5 | | #1 OR #2 OR #3 OR #4 OR #5 | | | 1 or 2 or 3 or 4 or 5 | |
|  | | **CiNii** | | | **CNKI (cnki.net) (专业检索-中英文扩展)** | | |
| Cardiac neurosis | | 心臓神経症 OR "cardiac neurosis" | | | SU='心血管神经官能症'+'心脏神经官能症' | | |
| Cardiac neurosis + herbal medicine | | “traditional Korean medicine” OR “traditional Chinese medicine” OR “Traditional oriental medicine” OR “Kampo medicine” OR herb OR decoction OR botanic OR 漢方薬OR ハーブ OR 散 OR 汤OR 丸 | | | SU='中药'+'中医药'+'本草'+'散'+'汤'+'丸'+'方'+'颗粒'+'胶囊'+'自拟' | | |
| Cardiac neurosis + acupuncture/manual therapy | | acupuncture OR moxibustion OR moxa OR cupping OR pharmacopuncture OR shiatsu OR anma OR 鍼 OR 灸 OR 吸角 OR 指圧OR 按摩 | | | SU='针'+'针刺'+'针灸'+'灸'+'罐'+'穴位注射'+'推拿'+'按摩'+'指压'+'按压' | | |
| Cardiac neurosis + psychotherapy/self-management method | | psychotherapy OR "cognitive behavioral therapy" OR "cognitive therapy" OR "autogenic training" OR "progressive muscle relaxation" OR qigong OR meditation OR yoga OR 心理療法 OR 認知行動療法 OR 認知療法 OR 自律訓練法 OR 漸進的筋弛緩法 OR 気功 OR 瞑想 OR ヨガ | | | SU='心理治疗'+'精神治疗'+'认知行为疗法'+'认知治疗'+'自生训练'+'肌肉放松'+'放松训练'+'气功'+'冥想'+'正念'+'瑜伽' | | |
|  | | **Kmbase** | | **ScienceON** | **KISS** | | **OASIS** |
| Cardiac neurosis | | 심장신경증 | | 심장신경증 | 심장신경증 | | 심장신경증 |

**Supplementary Table 2.** Characteristics of included articles.

| **Article label** | **Country** | **Age, mean (SD)** | **Female, %** | **Diagnostic criteria** | **Intervention** | | **Intervention** | | **Course** | **Type of outcome** |
| --- | --- | --- | --- | --- | --- | --- | --- | --- | --- | --- |
|  |  |  |  |  | **Treatment** | **n** | **Treatment** | **n** |  |  |
| Bai et al., 2017 (1) | China | 75.5 (N/R) | 57 | N/R | Herb (Shensongyangxin-capsule, [参松养心胶囊](https://blog.naver.com/hdjakg/222655592318)) + WM | 71 | WM | 70 | 4Ws | ②③④ |
| Bao et al., 2009 (2) | China | Range 30~65 | 47 | DCTS | Herb (Danchisoyo-san, 丹梔逍遙散) | 32 | WM | 32 | 1M | ④ |
| Cao et al., 2010 (3) | China | 38.6 (N/R) | 54 | Criterion A | Herb (Yixinshu-capsule, [益心舒胶囊](https://blog.naver.com/hdjakg/222582132938)) + WM | 100 | WM | 100 | N/R | ④⑤ |
| Che 2017 (4) | China | TG 43.22 (2.32)  CG 43.31 (2.51) | 72 | PIM | Herb (Wenxin-granule, [稳心颗粒](https://blog.naver.com/hdjakg/222581067263)) + WM | 29 | WM | 29 | 2Ms | ④ |
| Chen 2009 (5) | China | TG 34.2 (10.7)  CG 35.6 (9.7) | 59 | PIM | Herb (Gaeulsunsimtang-gagam, 开郁顺心汤加減) | 38 | WM | 38 | 4Ws | ④ |
| Chen 2011 (6) | China | TG 41 (N/R)  CG 39 (N/R) | 63 | PIM-12 | Herb (Yangsimseoul-tang, 养心舒郁汤) | 60 | WM | 60 | 30Ds | ①④ |
| Chen 2012 (7) | China | N/R | 67 | PIM, TCTSP, CIM | Herb (Yeongsimansin-tang, 宁心安神汤) | 47 | WM | 48 | 30Ds | ④ |
| Chen 2014 (8) | China | 34.17 (N/R) | 84 | N/R | Herb (Shensongyangxin-capsule, [参松养心胶囊](https://blog.naver.com/hdjakg/222655592318)) + Psy | 29 | WM + Psy | 29 | N/R | ④ |
| Chen 2018 (9) | China | TG 35 (N/R)  CG 37.5 (N/R) | N/R | CIM | Herb (Zaorenningxin-capsule, 枣仁宁心胶囊) + WM | 15 | WM | 15 | 1W | ④ |
| Chen et al., 2015 (10) | China | TG 36~82  CG 38~79 | 71 | Criterion A | Herb (Ansinyangsimhwan-gagam, 安神养心丸[加減](https://ko.dict.naver.com/#/entry/koko/e34a58aacfe24f45802bcd2b53094a83)) | 80 | WM | 80 | 2~3Ms | ④ |
| Chen et al., 2018 (11) | China | TG 48.62 (17.52)  CG 50.26 (13.91) | 65 | PIM-14, CIM-7 | Herb (Seunghamtang-hap-gyejibongnyeonghwan-gagam, [升陷湯](https://www.koreantk.com/ktkp2014/prescription/prescription-view.view?preCd=P0019800)+桂枝茯苓丸[加減](https://ko.dict.naver.com/#/entry/koko/e34a58aacfe24f45802bcd2b53094a83)) + Psy | 40 | WM + Psy | 40 | 4Ws | ②③④ |
| Chen et al., 2020 (12) | China | Range 28~55 | 50 | Guidelines for the Diagnosis and Treatment of Cardiovascular Diseases, GCNT | Herb (Geonsimpyeongyul-hwan, 健心平律丸) | 30 | WM | 28 | 12Ws | ④ |
| Chu 2011 (13) | China | 49.8 (N/R) | 75 | CCMD-3 | Herb (Wenxin-granule, [稳心颗粒](https://blog.naver.com/hdjakg/222581067263)) + WM + Psy | 31 | WM + Psy | 24 | 4Ws | ④ |
| Cui 2012 (14) | China | 41.6 (7.6) | 71 | PIM-11 | Herb (Sihosogansan-gagam, 柴胡消奸散[加減](https://ko.dict.naver.com/#/entry/koko/e34a58aacfe24f45802bcd2b53094a83)) | 38 | WM | 37 | 8Ws | ②③④ |
|  |  |  |  |  | Herb (Sihosogansan-gagam, 柴胡消奸散[加減](https://ko.dict.naver.com/#/entry/koko/e34a58aacfe24f45802bcd2b53094a83)) + WM | 40 | WM | 37 | 8Ws | ②③④ |
| Deng et al., 2017 (15) | China | 31.56 (N/R) | 61 | N/R | Herb (Shensongyangxin-capsule, [参松养心胶囊](https://blog.naver.com/hdjakg/222655592318)) + WM | 31 | WM | 31 | 4Ws | ④ |
| Du et al., 2007 (16) | China | 35.5 (N/R) | 59 | PIM | Herb (Soyo-hwan and Guibi-hwan, [逍遥丸](https://blog.naver.com/hdjakg/222805455701)+歸脾丸) | 54 | WM | 54 | 6Ws | ④ |
| Duan et al., 2014 (17) | China | 33.5 (N/R) | 66 | PIM-10 | Herb (Soyosan-gagam. 逍遙散[加減](https://ko.dict.naver.com/#/entry/koko/e34a58aacfe24f45802bcd2b53094a83)) | 100 | WM | 83 | 6Ws | ④ |
| Fang 2008 (18) | China | Range 30~55 (N/R) | 54 | National higher medical college textbooks-5 | Herb (Soyosan-gagam. 逍遙散加減) | 40 | WM | 43 | 8Ws | ④ |
| Fang et al., 2013 (19) | China | 54.5 (N/R) | 100 | N/R | Herb (Wenxin-granule, [稳心颗粒](https://blog.naver.com/hdjakg/222581067263)) + WM | 60 | WM | 58 | 1M | ④ |
| Fu et al., 2006 (20) | China | 46 (N/R) | 72 | PIM-11, Guidelines for Psychiatric and Nervous Diseases in China | Herb (Soyo-san, 逍遙散) + WM | 25 | WM | 25 | 8Ws | ②③④ |
| Gao, Liu et al., 2005 (21) | China | 37.5 (10.7) | 77 | CCMD-3 | Herb (Tongxinluo-capsule, [通心絡](http://www.dailymedi.com/detail.php?number=809467&thread=22r05)胶囊) | 30 | WM | 30 | 4Ws | ② |
| Gao & Sun, 2005 (22) | China | 38 (N/R) | 62 | Criterion A | Herb (Gamisoyohwan-hap-xintongkoufuye, 加味[逍遥丸](https://blog.naver.com/hdjakg/222805455701)+心通口服液) | 120 | WM | 80 | N/R | ④ |
| Gao et al., 2008 (23) | China | 38.33 (5.12) | 60 | Criterion A | Herb (Ikgichongmyeongtang-gagam, 益氣聰明湯加減) + WM | 42 | WM | 38 | 2Ws | ④ |
| Guo 2015 (24) | China | TG 48.1 (11.5)  CG 49.5 (12.0) | 61 | N/R | Herb (Haeulyeongsimtang-gagam, 解郁宁心汤加減) | 40 | WM | 40 | 4Ws | ④ |
| Guo 2021 (25) | China | 44 (N/R) | 64 | PIM, GCNT, DTGCT | Herb (Wenxinyihao, [稳心](https://blog.naver.com/hdjakg/222581067263) 1号) | 36 | WM | 36 | 4Ws | ①②③④ |
| Guo et al., 2020 (26) | China | TG 22~51  CG 21~53 | 61 | PIM, DCTS | Herb (Banhachulmi-hap-ijihwan-gagam, 半夏秫米合二至丸加減) + WM | 36 | WM | 36 | 8Ws | ②③④ |
| Han 2018 (27) | China | TG 36.1 (2.7)  CG 36.3 (2.6) | 79 | Unable to verify | Herb (Gijukjihwang-hwan, 杞竹地黄丸) + WM | 41 | WM | 41 | 1M | ④ |
| Han et al., 2013 (28) | China | 37 (6.5) | 68 | PIM, DCTS | Herb (Jeongsimtang-gagam, 正心湯加減) | 30 | WM | 30 | 4Ws | ①②③④ |
| Hu et al., 1994 (29) | China | 33.83 (N/R) | 91 | CCIC | NA | 44 | WM | 44 | 10Ds | ④ |
| Huang 2011 (30) | China | 36 (6.8) | 63 | Criterion A | Herb (Soyosan-gagam and Wenxin-granule, 逍遙散加減+[稳心颗粒](https://blog.naver.com/hdjakg/222581067263)) | 30 | WM | 30 | 60Ds | ②③④ |
| Huang et al., 2016 (31) | China | 45.2 (N/R) | 66 | PIM | Herb (Baekjayangsim-hwan, 栢子養心丸) + WM | 25 | WM | 25 | 8Ws | ④⑤ |
| Huo 2016 (32) | China | TG 38.66 (13.24)  CG 36.42 (15.31) | 97 | IM-7, CIM, GCNT | Herb (Soyosan-hap-guibitang-gagam, 逍遙散 +歸脾湯加減) + Psy | 50 | WM + Psy | 41 | 4Ws | ④ |
| Jiang 2021 (33) | China | TG 33.59 (4.55)  CG 34.65 (3.21) | 55 | PIM, GCNT, DCTS | Herb (Sogangeonbi-tang, 疏肝健脾汤) | 42 | WM | 42 | 4Ws | ②③④⑤ |
| Kao 2007 (34) | China | Range 18~65 | 69 | IM, CCMD-3, GCNT | Herb (Chaihuanxin-capsule, 柴胡安心胶囊) | 52 | WM | 50 | 4Ws | ②③④ |
| Leng 2018 (35) | China | N/R | 60 | DCTS, GCNT, PIM-7 | Herb (Yangsimsihwan-tang, 养心柴欢汤) | 36 | WM | 36 | 4Ws | ①④ |
| Li 2005 (36) | China | 47 (N/R) | 88 | PIM-11 | Herb (Kaixin-capsuleⅡ, 开心胶囊 2号) | 30 | WM | 30 | 30Ds | ④ |
| Li X. L. 2007 (37) | China | 40.55 (N/R) | 71 | IM-4 | Herb (Baoxinning-capsule, 保心宁胶囊) | 36 | WM | 36 | 1M | ④ |
| Li M. S. 2007 (38) | China | 35.2 (8.9) | 68 | CCMD-3 | Herb (Shensongyangxin-capsule, [参松养心胶囊](https://blog.naver.com/hdjakg/222655592318)) + WM | 62 | WM | 61 | 6Ws | ④ |
| Li 2012 (39) | China | 35.2 (N/R) | 54 | CCMD-3 | Herb (Yixinshu-capsule, [益心舒胶囊](https://blog.naver.com/hdjakg/222582132938)) + Psy | 106 | WM + Psy | 100 | 4Ws | ③④⑤ |
| Li 2016 (40) | China | TG 40.3 (2.9)  CG 40.1 (3.0) | 79 | Criterion D | Herb (Yangeumjeonggye-tang, 养阴定悸汤) + WM | 35 | WM | 35 | 8Ws | ④ |
| Li G. Y. 2018 (41) | China | TG 45.33 (6.85)  CG 45.27 (7.40) | 78 | PIM-14, DCTS, DT-4 | Herb (Jogangeonbiyangsim-bang, 调肝健脾养心方) | 30 | WM | 30 | 4Ws | ①②③④ |
|  |  | TG 47.73 (4.64)  CG 45.27 (7.40) | 78 | PIM-14, DCTS, DT-4 | Herb (Jogangeonbiyangsim-bang, 调肝健脾养心方) + WM | 31 | WM | 30 | 4Ws | ①②③④ |
| Li S. U. 2018 (42) | China | 35.3 (N/R) | 66 | N/R | Herb (Gyeji-gu-jagyak-ga-chokchilmoryeoyonggolguyeok-tang, 桂枝去芍藥加蜀漆牡蠣龍骨救逆湯) + WM | 37 | WM | 37 | 4Ws | ②③④ |
| Li X. B. 2021 (43) | China | 43.6 (4.5) | 41 | N/R | Herb (Wenxin-granule, [稳心颗粒](https://blog.naver.com/hdjakg/222581067263)) + WM | 34 | WM | 32 | 2Ms | ④ |
| Li J. C. 2021 (44) | China | 34 (N/R) | 56 | IM, GCNT | Herb (Saengmaegyangsim-tang, 生脉养心汤) + WM + Psy | 100 | WM + Psy | 100 | 4Ws | ④⑤ |
| Li et al., 2007 (45) | China | TG 44.71 (N/R)  CG 46.73 (N/R) | 72 | PIM, TCTSP | Herb (Kaixin-capsuleⅡ, 开心胶囊 2号) | 30 | WM | 30 | 30Ds | ①④ |
| Li & Jin 2015 (46) | China | TG 38.6 (9.1)  CG 39.0 (8.0) | 50 | N/R | Herb (Yixinshu-capsule, [益心舒胶囊](https://blog.naver.com/hdjakg/222582132938)) + WM | 52 | WM | 50 | 4Ws | ③④ |
| Li, Li et al., 2015 (47) | China | 34.5 (N/R) | 61 | IM | Herb (Soganhwalhyeoltang-gagam, 疎經活血湯加減) + WM + Psy | 55 | WM + Psy | 42 | 30Ds | ②③④ |
| Liang 2015 (48) | China | 44.22 (N/R) | 34 | CCMD | Herb (Yeongsim-tang, 宁心汤) + WM | 28 | WM | 28 | 30Ds | ④⑤ |
| Liang 2022 (49) | China | 36 (N/R) | 68 | N/R | Herb (Deungnonansim-bang, 邓老暖心方) + WM | 60 | WM | 60 | 4Ws | ①②③④ |
| Liao 2008 (50) | China | TG range 25~58  CG range 24~59 | 61 | Criterion A | Herb (Guibitang-gagam, 歸脾湯加減) | 48 | WM | 47 | 20Ds | ④ |
| Liao et al., 2013 (51) | China | Range 20~40 | 48 | MTC | Herb (Jagamchotang-gagam, 炙甘草湯加減) | 40 | WM | 25 | 10Ds | ④ |
| Liu 2004 (52) | China | Range 38~58 | 72 | PIM-10 | Herb (Wenxin-granule and Xinshenning-tablet, [稳心颗粒](https://blog.naver.com/hdjakg/222581067263)+心神宁片) | 32 | WM | 32 | 8Ws | ④ |
| Liu 2014 (53) | China | TG 48.2 (11.9)  CG 49.7 (12.6) | 71 | N/R | Herb (Haeulyeongsimtang-gagam, 解郁宁心汤加減) | 42 | WM | 42 | 1M | ④(3M f/w)⑤ |
| Liu 2016 (54) | China | TG 40.46 (5.42)  CG 41.98 (5.87) | 80 | PIM, The Guiding Principles of the Research of New Drugs of Traditional Chinese Medicine in the Treatment of Yasuo | Herb (Yangsimjeongtaek-tang, 养心定择汤) | 30 | WM | 30 | 4Ws | ①②③ |
| Liu et al., 2012 (55) | China | TG 47.5 (N/R)  CG 48.2 (N/R) | 100 | Criterion B | Herb (Hwanggigyejiyonggolmoryeotang-gagam, 黄芪桂枝龙骨牡蛎汤加减) | 29 | WM | 27 | 3Ms | ④ |
| Liu et al., 2014 (56) | China | TG 45.5 (8.2)  CG 46.1 (8.3) | 69 | PIM, DCTS | Herb (Eunsimansin-tang, 稳心安神汤) | 49 | WM | 49 | 8Ws | ②③④ |
| Liu et al., 2015 (57) | China | 34.16 (N/R) | 30 | N/R | Herb (Yeongsimansin-tang, Jaoga-tablet and Cheonwangbosim-dan, 宁心安神汤+刺五加片 + 天王補心丹) | 50 | WM | 50 | 30Ds | ④ |
| Long 2013 (58) | China | 42.6 (N/R) | 64 | PIM, DCTS | Herb (Soyo-san, 逍遙散) | 100 | WM | 100 | 20Ds | ②③④ |
| Lu 2005 (59) | China | TG 36.5 (N/R)  CG 35.6 (N/R) | 68 | CCIC | Herb (Wenxin-granule, [稳心颗粒](https://blog.naver.com/hdjakg/222581067263)) | 40 | WM | 40 | 4Ws | ④⑤ |
| Ma et al., 2009 (60) | China | 38.33 (5.1) | 62 | PIM | Herb (Gamiseunggang-san, [加味](https://ko.dict.naver.com/#/entry/koko/be3caf96ac664e458ef524a91a5272ff)升降散) + Psy | 32 | WM + Psy | 21 | 4Ws | ④ |
| Pan 2013 (61) | China | TG 39.5 (2.1)  CG 40.5 (1.9) | 58 | PIM, CCMD | Herb (Sanjointang-hap-silsosan, 酸棗仁湯+失笑散) + WM | 37 | WM | 37 | 4Ws | ②③④ |
| Pan 2018 (62) | China | TG 46.73 (4.95)  CG 47.93 (5.17) | 89 | PIM, DCTS | Herb (Yeongsim-tang, 宁心汤) | 45 | WM | 45 | 4Ws | ②③④ |
| Pei X. N. 2013 (63) | China | 36.5 (N/R) | 57 | PIM | Herb (Sahyangbosim-hwan, 麝香保心丸) | 30 | WM | 30 | 2Ms | ④ |
| Pei G. X. 2013 (64) | China | TG 42.58 (6.12)  CG 44.32 (4.58) | 65 | PIM-12, CCMD | Herb (Sihosogansan-gagam, 柴胡消奸散[加減](https://ko.dict.naver.com/#/entry/koko/e34a58aacfe24f45802bcd2b53094a83)) + WM | 60 | WM | 60 | 8Ws | ②③④ |
| Qian et al., 2020 (65) | China | TG 44.76 (11.86)  CG 46.16 (11.27) | 55 | PIM, GCNT | Herb (Soyosan-gami, 逍遙散[加味](https://ko.dict.naver.com/#/entry/koko/be3caf96ac664e458ef524a91a5272ff)) + WM | 30 | WM | 30 | 4Ws | ①④⑤ |
| Qin et al., 2013 (66) | China | 35.8 (7.6) | 68 | PIM | Herb (Wenxin-granule, [稳心颗粒](https://blog.naver.com/hdjakg/222581067263)) + Psy | 60 | WM + Psy | 60 | 8Ws | ②③④ |
|  |  |  |  |  | Herb (Wenxin-granule, [稳心颗粒](https://blog.naver.com/hdjakg/222581067263)) + WM + Psy | 60 | WM + Psy | 60 | 8Ws | ②③④ |
| Ren et al., 2010 (67) | China | TG 40.2 (N/R)  CG 38.0 (N/R) | 82 | Criterion A | NA + Chuna | 30 | WM | 30 | 33Ds | ④ |
| Ren 2018 (68) | China | N/R | 54 | PIM, CIM, GCNT | Herb (Baekapbosim-tang, 百合补心汤) + WM | 36 | WM | 36 | 4Ws | ④ |
| Shen et al., 2007 (69) | China | Range 21~58 | 69 | PIM-11 | Herb (Simmiondamtang-gagam, 十味溫膽湯加減) | 52 | WM | 52 | 30Ds | ④ |
| Shi et al., 2013 (70) | China | TG 40 (N/R)  CG 39 (N/R) | 63 | Clinical disease diagnosis basis and cure improvement criteria, PIM | Herb (Soyosan-gagam, 逍遙散加減) + WM | 45 | WM | 45 | 20Ds | ④ |
| Sue 2020 (71) | China | TG 42.53 (9.59)  CG 42.09 (10.12) | 72 | IM-8, CIM-2 | NA + Cupping | 34 | WM | 33 | 4Ws | ②③④ |
| Sun 2015 (72) | China | 34.8 (3.7) | 55 | N/R | Herb (Shensongyangxin-capsule, 参松养心胶囊) + WM | 28 | WM | 28 | 30Ds | ④⑤ |
| Sun 2022 (73) | China | 43.85 (N/R) | 89 | CCMD-3, TCM Syndrome Differentiation and Treatment of Cardiac Neurosis | Herb (Danchisoyosan-gagam, 丹梔逍遙散加減) + WM | 45 | WM | 45 | 4Ws | ②③⑤ |
| Sun et al., 2005 (74) | China | 42.4 (N/R) | 64 | IM | Herb (Simmiondamtang-gagam, 十味溫膽湯加減) + WM | 20 | WM | 19 | 30Ds | ④ |
| Sun et al., 2020 (75) | China | TG 39.52 (8.57)  CG 40.29 (7.93) | 78 | PIM-14, CIM-2 | Herb (Sihogagyejiyonggolmoryeo-tang, 柴胡加桂枝龍骨牡蠣湯) | 27 | WM | 27 | 4Ws | ③④ |
| Tan et al., 2010 (76) | China | 45 (8.8) | 69 | PIM, TCTSP, CIM | Herb (Shensongyangxin-capsule, [参松养心胶囊](https://blog.naver.com/hdjakg/222655592318)) | 37 | WM | 30 | 4Ws | ④ |
| Tang et al., 2015 (77) | China | 35.2 (4.5) | 61 | Criterion A | Herb (Yixinshu-capsule, [益心舒胶囊](https://blog.naver.com/hdjakg/222582132938)) + WM | 80 | WM | 80 | N/R | ①②④ |
| Wang 2000 (78) | China | Median 33 | 66 | PIM-12, CIM | Herb (Soyohwan-gagam, 濃縮逍遙散加減) + Psy | 20 | WM + Psy | 18 | 2Ms | ④ |
| Wang X. 2007 (79) | China | 38.6 (N/R) | 75 | MTC, CIM | Herb (Danchisoyo-san, 丹梔逍遙散) | 51 | WM | 25 | 4Ws | ④ |
| Wang W. 2007 (80) | China | 41 (N/R) | 61 | PIM, DCTS | Herb (Geollyeongtang-gagam, 建瓴湯加減) | 42 | WM | 40 | 4Ws | ④ |
| Wang 2008 (81) | China | TG 36 (N/R)  CG 34 (N/R) | 68 | Criterion E | Herb (Shensongyangxin-capsule, [参松养心胶囊](https://blog.naver.com/hdjakg/222655592318)) + WM | 45 | WM | 45 | 4Ws | ④ |
| Wang 2011 (82) | China | Median 33 | 66 | PIM-11 | Herb (Soyo-san, 逍遙散) + WM | 20 | WM | 18 | 2Ms | ④ |
| Wang 2012 (83) | China | 31.2 (N/R) | 64 | PIM-11 | Herb (Soyo-hwan, 逍遙丸) + WM | 22 | WM | 20 | 2Ms | ④ |
| Wang 2013 (84) | China | 39.95 (N/R) | 67 | N/R | Herb (Shensongyangxin-capsule, [参松养心胶囊](https://blog.naver.com/hdjakg/222655592318)) | 36 | WM | 36 | 4Ws | ④ |
| Wang Y. H. 2014 (85) | China | Median 43.5 | 100 | Criterion F | Herb (Xinyuan-capsule, 心元胶囊) + WM | 38 | WM | 38 | 1M | ④ |
| L. P. Wang 2014 (86) | China | TG 34.5 (2.5)  CG 35.5 (2.5) | 68 | N/R | Herb () + EA  *The prescription is different depending on the pattern identification of disease | 36 | WM | 36 | 8Ws | ②③④ |
| Y. M. Wang 2016 (87) | China | 42.34 (2.13) | 73 | N/R | Herb (Wenxin-granule, [稳心颗粒](https://blog.naver.com/hdjakg/222581067263)) + WM | 45 | WM | 45 | 8Ws | ④ |
| X. Q. Wang 2016 (88) | China | TG 36.2 (3.7) CG 36.6 (2.5) | 66 | CIM | NA | 60 | WM | 60 | 8Ws | ④ |
| Y. D. Wang 2016 (89) | China | TG 35.12 (2.10)  CG 34.65 (2.23) | 66 | N/R | EA + WM | 40 | WM | 40 | 3Ms | ②③ |
| L. J. Wang 2019 (90) | China | TG 43.1 (12.4)  CG 43.8 (12.5) | 60 | N/R | Herb (Shensongyangxin-capsule, [参松养心胶囊](https://blog.naver.com/hdjakg/222655592318)) + WM | 34 | WM | 34 | 4Ws | ④⑤ |
| M. Wang 2019 (91) | China | 47.4 (N/R) | 100 | Criterion G | Herb (Wenxin-granule, [稳心颗粒](https://blog.naver.com/hdjakg/222581067263)) + WM | 45 | WM | 45 | 4Ws | ④ |
| Wang 2020 (92) | China | 38.6 (N/R) | 47 | GCNT, PIM-7 | Herb (Seunghamtang-gagam, 升陷湯加減) | 33 | WM | 33 | 4Ws | ①③④ |
| Wang et al., 2009 (93) | China | TG 33.2 (N/R)  CG 33.6 (N/R) | 89 | N/R | Herb (Hwadamhwalhyeolbang-gagam, 化痰活血方加減) | 80 | WM | 40 | 30Ds | ④ |
| Wang et al., 2014 (94) | China | 45.9 (N/R) | 70 | PIM, DCTS | Herb (Gamigyejiyonggolmoryeo-tang, 加味桂枝龍骨牡蠣湯) | 48 | WM | 46 | 8Ws | ①②③ |
| Wang et al., 2015 (95) | China | N/R | 46 | N/R | Herb (Xinkeshu-tablet, 心可舒) + WM | 40 | WM | 37 | 3Ms | ④ |
| Wang et al., 2017 (96) | China | TG 44.28 (11.34)  CG 43.23 (11.23) | 63 | N/R | Herb (Shensongyangxin-capsule, [参松养心胶囊](https://blog.naver.com/hdjakg/222655592318)) + WM | 32 | WM | 32 | 1M | ④⑤ |
| Wei 2008 (97) | China | 43.6 (N/R) | 57 | PIM | Herb (Gamisoyosan-gagam, 加味逍遙散加減) | 79 | WM | 39 | 4Ws | ④ |
| Wei 2009 (98) | China | TG Median 51.3  CG Median 53.6 | 73 | Criterion A | Herb (Shensongyangxin-capsule, [参松养心胶囊](https://blog.naver.com/hdjakg/222655592318)) | 32 | WM | 31 | 4Ws | ④ |
| Wei 2019 (99) | China | 44.1 (N/R) | 68 | PIM, GCNT, DCTS, DT | Herb (Sihogayonggolmoryeotang-gagam, 柴胡加龍骨牡蠣湯加減) | 30 | WM | 30 | 4Ws | ②③ |
| Wei et al., 2006 (100) | China | Range 21~58 | 73 | PIM-11 | Herb (Wolgukhwan-gagam, 越鞠丸加減) | 24 | WM | 24 | 60Ds | ④ |
| Wu et al., 2014 (101) | China | TG 36.4 (7.9)  CG 37.6 (7.8) | 56 | PIM | NA + WM | 116 | WM | 116 | 4Ws | ①④ |
| Wu et al., 2017 (102) | China | TG 45.78 (8.32)  CG 43.78 (7.32) | 57 | PIM | NA | 30 | WM | 30 | 8Ws | ②③④ |
|  |  | TG 47.28 (8.29)  CG 43.78 (7.32) | 57 | PIM | NA + WM | 30 | WM | 30 | 8Ws | ②③④ |
| Xiao 2014 (103) | China | 51.8 (N/R) | 54 | PIM-3 | Herb (Wenxin-granule, [稳心颗粒](https://blog.naver.com/hdjakg/222581067263)) + WM | 50 | WM | 46 | 4Ws | ④ |
| Xu 2014 (104) | China | 42.95 (N/R) | 45 | N/R | Herb (Shensongyangxin-capsule, [参松养心胶囊](https://blog.naver.com/hdjakg/222655592318)) + WM + Psy | 30 | WM + Psy | 30 | 30Ds | ④⑤ |
| Xu 2016 (105) | China | TG 45.21 (2.78)  CG 45.56 (2.35) | 56 | N/R | Herb (Shensongyangxin-capsule, [参松养心胶囊](https://blog.naver.com/hdjakg/222655592318)) | 150 | WM | 140 | 1M | ②④⑤ |
| Xu et al., 1996 (106) | China | Range 28 ~ 56 | 91 | CCIC | NA | 88 | WM | 88 | 10Ds | ④ |
| Xue et al., 2013 (107) | China | 35.1 (N/R) | 76 | N/R | Herb (Wenxin-granule, [稳心颗粒](https://blog.naver.com/hdjakg/222581067263)) + WM | 25 | WM | 25 | 4Ws | ③④ |
| Xun 2017 (108) | China | 50.5 (N/R) | 69 | PIM-13 | Herb (Gammaekdaejotang-gagam, 甘麥大棗湯加減) + WM | 26 | WM | 25 | 8Ws | ④⑤ |
| Xun et al., 2022 (109) | China | 38.26 (N/R) | 63 | PIM-8, Chinese guidelines for the diagnosis and treatment of heart failure 2014, GCNT | Herb (Gammaekdaejotang-gagam, 甘麥大棗湯加減) + WM | 36 | WM | 36 | 8Ws | ④⑤ |
| Yan 2014 (110) | China | 37.5 (N/R) | 63 | Diagnosis and Cure Standards for Clinical Diseases | Herb (Wenxin-granule, [稳心颗粒](https://blog.naver.com/hdjakg/222581067263)) | 50 | WM | 50 | 15Ds | ④⑤ |
| Yan et al., 2017 (111) | China | TG 38.17 (10.25)  CG 37.52 (9.67) | 86 | PIM, Chinese Medicine | Herb (Guibitang-gagam, 歸脾湯加減) + WM | 46 | WM | 46 | N/R | ④ |
| Yang 2016 (112) | China | 37.10 (2.11) | 79 | Criterion A | Herb (Shensongyangxin-capsule, [参松养心胶囊](https://blog.naver.com/hdjakg/222655592318)) + WM + Psy | 30 | WM + Psy | 18 | 6Ws | ④ |
| Yang 2017 (113) | China | TG 44.8 (8.81)  CG 45.0 (7.15) | 83 | PIM-14, DCTS, Practical Internal Medicine of TCM | EA | 20 | WM | 20 | 4Ws | ①②③④ |
|  |  | TG 45.65 (10.55)  CG 45.0 (7.15) | 83 | PIM-14, DCTS, Practical Internal Medicine of TCM | NA | 20 | WM | 20 | 4Ws | ①②③④ |
| Yang et al., 2017 (114) | China | TG 35.0 (6.1)  CG 36.0 (5.1) | 62 | PIM, DCTS | Herb (Sihogayonggolmoryeo-tang, 柴胡加龍骨牡蠣湯) | 30 | WM | 30 | 21Ds | ④ |
| Yang et al., 2020a (115) | China | TG 52.17 (7.33)  CG 52.31 (7.31) | 88 | PIM, DTGCT | Herb (Sosihotang-hap-gwaruhaebaekbanhatang-gagam, 小柴胡湯+ 瓜蔞薤白半夏湯加減) + WM | 48 | WM | 46 | 4Ws | ②③④ |
| Yang et al., 2020b (116) | China | TG 49.2 (6/7)  CG 49.1 (6.4) | 86 | PIM-14 | Herb (Sosihotang-hap-tonggyuhwalhyeoltang-gagam, 小柴胡湯+通竅活血湯加減) + WM | 42 | WM | 41 | 4Ws | ②③④ |
| Yu 2003 (117) | China | 35.9 (N/R) | 70 | N/R | Herb (Sanjointang-hap-geumnyeongjasan-gagam, 酸棗仁湯+金鈴子散加減) | 65 | WM | 40 | 15Ds | ④ |
| Yu 2015 (118) | China | Range 35~69 | 60 | PIM | Herb (Seosimansintang-gagam, 舒心安神汤加減) | 36 | WM | 36 | 42Ds | ③④ |
| Yuan et al., 2016 (119) | China | 65.37 (2.31) | 45 | N/R | Herb (N/R) | 42 | WM | 42 | 4Ws | ④ |
| Zang 2007 (120) | China | TG 36 (N/R)  CG 34 (N/R) | 68 | Criterion E | Herb (self-made prescription) + NA + WM | 30 | WM | 42 | 2Ms | ④ |
| Zeng 2016 (121) | China | TG 38.0 (5.0)  CG 38.5 (5.5) | 53 | N/R | Herb (Wenxin-granule, [稳心颗粒](https://blog.naver.com/hdjakg/222581067263)) + WM + Psy | 46 | WM + Psy | 46 | 4Ws | ④ |
| Zeng et al., 2010 (122) | China | TG 35 (N/R)  CG 36 (N/R) | 61 | PIM | Herb (Soganyeongsimtang-gagam, 疏肝宁心汤加減) | 40 | WM | 40 | 20Ds | ④ |
| Zhang 2012 (123) | China | Range 28~40 | 100 | Criterion C | Herb (Sihosogansan-hap-yangsimtang, 柴胡消奸散+養心湯) | 56 | WM | 56 | 4Ws | ④ |
| Zhang 2013 (124) | China | 45 (N/R) | 68 | PIM | Herb (Yeongsim-tang, 宁心汤) | 26 | WM | 24 | 6Ws | ④ |
| W. Z. Zhang 2014 (125) | China | TG 40.8 (15.6)  CG 41.2 (16.3) | 47 | N/R | Herb (Mokdanyangsim-bang, 牡丹养心方) + WM + Psy | 144 | WM + Psy | 118 | 2Ms | ④ |
| Y. H. Zhang 2014 (126) | China | TG 49.44 (13.95)  CG 49.31 (14.64) | 58 | PIM-14, DCTS | NA | 30 | WM | 31 | 32Ds | ①③④ |
| Zhang C. S. 2015 (127) | China | 47.5 (11.9) | 60 | N/R | Herb (Haeulyeongsimtang-gagam, 解郁宁心汤加減) | 40 | WM | 40 | 1M | ④ |
| Zhang Y. 2015 (128) | China | TG 35.4 (N/R)  CG 34.8 (N/R) | 38 | N/R | Herb (self-made prescription, Cheonwangbosim-dan and Jaoga-tablet, self-made prescription +天王補心丹+刺五加片) | 40 | WM | 40 | 4Ws | ④ |
|  |  | TG 34.5 (N/R)  CG 34.8 (N/R) | 30 | N/R | Herb (self-made prescription) | 40 | WM | 40 | 4Ws | ④ |
| Zhang 2016 (129) | China | TG 48.97 (13.44)  CG 49.15 (14.68) | 62 | PIM-14, DCTS | NA | 61 | WM | 63 | 32Ds | ①③④ |
| Zhang L. L. 2020 (130) | China | 45.61 (N/R) | 76 | PIM-15, CIM-2 | Herb (New green pellet, 新绿色颗粒) | 36 | WM | 36 | 2Ws | ①③④ |
| H. Zhang 2020 (131) | China | TG 38.3 (N/R)  CG 38.6 (N/R) | 45 | PIM-16, DMTH | Herb (Ansinjeongjihwan-hap-gammaekdaejotang, 安神定智丸+甘麥大棗湯) + WM | 38 | WM | 38 | 4Ws | ②③④ |
| Zhang et al., 2008 (132) | China | 36.8 (7.8) | 65 | CCMD-3 | Herb (Wenxin-granule, [稳心颗粒](https://blog.naver.com/hdjakg/222581067263)) + Psy | 80 | WM + Psy | 80 | 8Ws | ②③④ |
|  |  |  |  |  | Herb (Wenxin-granule, [稳心颗粒](https://blog.naver.com/hdjakg/222581067263)) + WM + Psy | 80 | WM + Psy | 80 | 8Ws | ②③④ |
| Zhang et al., 2012 (133) | China | TG 40.8 (11.2)  CG 40.6 (12.1) | 69 | PIM | Herb (Haeulyeongsimtang-gagam, 解郁宁心汤加減) | 51 | WM | 36 | 30Ds | ④ |
| Zhang et al., 2015 (134) | China | 44.8 (N/R) | 59 | PIM, DCTS | Herb () + NA + EA  *The prescription is different depending on the pattern identification of disease | 89 | WM | 68 | 8Ws | ①②③④ |
| Zhang et al., 2016 (135) | China | TG 35.4 (N/R)  CG 34.8 (N/R) | 63 | N/R | Herb (self-made prescription, Cheonwangbosim-dan and Jaoga-tablet, self-made prescription +天王補心丹+刺五加) | 40 | WM | 40 | 4Ws | ④ |
|  |  | TG 34.5 (N/R)  CG 34.8 (N/R) | 70 | N/R | Herb (self-made prescription) | 40 | WM | 40 | 4Ws | ④ |
| Zhang et al., 2018 (136) | China | TG 42.8 (8.7)  CG 43.6 (7.5) | 62 | PIM | NA + WM | 50 | WM | 50 | 32Ds | ①②③④ |
| Zhang et al., 2019 (137) | China | TG 34.22 (5.31)  CG 34.21 (5.11) | 54 | PIM, DCTS | Herb (Sihogayonggolmoryeotang-gagam, 柴胡加龍骨牡蠣湯加減) + WM | 45 | WM | 45 | N/R | ④ |
| Zhang et al., 2022 (138) | China | TG 45.68 (10.68)  CG 44.87 (9.87) | 61 | PIM, DMTH | Herb (Sihogayonggolmoryeotang-gagam, 柴胡加龍骨牡蠣湯加減) + WM | 40 | WM | 40 | 4Ws | ①②③④⑤ |
| Zhao et al., 2011 (139) | China | Median 42 | 83 | PIM | Herb (Seoganyangsimtang-gagam and Shensongyangxin-capsule, 舒肝养心汤加減+[参松养心胶囊](https://blog.naver.com/hdjakg/222655592318)) | 33 | WM | 33 | 4Ws | ④ |
| Zhou et al., 2009 (140) | China | 41.2 (7.9) | 56 | N/R | NA | 30 | WM | 30 | 4Ws | ④⑤ |
|  |  |  |  | N/R | NA + WM | 30 | WM | 30 | 4Ws | ④⑤ |
| Zhou et al., 2014 (141) | China | Range 28~56 | 75 | Handbook of IM | Herb (Yangsim-tang, 養心湯) | 28 | WM | 28 | 14Ds | ④ |
| Zhu et al., 2019 (142) | China | 39.24 (N/R) | 60 | PIM, DT | Herb (Gamiomijatang-gagam, 加味五味子湯加減) | 35 | WM | 35 | 2Ws | ④ |

*IG, treatment group; CG, comparison group; CCIC, Clinical Disease Diagnosis based on Cure and Improvement Criteria (临床疾病诊断依据治愈好转标准); CCMD, Chinese Classification of Mental Disorder (中国精神障碍分类及诊断标准); CIM, Chinese Internal Medicine (中医内科学); DCTS, Diagnostic and Therapeutic Criteria of TCM Syndromes (中医病证诊断疗效标准); DMTH, Diagnostic efficacy standards and medication specifications for TCM heart disease (中医心病诊断疗效标准与用药规范); DT, Diagnostics of TCM (中医诊断学); DTGCT, Diagnosis and Treatment Guidelines for Common Diseases in TCM (中医内科常见病诊疗指南); GCNT, Guiding Principles for Clinical Research of New TCM (中药新药临床研究指导原则); IM, Internal Medicine (内科学); MTC, Modern Traditional Chinese Medicine Cardiology (现代中医心病学); PIM, Practical Internal Medicine (实用内科学); TCTSP, TCM Clinical Diagnosis and Treatment Terminology: Syndrome section (中医临床诊疗术语证候部分); TCM, Traditional Chinese Medicine; WM, Western medicine; EA, electroacupuncture; NA, normal acupuncture; Psy, Psychotherapy; M, Month; W, Week; D, Day; ①, somatization; ②, depression; ③, anxiety; ④, efficacy rate; ⑤ adverse effect rate;*

*Criterion A, Common symptoms include palpitations, foot stuffiness, shortness of breath, dizziness, fatigue, and possible precordial pain.
No specific pathological signs are present, and other organic diseases and mental disorders should be excluded for diagnosis;*

*Criterion B, Main symptoms: Palpitation or heartache, manifested as rapid, strong, or slow heartbeat, or heavy heartbeat, or sudden beating and stopping.
Secondary symptoms: (1) hot flashes and sweating; (2) insomnia; (3) dizziness; (4) sensory disturbance in hands or feet, which may be consciously swollen; (5) muscle or joint pain; (6) breathlessness; (7) Body feels numb or irritated; (8) Tired or weak easily.
If there are more than 2 main symptoms and secondary symptoms, the diagnosis can be made;*

*Criterion C, (1) Symptoms: palpitations, insomnia, chest tightness, and dizziness caused by anxiety, emotional agitation, mental trauma, or overwork.
(2) The blood pressure is normal or slightly increased (when the mood is tense). Cardiac auscultation: rapid heart rate, enhanced heart sounds, may be accompanied by soft systolic murmurs of grade I-II in the precordial area, and occasionally premature systole.
(3) Auxiliary inspection. Electrocardiogram showed sinus tachycardia, some patients had partial T wave inversion or flattening, propranolol test was positive, exercise test was negative, echocardiography: normal;*

*Criterion D, ① It is most common in young and menopausal women.
② There may be clinical manifestations such as palpitation, palpitation, chest tightness, shortness of breath, precordial pain or discomfort, accompanied by insomnia, dreaminess, anxiety, irritability, etc., usually aggravated after overwork, and the condition is prone to recurrent attacks without obvious signs. Occasionally high blood pressure and increased heart rate can be seen.
③The electrocardiogram is mostly normal, and tachycardia is occasionally seen. Cardiac color Doppler ultrasound and angiography all indicate that there is no organic heart disease.*

*Criterion E, Western medicine diagnostic criteria: typical cardiovascular symptoms, such as palpitations, shortness of breath, precordial pain, etc.; systemic neurosis, such as dizziness, insomnia, anxiety, nervousness, sweating, etc.;
After examination of the whole body and cardiovascular system, hyperthyroidism, coronary heart disease, myocarditis and other organic heart diseases were excluded
TCM diagnostic criteria: TCM syndrome belongs to liver depression and Qi stagnation. Symptoms include palpitations, chest tightness, pain in walking or hypochondriac pain, shortness of breath, good breath, emotional depression, irritability, restless mood, insomnia and dreaminess, pale tongue, thin moss, pulse string.*

*Criterion F, Diagnostic criteria: The clinical symptoms of cardiac neurosis are mainly discomfort and pain in the precordial area, most of which are located in the apex of the heart and the left submammary area.
Obvious physical labor triggers; often accompanied by chest tightness and dyspnea, symptoms relieved after a long sigh; fatigue, fatigue, palpitations, dizziness, dreaminess, insomnia, headache, loss of appetite, mood swings and other symptoms
Auxiliary examination: Electrocardiogram, cardiac X-ray examination, and cardiac color ultrasound examination showed no obvious organic lesion; laboratory biochemical examination showed no obvious abnormality.
On physical examination, the heart beats strongly, the first heart sound is hyperactive, and grade Ⅰ-Ⅱ systolic murmurs can be heard in the mitral valve or pulmonary valve area.*

*Criterion G, The main clinical manifestations are arrhythmia, and the main symptoms include heat, sweating, chest tightness, palpitations, shortness of breath, irritability, insomnia, dreaminess, memory loss, and fatigue.
The concentrations of serum follicle-stimulating hormone and luteinizing hormone increased, and were higher than those in the follicular and ovulation phases of healthy people, while the concentrations of estradiol decreased, and were lower than those in the follicular phase of healthy people.
Arrhythmias caused by organic heart disease, history of hypertension, hyperthyroidism, and electrolyte disturbance were excluded.*

[Bibliographic information of included papers]

1. Bai ZD, Liu LH, Dang T. Effect of Shensong Yangxin capsule combined with bisoprolol fumarate on anxiety or depression in patients with atrial fibrillation. Medical Journal of National Defending Forces in Northwest China. (2017) 38:321-323. doi: 10.16021/j.cnki.1007-8622.2017.05.013

2. Bao SJ, Feng J. 32 Cases of Cardiac Neurosis Treated with Modified Danzhi Xiaoyao Powder. Journal of Changchun University of Traditional Chinese Medicine. (2009) 25:862. doi: 10.13463／j.cnki.cczyy.2009.06.031

3. Cao ZD, Wu SB, Qi QQ. Clinical Observation of Yixinshu Capsules in Treating Cardiac Neurosis. Chinese Journal of Integrative Medicine on Cardio-/Cerebrovascular Disease. (2010) 8:783-784. doi: CNKI:SUN:ZYYY.0.2010-07-011

4. Che D. Study on the Effect of Betaloc and Wenxin Granules in Treating Cardiac Neurosis. Cardiovascular Disease Journal of Integrated Traditional Chinese and Western Med. (2017) 5:20. doi: 10.16282/j.cnki.cn11-9336/r.2017.05.015

5. Chen PL. Observation on the efficacy of Kaiyu Shunxin decoction in treating 38 cases of cardiac neurosis. Guiding Journal of Traditional Chinese Medicine and Pharmacy. (2009)15:27-28. doi: 10.13862/j.cnki.cn43-1446/r.2009.12.024

6. Chen CH. Observation on the efficacy of self-prepared Yangxin Shuyu Decoction in the treatment of cardiac neurosis. CHINA FOREIGN MEDICAL TREATMENT. (2011) 30:136-138. doi: 10.16662/j.cnki.1674-0742.2011.27.013

7. Chen XH. Clinical study on the treatment of cardiac neurosis with Ningxin Anshen Decoction. Journal of New Chinese Medicine. (2012) 44:19-20. doi: 10.13457/j.cnki.jncm.2012.04.009

8. Chen HY. The clinical curative effect of the senate pine capsule in the treatment of cardiovascular neurosis. Journal of Clinical Medical Literature. (2014) 1:58-59. doi: 10.16281/j.cnki.jocml.2014.02.014

9. Chen B. Observation of the Curative Effect of Zaoren Ningxin Capsule for Curing Heart Neurosis. World Latest Medicine Information. (2018) 18:208-209. doi: 10.19613/j.cnki.1671-3141.2018.45.107

10. Chen Y, Wang L. Clinical Observation on 80 Cases of Cardiac Neurosis Treated by Mongolian Medicine. Journal of Medicine & Pharmacy of Chinese Minorities. (2015) 21:25-26. doi: 10.16041/j.cnki.cn15-1175.2015.07.016

11. Chen YI, Zhang CY, Chen SJ, Cheng W. Observation on Efficacy of Shengxian Decoction Combined with Guizhifuling Pills in Modified Treatment of Cardiac Neurosis. Evaluation and Analysis of Drug-Use in Hospitals of China. (2018) 18:595-597. doi: 10.14009/j.issn.1672-2124.2018.05.007

12. Chen YY, He XP. Clinical study of Jianxin Pinglv Pills in the treatment of cardiac neurosis. Heilongjiang Journal of Traditional Chinese Medicine. (2020) 49:385-386. doi: CNKI:SUN:HLZY.0.2020-03-295

13. Chu XF. Comparative study on Wenxin granules and propranolol in the treatment of cardiac neurosis. Chinese Journal of Modern Drug Application. (2011) 5:76. doi: 10.14164/j.cnki.cn11-5581/r.2011.15.012

14. Cui P. Observation on Therapeutic Effect of Chaihu Shugan Powder Combined with Deanxit in Treating Cardiac Neurosis. Practical Clinical Journal of Integrated Traditional Chinese and Western Medicin. (2012) 12:17-19. doi: 10.3969/j.issn.1671-4040.2012.01.010

15. Deng SG, Jin Y, Zhou SG, Zhang S, Fan Q. Clinical Effect of Shensong Yangxin Capsules in Treating Cardiovascular. Heilongjiang Medicine Journal. (2017) 30:135-136. doi: 10.14035/j.cnki.hljyy.2017.01.066

16. Du XY, Liu DH. Xiaoyao Pills Combined with Guipi Pills in the Treatment of 54 Cases of Cardiac Neurosis. Shaanxi Journal of Traditional Chinese Medicine. (2007) 11:1529. doi: CNKI:SUN:SXZY.0.2007-11-068

17. Duan HJ, Li N, Li X, He HT, Sun FJ. Observation on the efficacy of Xiaoyao Powder in the treatment of cardiac neurosis. Sichuan Zhong yi = Sichuanzhongyi. (2004) 22:39–40. doi: 10.3969/j.issn.1000-3649.2004.04.023

18. Fang JZ. Therapeutic Effect which Disperse the Depressed Liver-energy and Ant-luxuriate Treat Cardiac Neurosis Enlarge is Analysised. Guangming Journal of Chinese Medicine. (2008) 10:1493-1494. doi: CNKI:SUN:GMZY.0.2008-10-061

19. Fang JZ, Xia YF, Zheng LL. Observation on the effect of Wenxin Granules on estrogen levels in women with cardiac neurosis during menopause. People's Military Surgeon. (2013) 56:927-928. doi: CNKI:SUN:RMJZ.0.2013-08-044

20. Fu R, Liang KY. Treating 50 Cases of Cardiac Neurosis with Xiaoyao Powder Combined with Antidepressants. China's Naturopathy. (2006) 08:13-14. doi: 10.19621/j.cnki.11-3555/r.2006.08.012

21. Gao HM, Liu YG, Wang SP, Guo YL. The Study of Depression Ameliorated Using Tongxinluo Capsule in Patients with Cardiovascular Neurosis. Journal of Bethune Military Medical College. (2005) 04:204-205. doi: 10.16485/j.issn.2095-7858.2005.04.005

22. Gao XJ, Sun SY. Observation on the clinical efficacy of Jiawei Xiaoyao Pills and Hexintong oral liquid in the treatment of cardiac neurosis. China Journal of Basic Medicine In Traditional Chinese Medicine. (2005) 03:229. doi: CNKI:SUN:ZYJC.0.2005-03-027

23. Gao H, Lu SJ. Observation on the efficacy of Yiqi Smart Decoction in the treatment of cardiac neurosis. Journal of Emergency in Traditional Chinese Medicine. (2008) 17:1660-1669. doi: CNKI:SUN:ZYJZ.0.2008-12-008

24. Guo J. Clinical Study on "Jieyu Ningxin Decoction in Treating Cardiovascular Neurosis”. World Latest Medicine Information. (2015) 15:147. doi: CNKI:SUN:WMIA.0.2015-13-124

25. Guo S. Clinical Study of Wenxinyihao in Treating Cardiac Neurosis (Deficiency of Both Qi and Yin with Blood Stasis [master’s thesis]. China: Changchun university of Chinese medicine. (2021).

26. Guo MM, Lin ZH, Guo JH, Li XM, Yu SX. Clinical Observation on the Treatment of Cardiac Neurosis with Banxia Xiaomi Combined with Erzhi Pills. Shanxi Journal of Traditional Chinese Medicine. (2020) 36:31-33. doi: CNKI:SUN:SHIX.0.2020-09-013

27. Han F. Clinical study on Qizhudihuang Pills in the treatment of cardiac neurosis. Cardiovascular Disease Journal of Integrated Traditional Chinese and Western Med. (2018) 6:167. doi:10.16282/j.cnki.cn11-9336/r.2018.35.133

28. Han B, Zhang Y. Dingxin Decoction (定心汤) Combined with Auricular Point Sticking Therapy for Cardiac Neurosis. Jilin Journal of Traditional Chinese Medicine. (2013) 33:1223-1225. doi: 10.13463/j.cnki.jlzyy.2013.12.009

29. Hu NK, Xue YP, Li GE, Wei CC, Cong H. Observation on Therapeutic Effect of Acupuncture at Neiguan Point on 44 Cases of Cardiac Neurosis. [Shandong Journal of Traditional Chinese Medicine](https://oversea-cnki-net-ssl.openlink.khu.ac.kr/kns/Navi?DBCode=CJFD&BaseID=SDZY). (1994) 06:246-247. doi: CNKI:SUN:SDZY.0.1994-06-003

30. Huang BY. Clinical observation of Xiaoyao powder combined with Wenxin granule in the treatment of cardiac neurosis. Asia-Pacific Traditional Medicine. (2011) 5:104-105. doi: CNKI:SUN:YTCT.0.2011-05-060

31. Huang GR, Huang ZN. Clinical observation on 50 cases of cardiac neurosis treated with Baizi Yangxin Pills. Nei Mongol Journal of Traditional Chinese Medicine. (2016) 35:36-37. doi: 10.16040/j.cnki.cn15-1101.2016.08.037

32. Huo RL. Effects of Xiaoyao Powder combined with modified Guipi Decoction on cardiac autonomic nerve function in patients with cardiac neurosis. Hebei Journal of Traditional Chinese Medicine. (2016) 38:565-568. doi: 10.3969/j.issn.1002-2619.2016.04.026

33. Jiang YX. Clinical Efficacy of Shugan Jianpi Decoction（疏肝健脾方）in Treating Cardiac Neurosis of Liver Depression and Spleen Deficiency and Its Influence on Emotional State. Journal of Liaoning University of Traditional Chinese Medicine. (2021) 23:207-210. doi: 10.13194/j.issn.1673-842x.2021.09.043

34. Gao YQ. Study on Efficacy and Safety of Chaihu Anxin Capsule in Cardiac Neurosis [master’s thesis]. China: Hebei Medical University. (2007).

35. Leng YJ. Clinical Observation on the Treatment of Qi and Blood Deficiency and Stupidity Cardiac Neurosis with Yangxin Chai Huan Decoction [master’s thesis]. China: Changchun University of Traditional Chinese Medicine. (2018).

36. Li SN, Ye ZZ, Shan JJ, Chen JG. A Clincal Observation on Kaixin CapsuleⅡfor Cardiac Neurosis. Journal of Emergency in Traditional Chinese Medicine. (2005) 05:402-403+500. doi: CNKI:SUN:ZYJZ.0.2005-05-007

37. Li XL. Baoxinning Capsules Treating 36 Cases of Cardiovascular Neurosis. China's Naturopathy. (2007) 08:23. doi: 10.19621/j.cnki.11-3555/r.2007.08.027

38. Li MS. Observation on the Curative Effect of Shensong Yangxin Capsules in Treating Cardiac Neurosis. Chinese Journal of Coal Industry Medicine. (2011) 14:1031-1032. doi: CNKI:SUN:ZMGY.0.2011-07-050

39. Li ZH. Clinical Observation on the Treatment of Cardiac Neurosis with Yixinshu Capsule. Chinese Journal of Pharmacoepidemiology. (2012) 21:207-208. doi: 10.19960/j.cnki.issn1005-0698.2012.05.002

40. Li GC. Study on the curative effect of Yangyin Dingpalan decoction in treating cardiac neurosis. Cardiovascular Disease Journal of Integrated Traditional Chinese and Western Me. (2016) 4:84. doi: 10.16282/j.cnki.cn11-9336/r.2016.23.067

41. Li GY. Clinical Study of 'Psycho-cardiology' Model in Patients with Liver Depression and Spleen Deficiency of Cardiac Neurosis [master’s thesis]. China: Yunnan College of Traditional Chinese Medicine. (2018).

42. Li SY. Analysis of the effect of using Guizhi to remove peony and Shuqi Oyster Dragon Bone Jiuxi Decoction in the treatment of cardiac neurosis. Contemporary Medical Symposium. (2018) 16:192-193. doi: CNKI:SUN:QYWA.0.2018-21-140

43. Li XB. Effect of Wenxin Granules on the treatment of patients with cardiac neurosystole and cardiac neurosis. Journal of North Pharmacy. (2021) 18:103-104. doi: CNKI:SUN:BFYX.0.2021-01-046

44. Li JC. Shengmai Yangxin decoction in the treatment of heart neurosis with deficiency of heart and spleen. Clinical Journal of Chinese Medicine. (2021) 13:74-75. doi: CNKI:SUN:ZYLY.0.2021-24-027

45. Li SN, Wei DL. Therapeutic Effect of Kaixin Capsule It for Cardiac Neurosis and Its Effect on Life Quality. Journal of New Chinese Medicine. (2007) 07:14-15+8. doi: 10.13457/j.cnki.jncm.2007.07.010

46. Li BH, Jin WD. Clinical Observation of Yixinshu Capsules Combined with Metoprolol Sustained Release Tablets in Treating Cardiac Neurosis. Chinese Journal of Integrative Medicine on Cardio-/Cerebrovascular Disease. (2015) 13:1416-1418. doi: CNKI:SUN:ZYYY.0.2015-12-022

47. Li SJ, Li RL, Huo JR. Curative effect observation of 55 cases of cardiac neurosis treated with Shugan Huoxue decoction combined with western medicine. Hebei Journal of Traditional Chinese Medicine. (2015) 37:1807-1809. doi: 10.3969/j.issn.1002-2619.2015.12.014

48. Chen L. Ning Xin Tang combined with Western Medicine Treatment Intractable Cardiovascular Neurosis Randomized Controlled Study. Journal of Practical Traditional Chinese Internal Medicine. (2015) 29:90-92. doi: 10.13729/j.issn.1671-7813.2015.12.40

49. Liang XM. The effect of Deng Lao Nuanxin Recipe on cardiac neurosis and its effect on cardiac autonomic nervous function. Inner Mongolia Journal of Traditional Chinese Medicine. (2022) 41:32-33. doi: 10.16040/j.cnki.cn15-1101.2022.06.034

50. Liao LL. 48 cases of cardiac neurosis treated with modified Guipi decoction. Journal of New Chinese Medicine. (2008) 40:72-73. doi: 10.13457/j.cnki.jncm.2008.12.043

51. Liao YQ, Ding ML. Clinical Observation on Modified Zhigancao Decoction in Treating Cardiac Neurosis. The Journal of Medical Theory and Practice. (2013) 26:2838-2839. doi: 10.19381/j.issn.1001-7585.2013.21.018

52. Liu SJ, Liu H, Yang M. Evaluation of the efficacy of Buchang Wenxin Granules and Xinshenning Tablets combined in the treatment of cardiac neurosis. Journal of Zhangjiakou Medical Collage. (2004) 01:55. doi:CNKI:SUN:ZJKB.0.2004-01-027

53. Liu FS. Clinical Study on the Treatment of Cardiovascular Neurosis with Jieyu Ningxin Decoction. Knowledge on Prevention and Treatment of Cardiovascular Disease (Academic Edition). (2014) 08:91-93. doi: CNKI:SUN:XUGB.0.2014-04-039

54. Liu CX. Observing Yangxindingjitang in Treating Cardiovacular Neurosis Syndrome of Asthenia Qi and Blood [master’s thesis]. China: Heilongjiang University of Traditional Chinese Medicine. (2016).

55. Liu YP, Sun DI, Li YB, Wang W, Li YX. Curative effect analysis of modified Huangqi Guizhi Longgu Muli decoction in treating climacteric cardiac neurosis. Chinese Journal of Misdiagnostics. (2012) 12:70-71. doi: CNKI:SUN:ZWZX.0.2012-01-061

56. Liu QA, Wang P, Xia Yu. Clinical observation on Wenxin Anshen Decoction for the treatment of cardiac neurosis. Journal of Changchun University of Traditional Chinese Medicine. (2014) 30:465-467. doi: 10.13463/j.cnki.cczyy.2014.03.034

57. Liu SJ, Zhou YL. Ningxin Anshen Fang,Ciwujia Joint Tianwangbuxin Treating Cardiovascular Neurosis Randomized Controlled Study. Journal of Practical Traditional Chinese Internal Medicine. (2015) 29:7-8. doi: 10.13729/j.issn.1671-7813.2015.08.04

58. Long YH. Xiaoyaosan Treatment of Cardiac Neurosis Randomized Controlled Study. Journal of practical traditional Chinese internal medicine. (2013) 27:49-51. doi: CNKI:SUN:SYZY.0.2013-09-026

59. Lu SC. Wenxin Granules in the Treatment of 40 Cases of Cardiac Neurosis. Shaanxi Journal of Traditional Chinese Medicine. (2005) 07:643-644. doi: CNKI:SUN:SXZY.0.2005-07-026

60. Ma YZ, Liu XW. Curative Effect Observation on 32 Cases of Cardiac Neurosis Treated with Modified Jiangjiang Powder. Journal of Shanxi College of Traditional Chinese Medicine. (2009) 10:44-46. doi: CNKI:SUN:SHAN.0.2009-04-033

61. Pan CX. Treatment of 37 cases of cardiac neurosis by Suanzaoren decoction plus Shixiao powder combined with fluoxetine hydrochloride capsules. Traditional Chinese Medicinal Research. (2013) 26:23-24. doi: CNKI:SUN:ZYYJ.0.2013-09-014

62. Pan C. Observation on Therapeutic Effect of "Ningxin Decoction" in Treating Cardiac Neurosis. Journal of Emergency in Traditional Chinese Medicine. (2018) 27:507-510. doi: CNKI:SUN:ZYJZ.0.2018-03-040

63. Pei XN. Observation on the efficacy of Shexiang Baoxin Pills in the treatment of cardiac neurosis. China Health Industry. (2013) 10:194-195. doi: 10.16659/j.cnki.1672-5654.2013.32.022

64. Pei GX. Observation on Therapeutic Effect of Chaihu Shugan Powder in Treating Cardiac Neurosis. Chinese Journal of Trauma and Disability Medicine. (2013) 21:174-175. doi: CNKI:SUN:XCYZ.0.2013-10-135

65. Qian YY, Li WJ. Clinical research on the treatment of liver depression and spleen deficiency type cardiac neurosis with modified Xiaoyao powder. Guiding Journal of Traditional Chinese Medicine and Pharmacy. (2020) 26:56-58. doi: 10.13862/j.cnki.cn43-1446/r.2020.12.015

66. Qin ZX, Liu QZ, Qian J, Yao W, Liu H, Ji J, Zhong L. Clinical effects of Betaloc combined with Wenxin Granules in the treatment of cardiac neurosis. Journal of Dalian Medical University. (2013) 35:268-270. doi: CNKI:SUN:DLYK.0.2013-03-021

67. Ren LY, Ma XM. Acupuncture combined with massage to treat 30 cases of cardiac neurosis. Zhejiang Journal of Traditional Chinese Medicine. (2010) 45:353. doi: CNKI:SUN:ZJZZ.0.2010-05-034

68. Ren H. Treatment of Yin Deficiency Fire with Lilium Soup Clinical Observation of Cardiac Neurosis [master’s thesis]. China: Changchun University of Traditional Chinese Medicine. (2018).

69. Shen JL, Wang SF. Modified Shiwei Wendan Decoction in Treating 52 Cases of Cardiac Neurosis. Traditional Chinese Medicinal Research. (2007) 04:50. doi: CNKI:SUN:ZYYJ.0.2007-04-022

70. Shi PF, Li QL. 45 Cases in Treating Cardiac Nurosis with Xiaoyao Powder and Oryzanol. Modern Traditional Chinese Medicine. (2013) 33:11-13. doi: 10.13424/j.cnki.mtcm.2013.02.039

71. Su ZY. Clinical Study on Acupuncture Combined with Balanced Cupping Therapy in the Treatment of Cardiovascular Neurosis [master’s thesis]. Changchun University of Traditional Chinese Medicine. (2020).

72. Sun JC. Clinical efficacy of Shensong Yangxin Capsule in the treatment of cardiovascular neurosis. China Practical Medicine. (2015) 10:138-139. doi: 10.14163/j.cnki.11-5547/r.2015.19.095

73. Sun GC, Chang JH, Zhang C. Clinical Study on Modified Danzhi Xiaoyao Powder Combined with Betaloc for Cardiac Neurosis with Syndrome of Binding Constraint of Liver Qi. New Chinese Medicine. (2022) 54:53-56. doi: 10.13457/j.cnki.jncm.2022.15.011

74. Sun XL, Cao MY. 20 cases of cardiovascular neurosis treated with modified Shiwei Wendan decoction - with comparison of 19 cases treated with conventional western medicine. Zhejiang Journal of Traditional Chinese Medicine. (2005) 10:425. doi: CNKI:SUN:ZJZZ.0.2005-10-005

75. Sun J, Xu MH, Leng YN. Observation on the clinical efficacy of modified Chaihu Longgu Oyster Decoction in the treatment of cardiac neurosis. Chinese Journal of Integrative Medicine on Cardio-Cerebrovascular Disease. (2020) 18:3255-3257. doi: CNKI:SUN:ZYYY.0.2020-19-029

76. Tan J, Zhou W, Zhou XJ. Effects of Shensong Yangxin Capsule on symptoms and electrocardiogram of patients with cardiac neurosis. Journal of Emergency in Traditional Chinese Medicine. (2010) 19:1590-1591. doi: CNKI:SUN:ZYJZ.0.2010-09-081

77. Tang XX, Liang L, Zhang Y. Clinical Observation on the Treatment of Cardiac Neurosis with Yixinshu Capsule. Chinese Journal of Integrative Medicine on Cardio-/Cerebrovascular Disease. (2015) 13:949-950. doi: CNKI:SUN:ZYYY.0.2015-07-034

78. Wang BC. Treatment of 20 Cases of Cardiac Neurosis with Chinese Proprietary Drugs Based on Syndrome Differentiation. Chinese Medicine Modern Distance Education of China. (2009) 7:27. doi: CNKI:SUN:ZZYY.0.2009-09-027

79. Wang XQ. 51 cases of cardiac neurosis treated with modified Danzhi Xiaoyao Powder. China's Naturopathy. (2007) 08:29-30. doi:10.19621/j.cnki.11-3555/r.2007.08.034

80. Wang WQ. Clinical Observation on 42 Cases of Cardiac Neurosis Treated by Jianling Decoction. China Medical Herald. (2007) 22:85. doi: CNKI:SUN:YYCY.0.2007-22-062

81. Wang L. Clinical observation on 45 cases of cardiac neurosis treated with Shensong Yangxin Capsule. China Foreign Medical Treatment. (2008) 27:58. doi: 10.16662/j.cnki.1674-0742.2008.34.018

82. Wang XK. Clinical study on Xiaoyao Powder in the treatment of cardiac neurosis. Medical Innovation of China. (2011) 8:165-166. doi: CNKI:SUN:ZYCX.0.2011-19-107

83. Wang H. Clinical study on Xiaoyao Pills in the treatment of cardiac neurosis. Guangming Journal of Chinese Medicine. (2012) 27:1767-1768. doi: CNKI:SUN:GMZY.0.2012-09-030

84. Wang J. Observation on the clinical effect of Shensong Yangxin Capsule in the treatment of cardiac neurosis. Chinese Journal of Trauma and Disability Medicine. (2013) 21:215-216. doi: CNKI:SUN:XCYZ.0.2013-09-164

85. Wang YH. Observation on the Effect of Xinyuan Capsules in Auxiliary Treatment of Cardiac Neurosis. Chinese Journal of Clinical Rational Drug Use. (2014) 7:41. doi: 10.15887/j.cnki.13-1389/r.2014.34.031

86. Wang LP. 36 Cases of Cardiac Neurosis Treated by Syndrome Differentiation Combined with Acupuncture and Moxibustion. Shaanxi Journal of Traditional Chinese Medicine. (2014) 35:832-833. doi: CNKI:SUN:SXZY.0.2014-07-040

87. Wang YM. Efficacy observation of Betaloc combined with Wenxin granule in the treatment of cardiac neurosis. Clinical Journal of Chinese Medicine. (2016) 8:43-44. doi: CNKI:SUN:ZYLY.0.2016-27-027

88. Wang XQ. Clinical research on the treatment of cardiac neurosis with traditional Chinese medicine acupuncture. Cardiovascular Disease Journal of Integrated Traditional Chinese and Western Med. (2016) 4:163-166. doi: 10.16282/j.cnki.cn11-9336/r.2016.26.126

89. Wang YD. Clinical observation of acupuncture combined with fluoxetine in the treatment of cardiac neurosis. Chinese Community Doctors. (2016) 32:113-115. doi: 10.3969/j.issn.1007-614x.2016.5.70

90. Wang LJ. Observation on the Effect of Shensong Yangxin Capsules in Treating Cardiovascular Neurosis. Psychological Monthly. (2019) 14:192. doi: 10.19738/j.cnki.psy.2019.17.176

91. Wang M. The effect of BuChang Wenxin Granule in treating climacteric syndrome with cardiac neurosis. Journal of Anhui Health Vocational & Technical College. (2019) 18:125-126

92. Wang YX. Clinical Observation of Shengxian Decoction in Treating Cardiac Neurosis Caused by Qi-Deficiency and Blood Stasis [master’s thesis]. China: Changchun University of Traditional Chinese Medicine. (2020).

93. Wang SF, Shen JL, Li QF, Xu Y, Ma YJ. Clinical observation on 80 cases of cardiac neurosis treated with phlegm-reducing and blood-activating method. Compilation of papers from the third academic conference on thrombosis of the Chinese Society of Traditional Chinese Medicine; 2009 Nov 6; Zhengzhou, Henan, China: Thrombosis Branch of China Association of Traditional Chinese Medicine: China Association of Traditional Chinese Medicine. (2009). p. 143-144.

94. Wang P, Xia Y, Zheng BW. Clinical Observation on the Treatment of Cardiac Neurosis with Modified Guizhi Longgu Oyster Decoction. Chinese Journal of Traditional Medical Science and Technology. (2014) 21:415-417. doi: CNKI:SUN:TJYY.0.2014-04-038

95. Wang LH, Wang H. Curative effect analysis of Xin Ke Shu combined with betaloc for treatment of cardiovascular neurosis of 40 cases. Chinese Community Doctors. (2015) 31:88-91. doi: 10.3969/j.issn.1007-614x.2015.15.55

96. Wang WB, Li CP. Observation on the effect of Shensong Yangxin Capsule in the treatment of cardiovascular neurosis. Henan Medical Research. (2017) 26:4506-4507. doi: CNKI:SUN:HNYX.0.2017-24-048

97. Wei XH. Aclinica l trials of xiaoyaosan in the treatment of cardiac neurosis. Journal of Sichuan of Traditional Chinese Medicine. (2008) 05:58-59. doi: CNKI:SUN:SCZY.0.2008-05-037

98. Wei AH. Observation on the Curative Effect of Shensong Yangxin Capsules in Treating Cardiac Neurosis. Chinese Journal of Clinical Rational Drug Use. (2009) 2:59-60. doi: CNKI:SUN:PLHY.0.2009-19-050

99. Wei MM. Observation on Treatment effect of Chaihu Longgu Muli Decoction on Cardiac Neurosis of Gan stagnation Pi deficiency syndrome [master's theses]. China: College of Traditional Chinese Medicin. (2019).

100. Wei DL, Li SN. Curative Effect Observation on 24 Cases of Cardiac Neurosis Treated by Kaiyu Therapy. Shandong Journal of Traditional Chinese Medicine. (2006) 05:316-317. doi: 10.16295/j.cnki.0257-358x.2006.05.013

101. Wu M, Chen XL, Cai W. Combination of acupuncture and conventional therapy for the treatment of cardiac neurosis:a report of 116 cases. Shanghai Journal of Traditional Chinese Medicine. (2014) 48:47-48. doi: 10.16305/j.1007-1334.2014.09.015

102. Wu F, Duan JQ. Effect of Acupuncture Combined with Drug on Cardiac Neurosis. [Journal of Hunan University of Chinese Medicine](https://oversea-cnki-net-ssl.openlink.khu.ac.kr/kns/Navi?DBCode=CJFD&BaseID=HNZX). (2017) 37:1261-1264. doi: CNKI:SUN:HNZX.0.2017-11-022

103. Xiao J. Curative Effect Observation of Wenxin Granules Combined with Metoprolol in Treating Cardiac Neurosis. Research of Integrated Traditional Chinese and Western Medicine. (2014) 6:146-147. doi: CNKI:SUN:ZXYH.0.2014-03-014

104. Xu TW. Analysis of the clinical efficacy of Shensong Yangxin Capsule in the treatment of cardiovascular neurosis. Asia-Pacific Traditional Medicine. (2014) 10:111-112. doi: CNKI:SUN:YTCT.0.2014-13-058

105. Xu YF. Analysis of Clinical Effects of Shensong Yangxin Capsules in Treating Cardiovascular Neurosis. Journal of China Prescription Drug. (2016) 14:95-96. doi: CNKI:SUN:ZGCF.0.2016-08-074

106. Xu HM, Zhang YQ. Observation on the Curative Effect of Acupuncture and Moxibustion at Neiguan Point on ST-T Change of Cardiac Neurosis. Journal of Clinical Acupuncture and Moxibustion. (1996) 02:27-28. doi: 10.19917/j.cnki.1005-0779.1996.02.016

107. Xue LY, Li ZM. Clinical observation of Wenxin granules with Deanxit in the treatment of cardiac neurosis. China Modern Medicine. (2013) 20:85-86. doi: CNKI:SUN:ZGUD.0.2013-10-048

108. Sun GX. Modified Ganmai Dazao Decoction combined with western medicine in the treatment of 26 cases of cardiac neurosis. Zhejiang Journal of Traditional Chinese Medicine. (2017) 52:455. doi: 10.13633/j.cnki.zjtcm.2017.06.048

109. Sun JJ, Zhao Y, Li MY. Clinical Observation and Efficacy Analysis of Cardiac Neurosis Treated by Ganmaidazao Soup Combined with Flupentixol and Melitracen Tablets. Clinical Research. (2022) 30:100-103

110. Yan LF. Discussion on Buchang Wenxin Granule in Treatment of Cardiac Neurosis. Journal of Liaoning University of Traditional Chinese Medicine. (2014) 16:187-188. doi: 10.13194/j.issn.1673-842x.2014.05.070

111. Yan JY, Li WJ, Wang LH, Wu CS, Chen S. Impacts of Guipi Decoction on Cardiac Autonomic Nerve Function, Inflammatory Factors and Endothelial Function in the Patients of Cardiac Neurosis. World Journal of Integrated Traditional and Western Medicine. (2017) 12:1249-1252. doi: 10.13935/j.cnki.sjzx.170917

112. Yang YP. Clinical Observation on 30 Cases of Cardiovascular Neurosis Treated by Shensong Yangxin Capsule. Chinese Journal of Clinical Rational Drug Use. (2016) 9:81-82. doi: 10.15887/j.cnki.13-1389/r.2016.19.041

113. YP. Clinical observation on electroacupuncture of cervical sympathetic ganglion in the treatment of cervical cardiac neurosis [master’s thesis]. China: Heilongjiang University of Traditional Chinese Medicine. (2017).

114. Yang JY, Gan X, Yang BJ. 60 cases of cardiac neurosis treated with modified Bupleurum plus Longgu Oyster Decoction. Traditional Chinese Medicine Clinical Research. (2017) 09:81-82. doi: CNKI:SUN:ZYLY.0.2017-10-039

115. Yang C, Long S, Yu JQ. Observation on the efficacy of Xiaochaihu Decoction combined with Gualou Xiebai Banxia Decoction in the treatment of cardiac neurosis with liver stagnation and spleen deficiency. Journal of Liaoning University of Traditional Chinese Medicine. (2020) 22:136-139. doi: 10.13194/j.issn.1673-842x.2020.04.034

116. Yang C, Long S, Yu JQ. Clinical Study on Modified Xiaochaihu Decoction Combined with Tongqiao Huoxue Decoction in the Treatment of Cardiac Neurosis with Blood Stasis Syndrome. International Journal of Traditional Chinese Medicine. (2020) 42:640-643. doi: 10.3760/cma.j.cn115398-20190712-00083

117. Yu HY. Suanzaoren Decoction and Jinlingzi Powder Treating 65 Cases of Cardiovascular Neurosis. Journal of Traditional Chinese Medicine and Chinese Materia Medica of Jilin. (2003) 11:12. doi: 10.13463/j.cnki.jlzyy.2003.11.009

118. Yu Y. Curative Effect Observation of Shuxin Anshen Decoction in the Treatment of Patients with Cardiac Neurosis. Asia-Pacific Traditional Medicine. (2015) 11:118-120. doi: 10.11954/ytctyy.201524058

119. Yuan GF, Wang GX. Observation on the Curative Effect of Traditional Chinese Medicine in Treating Cardiovascular Neurosis. Journal of Clinical Medical Literature. (2016) 3:979. doi: 10.16281/j.cnki.jocml.2016.05.140

120. Zhang XX. 30 cases of cardiac neurosis treated with comprehensive therapy. Chinese Journal of Integrative Medicine on Cardio-/Cerebrovascular Disease. (2007) 02:161-162. doi: CNKI:SUN:ZYYY.0.2007-02-034

121. Zeng FH. Effect of Wenxin Granules on improving the prognosis of patients with arrhythmia and cardiac neurosis. Cardiovascular Disease Journal of Integrated Traditional Chinese and Western Med. (2016) 4:22-24. doi: 10.16282/j.cnki.cn11-9336/r.2016.32.014

122. Zeng SE, Zhao YH, Tan CH. Observation on the efficacy of Shugan Ningxin Decoction in the treatment of cardiac neurosis. Chinese Journal of Integrative Medicine on Cardio-/Cerebrovascular Disease. (2010) 08:784-785. doi: CNKI:SUN:ZYYY.0.2010-07-012

123. Zhang XZ. 56 cases of cardiac neurosis treated with Bupleurum Shugan Powder and Yangxin Decoction. Shandong Journal of Traditional Chinese Medicine. (2012) 31:104-105. doi: 10.16295/j.cnki.0257-358x.2012.02.002

124. Zhang LX. Treating 26 Cases of Cardiac Neurosis with Ningxin Decoction. Hebei Journal of Traditional Chinese Medicine. (2013) 35:209-210. doi: CNKI:SUN:HBZY.0.2013-02-025

125. Zhang WZ. Clinical effect observation of Mudanyangxin prescription combined with fluoxetine hydrochloride capsules on treatment of cardiac neurosis. Cardiovascular Disease Journal of Integrated Traditional Chinese and Western Med. (2014) 2:89-90. doi: 10.16282/j.cnki.cn11-9336/r.2014.16.111

126. Zhang YH. Clinical Randomized Controlled Trail of Treating Cardiac Neurosis with Acupuncture [master’s thesis]. China: Chengdu University of Traditional Chinese Medicine. (2014).

127. Zhang CS. Analysis of the efficacy of Jieyu Ningxin Decoction in the treatment of cardiovascular neurosis. Cardiovascular Disease Journal of Integrated Traditional Chinese and Western Med. (2015) 3:25-27. doi: 10.16282/j.cnki.cn11-9336/r.2015.14.015

128. Zhang Y. Observation on the clinical effect of traditional Chinese medicine in the treatment of cardiovascular neurosis. Asia-Pacific Traditional Medicine. (2015) 11:77-78. doi: CNKI:SUN:YTCT.0.2015-05-038

129. Zhang J. Clinical Randomized Controlled Trail of Treating Cardiac Neurosis with Acupuncture [master’s thesis]. China: Chengdu University of Traditional Chinese Medicine. (2016).

130. Zhang LL. Clinical Observation on the Treatment of Cardiac Neurosis (Deficiency of Heart and Timidity Syndrome) [master’s thesis]. China: CHANGCHUN UNIVERSITY OF CHINESE MEDICINE. (2020).

131. Zhang H. Clinical Observation on Anshen Dingzhi Pill Combined with Ganmai Dazao Decoction in the Treatment of Cardiac Neurosis. [Chinese Medicine Modern Distance Education of China](https://oversea-cnki-net-ssl.openlink.khu.ac.kr/kns/Navi?DBCode=CJFD&BaseID=ZZYY). (2020) 18:73-74. doi: CNKI:SUN:ZZYY.0.2020-06-030

132. Zhang W, Pei XL. Clinical Observation of Wenxin Granules in Treating Cardiac Neurosis. [Jilin Medical Journal](https://oversea-cnki-net-ssl.openlink.khu.ac.kr/kns/Navi?DBCode=CJFD&BaseID=JLYX). (2008) 16:1399-1400. doi: CNKI:SUN:JLYX.0.2008-16-062

133. Zhang CX, Liu LC, Zhou XP. Observation on the efficacy of Jieyu Ningxin Decoction in the treatment of cardiovascular neurosis. Journal of Hebei Traditional Chinese Medicine and Pharmacology. (2012) 27:30-31. doi: 10.16370/j.cnki.13-1214/r.2012.04.004

134. Zhang HY, Zhao ZT, Gao JJ. [Clinical Observation and Literature Review of Traditional Chinese Medicine Combined with Acupuncture in the Treatment of Cardiac Neurosis](https://oversea-cnki-net-ssl.openlink.khu.ac.kr/kns/Detail?sfield=fn&QueryID=10&CurRec=1&recid=&FileName=ZYCX201526037&DbName=CJFDLAST2015&DbCode=CJFD&yx=&pr=&URLID=). [Medical Innovation of China](https://oversea-cnki-net-ssl.openlink.khu.ac.kr/kns/Navi?DBCode=CJFD&BaseID=ZYCX). (2015) 12:116-120. doi: CNKI:SUN:ZYCX.0.2015-26-037

135. Zhang SW, Lan Y, Sun YM. Observation on the Curative Effect of Traditional Chinese Medicine in Treating Cardiovascular. Cardiovascular Disease Journal of Integrated Traditional Chinese and Western Med. (2016) 4:152-154. doi: 10.16282/j.cnki.cn11-9336/r.2016.27.120

136. Zhang YG, Li L, Song YZ, Lin FX, Wu ZJ. Clinical Study on Acupuncture at Five Shu Acupoints Combined with Routine Western Medicine for Cardiac Neurosis. Journal of New Chinese Medicine. (2018) 50:208-210. doi: 10.13457/j.cnki.jncm.2018.07.063

137. Zhang LL, Liu CM, Xu Q. Clinical Analysis of Chaihu Combined Longgu Muli Decoction in Treating Cardiac Neurosis. China & Foreign Medical Treatment. (2019) 38:10-12. doi: 10.16662/j.cnki.1674-0742.2019.03.010

138. Zhang T, Zhang H. Clinical Study on Traditional Chinese Medicine Syndrome, Unhealthy Emotions and Safetyof Patients with Cardiac Neurosis Treated by Chaihu plus Longgu Muli Decoction. Journal of Sichuan of Traditional Chinese Medicine. (2022) 40:73-76. doi: CNKI:SUN:SCZY.0.2022-01-022

139. Zhao SF, Zeng SE, Yang DM, Zhao BQ. Clinical study on the treatment of cardiac neurosis with self-prepared Shugan Yangxin Decoction and Shensong Yangxin Capsules. Journal of Emergency in Traditional Chinese Medicine. (2011) 20:690-695.

140. Zhou ZZ, Huo H, Li WM. Discussion on Cardiac Neurosis Treated with Drug and Acupuncture. [Clinical Medical Engineering](https://oversea-cnki-net-ssl.openlink.khu.ac.kr/kns/Navi?DBCode=CJFD&BaseID=YBQJ). (2009) 16:47-48. doi: CNKI:SUN:YBQJ.0.2009-08-023

141. Zhou YB, Gao Y, Sun J. Yangxin Decoction in the Treatment of Cardiac Neurosis: Clinical Observation of 56 Cases. Information on Traditional Chinese Medicine. (2014) 31:132-135. doi: CNKI:SUN:ZYXN.0.2014-04-052

142. Zhu CH, Zhang W. Clinial observation on treating 70 cases of cardiac neurosis of the Qiyin Liangxu type with the Jiawei Wuweizi decoction. Clinical Journal of Chinese Medicine. (2019) 11:85-87. doi: CNKI:SUN:ZYLY.0.2019-26-033

**Supplementary Table 3.** Descriptions of the included acupuncture and related therapies.

| **Article label** | **Style of acupuncture** | **Names of acupuncture points used** | **Number of needles** | **Needling Depth** | **Acupuncture reaction** | **Retention time** | **Frequency and course** |
| --- | --- | --- | --- | --- | --- | --- | --- |
| **Herbal medicine + Acupuncture** | | | | | | | |
| Wang 2014 (116) | EA | Baihui (GV20), Shenting (GV24), Neiguan (PC6), Zusanli (ST36), Taichong (LR3), Sanyinjiao (SP6) | 10 | NR | NR | 20~30min | Once a day, for 8 weeks |
| Zhang et al., 2015 (117) | ACU + EA | ACU: Baihui (GV20), Shenting (GV24)  ACU+EA: Neiguan (PC6), Zusanli (ST36), Taichong (LR3), Sanyinjiao (SP6) | 10 | NR | NR | NR | Once a day, 5 days a week for 8 weeks |
| **Acupuncture Only** | | | | | | | |
| Hu et al., 1994 (132) | ACU | Neiguan (PC6) | 2 | 1 F-cun | Deqi | 10min | Once a day, 10 days |
| Wang 2016 (133) | ACU | Shenting (GV24), Neiguan (PC6), Taichong (LR3), Zusanli (ST36), Xinshu (BL15), Pishu (BL20), Sanyinjiao (SP6) etc | 13 + a | NR | NR | 30min | Once a day, for 8 weeks |
| Wu et al., 2017 (134) | ACU | Main acupoint : Baihui (GV20), Shenting (GV24), Neiguan (PC6), Shenmen (HT7), Xinshu (BL15), Daling (PC7)  Other acupoint :  Headache & dizziness : Fengchi (GB20), temple (EX-HN5), Quchi (LI11)  Insomnia : Sanyinjiao (SP6), Anmian  Dual deficiency of qi and blood : Zusanli (ST36), Pishu (BL20) | 10 + a | NR | Deqi | 30min | Once a day, 6 days a week for 8 weeks |
| Xu et al., 1996 (135) | ACU | Neiguan (PC6) | 2 | 1 F-cun | Deqi | 10min | Once a day, 10 days |
| Yang 2017a (136) | ACU | Main acupoint: C4-7 Cervical Hyeopcheok acupoint  Other acupoint : Xinshu (BL15), Jueyinshu (BL14), Juque (CV14), Danzhong (CV17), Shenmen (HT7), Neiguan (PC6) | 6 + a | Main acupoint  1 F-cun | NR | 30min | Once a day, 5 days a week for 4 weeks |
| Yang 2017b (136) | EA | Main acupoint: Sympathetic ganglion (Upper, Lower)  Other acupoint: Xinshu (BL15), Jueyinshu (BL14), Juque (CV14), Danzhong (CV17), Shenmen (HT7), Neiguan (PC6) | 4 + a | Main acupoint  1 F-cun | NR | 30min | Once a day, 5 days a week for 4 weeks |
| Zhang 2014 (137) | ACU | Main acupoint: Xinshu (BL15), Jueyinshu (BL14), Pishu (BL20), Ganshu (BL18), Shenmen (HT7), Neiguan (PC6), Juque (CV14), Danzhong (CV17)  Other acupoint: Dual deficiency of the heart and spleen: Zusanli (ST36), Tianshu (ST25)  Yin deficiency with effulgent fire : Taixi (KI3), Sanyinjiao (SP6)  Heart yang deficiency: Mingmen (GV4), Guanyuan (CV4), Zusanli (ST36) | 14 + a | NR | Deqi | 30min | Once a day, 30 days |
| Zhang 2016 (138) | ACU | Main acupoint: Xinshu (BL15), Jueyinshu (BL14), Shenmen (HT7), Neiguan (PC6), Ganshu (BL18), Pishu (BL20), Juque (CV14), Danzhong (CV17)  Other acupoint: Dual deficiency of the heart and spleen: Zusanli (ST36), Tianshu (ST25)  Yin deficiency with effulgent fire: Taixi (KI3), Sanyinjiao (SP6)  Heart yang deficiency: Mingmen (GV4), Guanyuan (CV4), Zusanli (ST36) | 14 + a | All depths are different depending on the acupuncture points | Deqi | 30min | Once a day, 30 days |
| Zhou et al., 2009 (139) | ACU | Eight confluent points: Neiguan (PC6), Gongsun (SP4) | 4 | NR | NR | 30min | Once a day, for 4 weeks |
| **Acupuncture + Western medicine** | | | | | | | |
| Wang 2016 (140) | EA | Baihui (GV20), Shenting (GV24), Neiguan (PC6), Zusanli (ST36), Taichong (LR3), Sanyinjiao (SP6) etc | 10 + a | NR | NR | 20-30 min | Once a day, for 3 months |
| Wu et al., 2014 (141) | ACU | Joined puncture: Both Neiguan (PC6) - Jianshi (PC5), Qiuxu (GB40) - Zhaohai (KI6) | 4 | NR | Deqi | 30min | Once a day, for 4 weeks |
| Wu et al., 2017b (134) | ACU | Main acupoint: Baihui (GV20), Shenting (GV24), Neiguan (PC6), Shenmen (HT7), Xinshu (BL15), Daling (PC7)  Other acupoint :  Headache & dizziness: Fengchi (GB20), temple (EX-HN5), Quchi (LI11)  Insomnia: Sanyinjiao (SP6), Anmian  Dual deficiency of qi and blood: Zusanli (ST36), Pishu (BL20) | 10 + a | NR | Deqi | 30min | Once a day, 6 days a week for 8 weeks |
| Zhang et al., 2018 (142) | ACU | Xinshu (BL15), Feishu (BL13), Pishu (BL20), Ganshu (BL18), Shenshu (BL23) | 10 | NR | Deqi | 15min | Once a day, 30 days |
| Zhou et al., 2009b (139) | ACU | Eight confluent points: Neiguan (PC6), Gongsun (SP4) | 4 | NR | NR | 30min | Once a day, for 4 weeks |

## Supplementary Figures


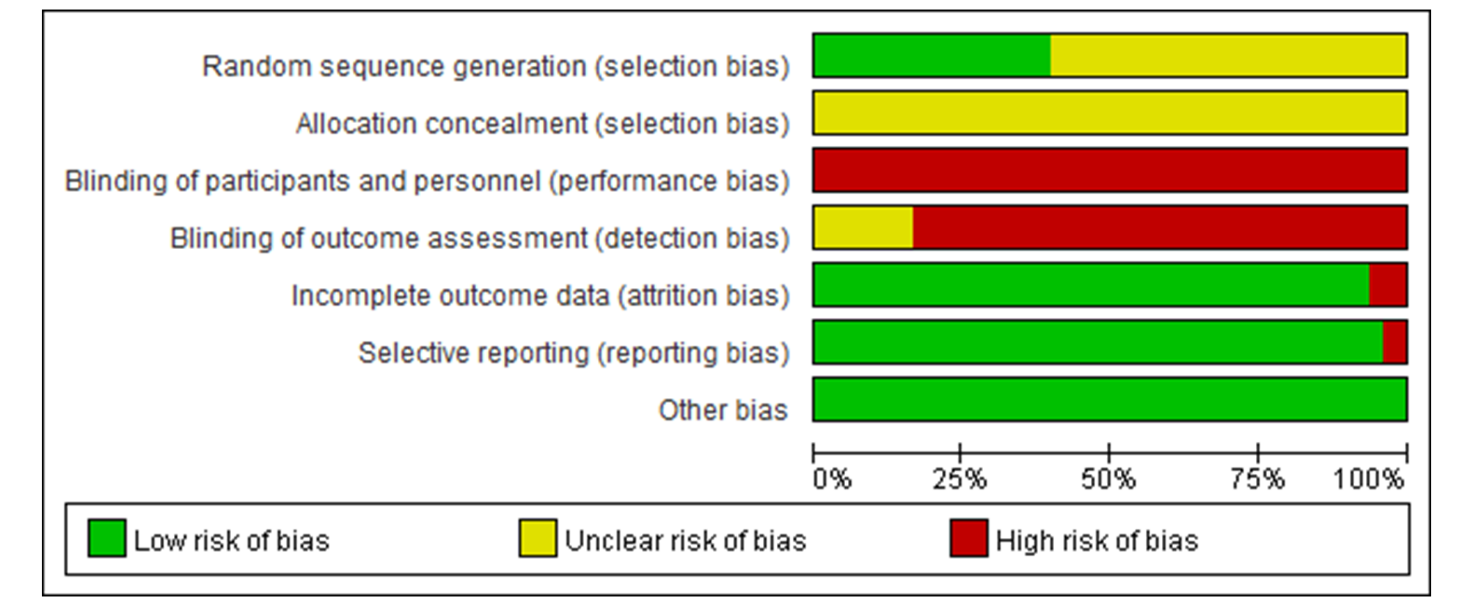


**Supplementary Figure 1.** The risk of bias for 48 studies comparing herbal and Western medicine combined treatment with Western medicine alone.


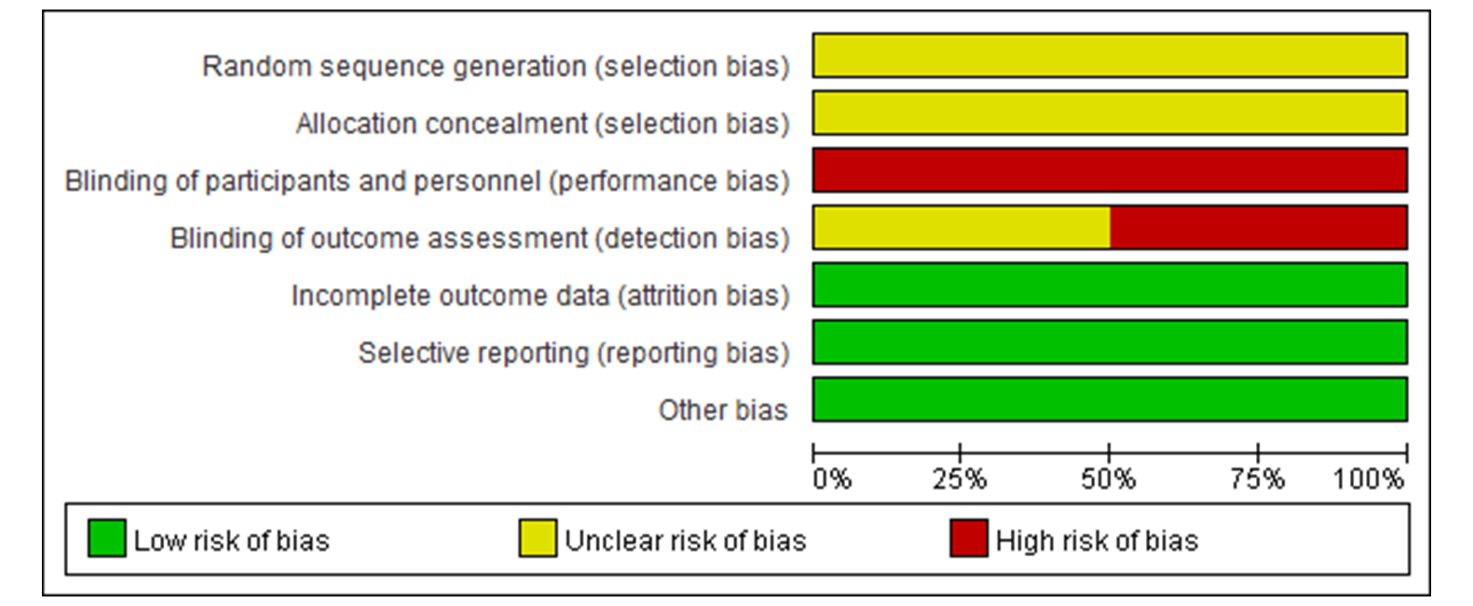


**Supplementary Figure 2.** The risk of bias for 2 studies comparing herbal medicine and acupuncture combined treatment with Western medicine alone.


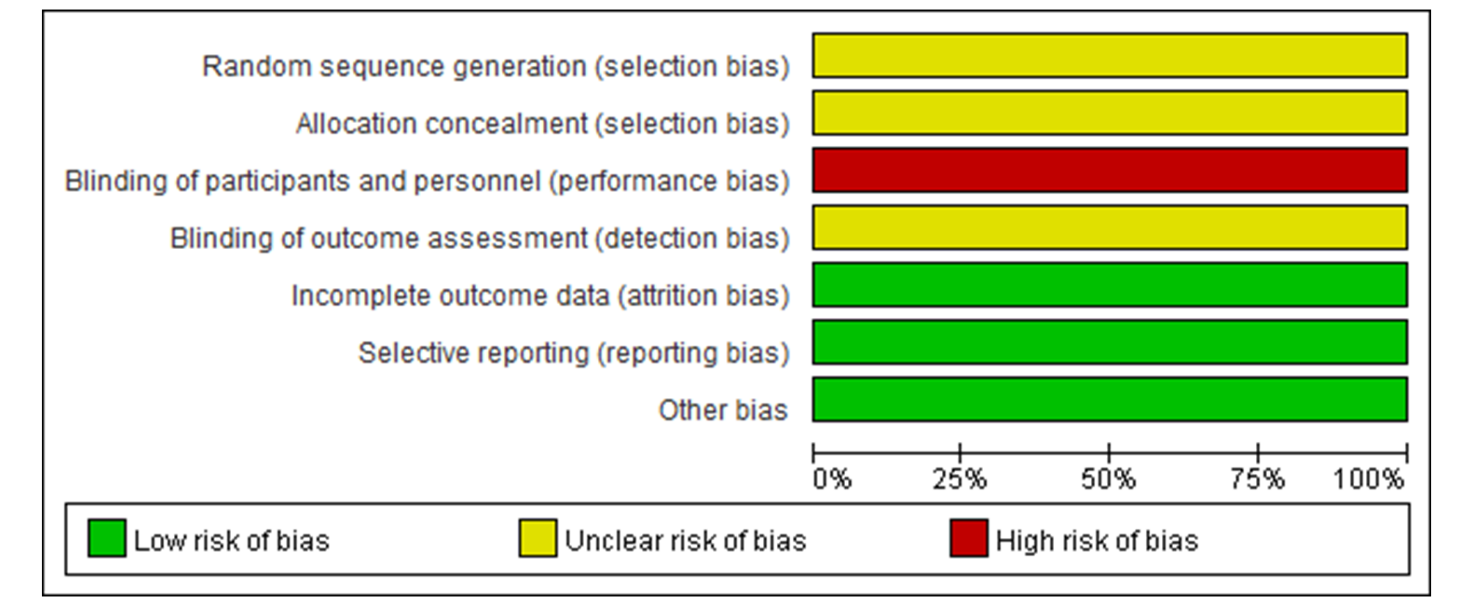


**Supplementary Figure 3.** The risk of bias for 1 study comparing herbal medicine and acupuncture, Western medicine combined treatment with Western medicine alone.


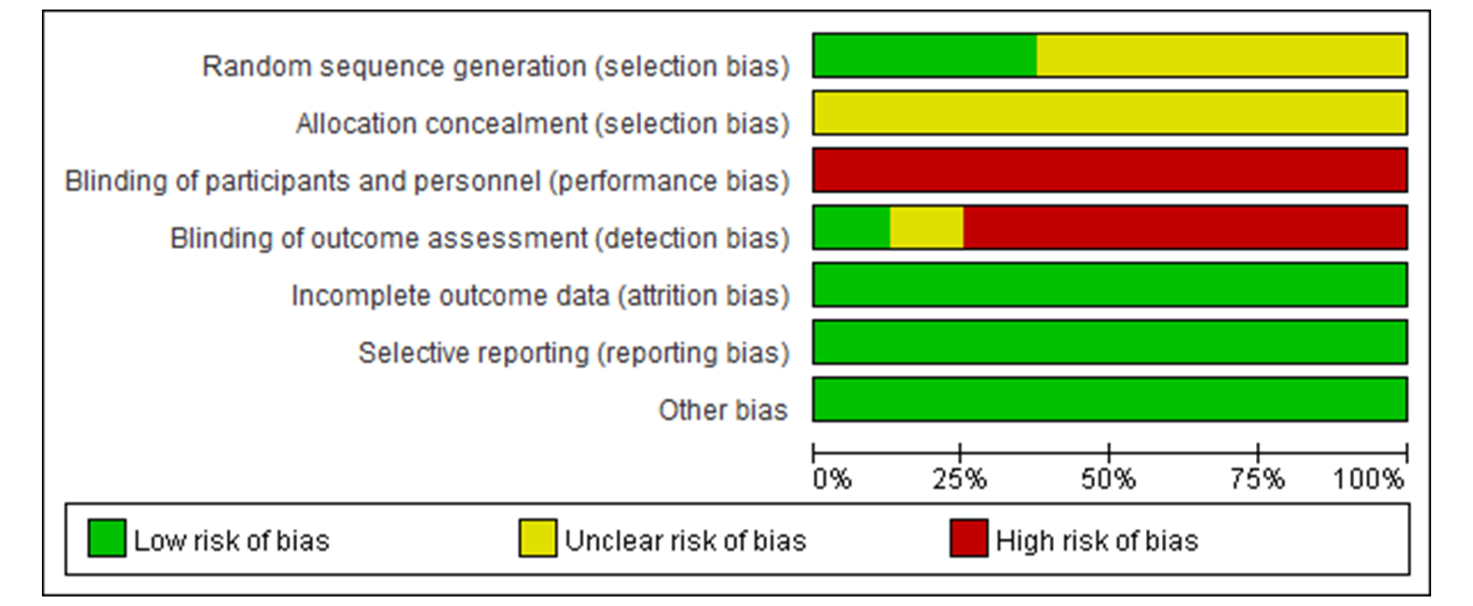


**Supplementary Figure 4.** The risk of bias for 8 studies comparing herbal medicine plus psychotherapy versus Western medicine plus psychotherapy.


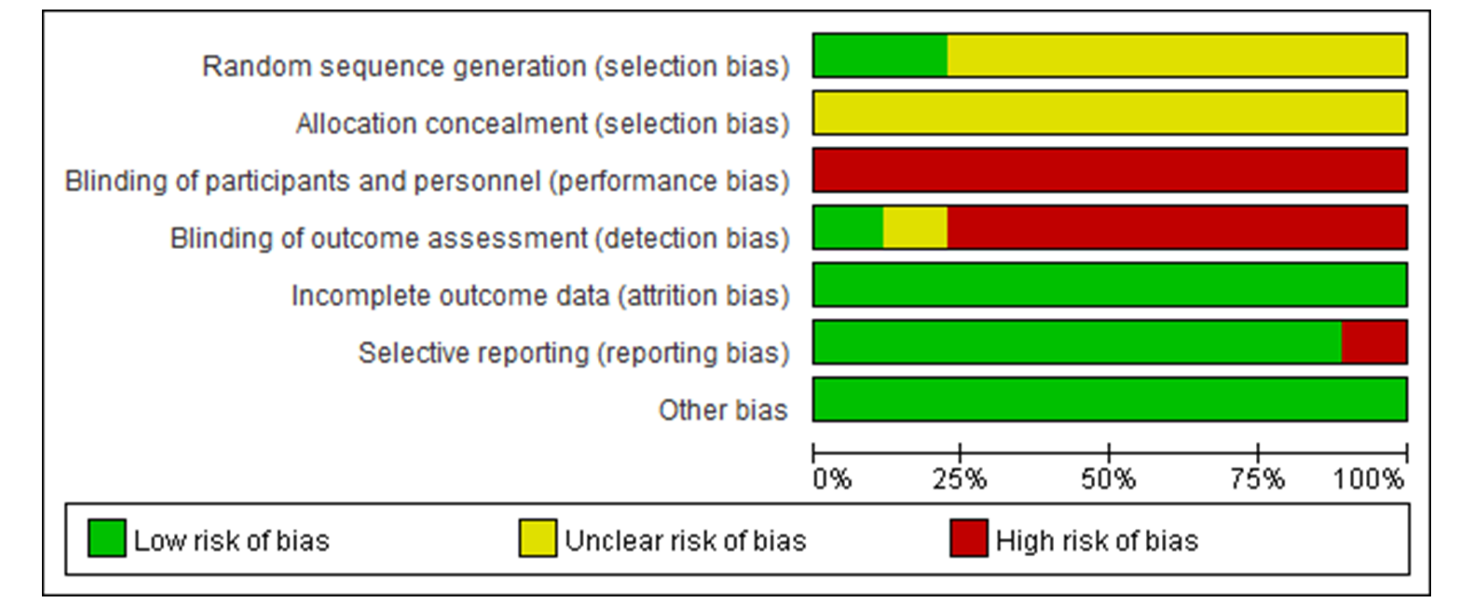


**Supplementary Figure 5.** The risk of bias for 9 studies comparing herbal medicine and psychotherapy, Western medicine combined therapy versus Western medicine and psychotherapy.


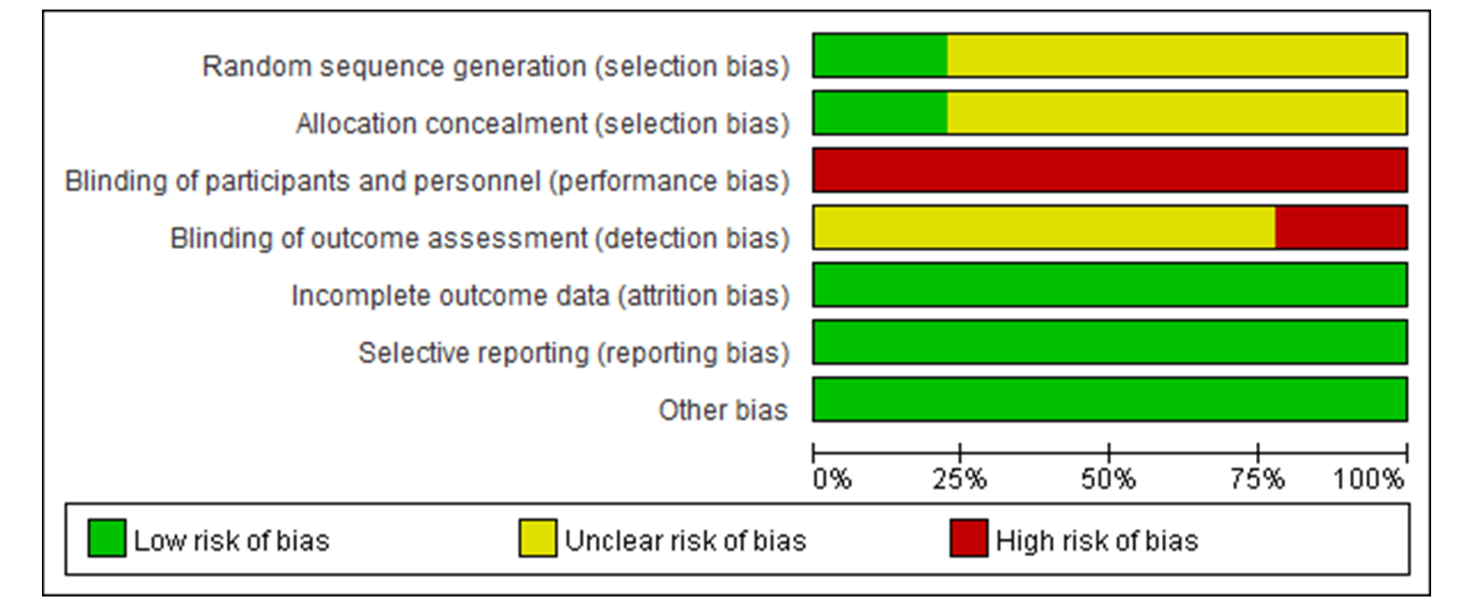


**Supplementary Figure 6.** The risk of bias for 9 studies comparing acupuncture and Western medicine.


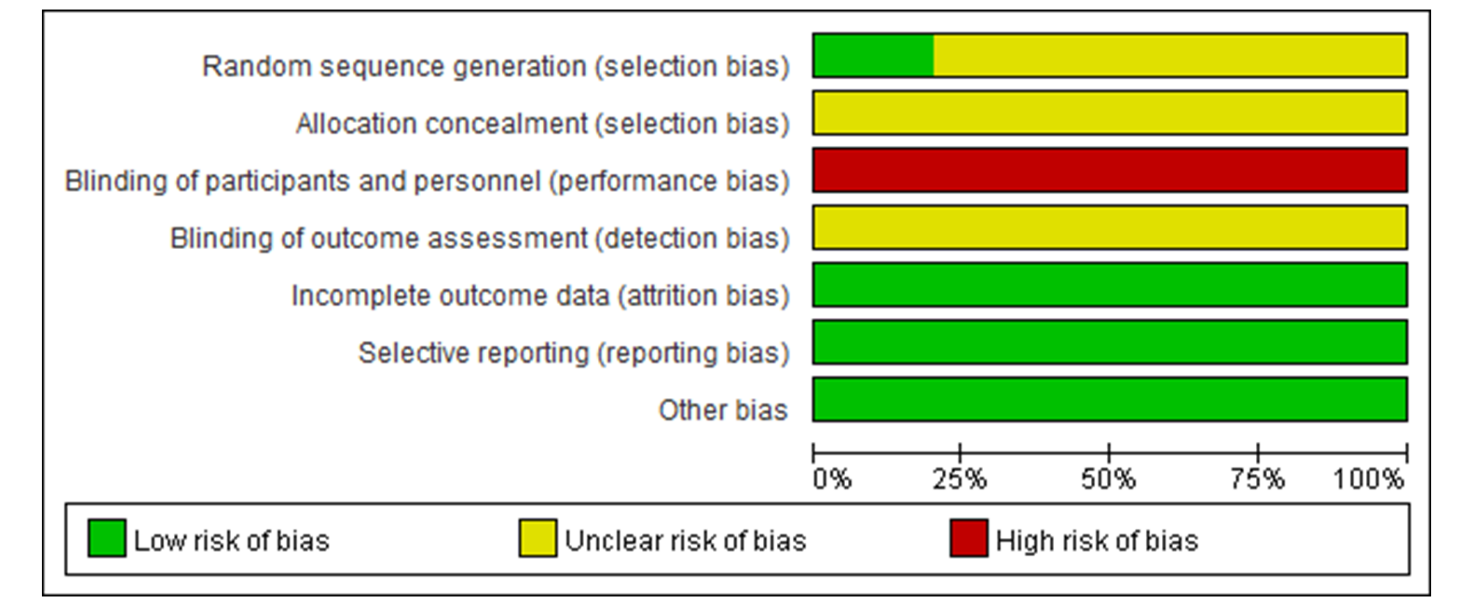


**Supplementary Figure 7.** The risk of bias for 5 studies comparing acupuncture and Western medicine combined treatment with Western medicine alone.


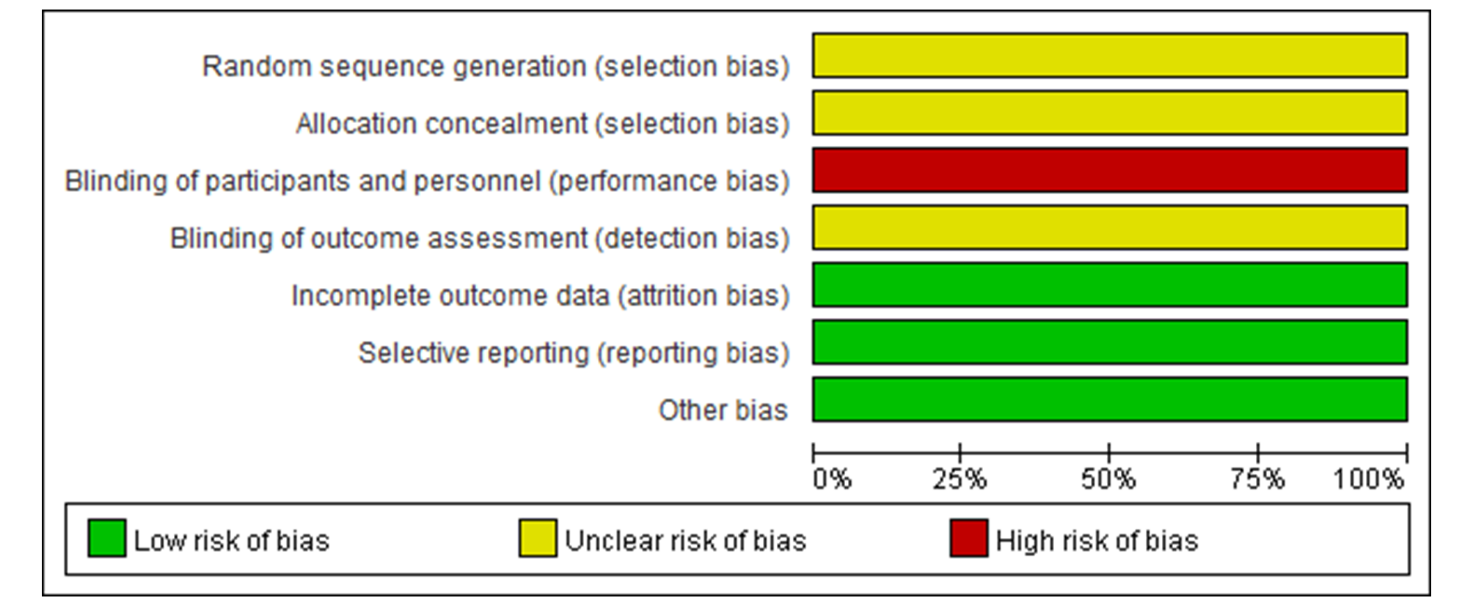


**Supplementary Figure 8.** The risk of bias for 1 study comparing acupuncture and chuna combined treatment with Western medicine alone.

**
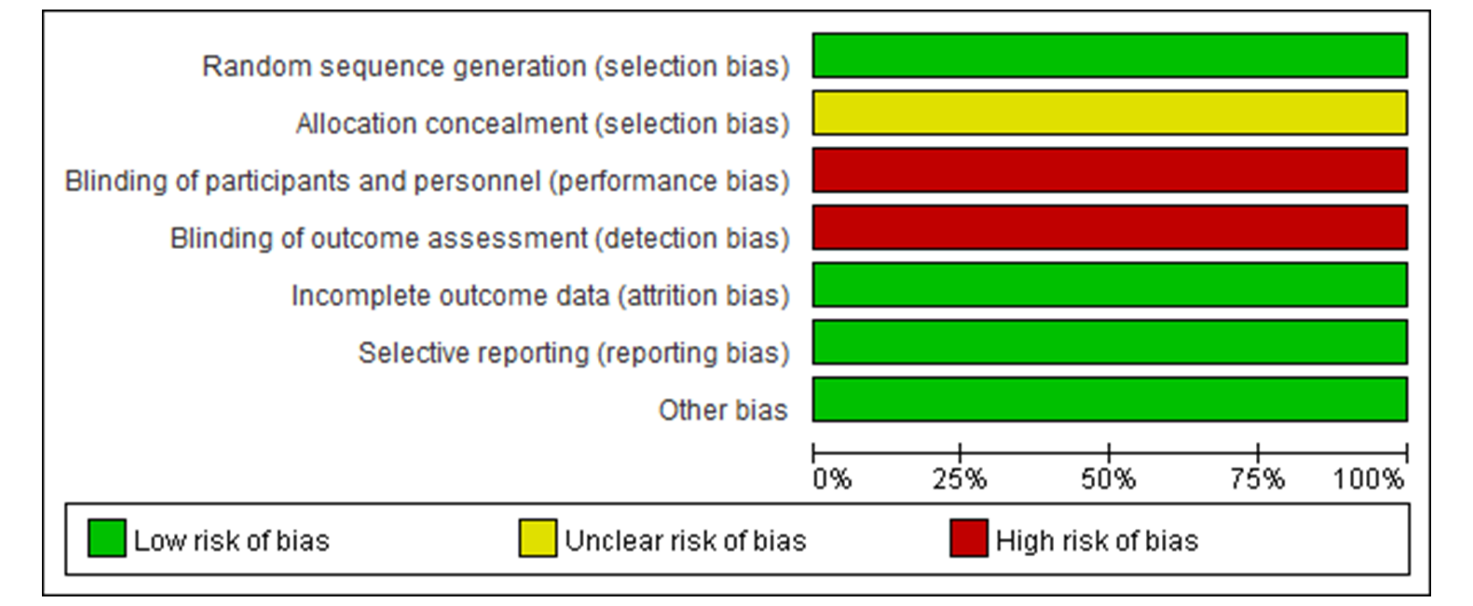
**

**Supplementary Figure 9.** The risk of bias for 1 study comparing acupuncture and cupping combined treatment with Western medicine alone.

**
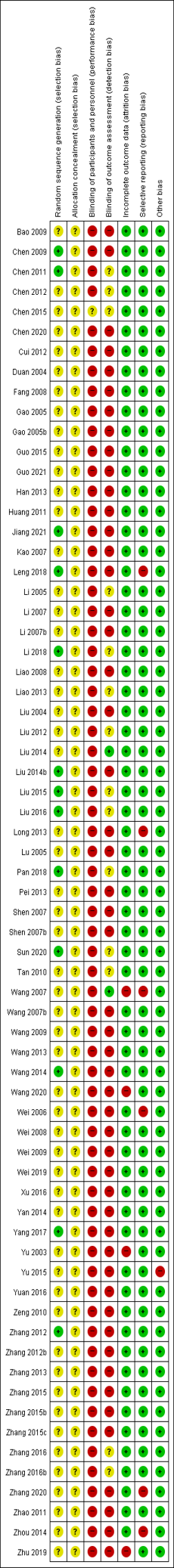
**
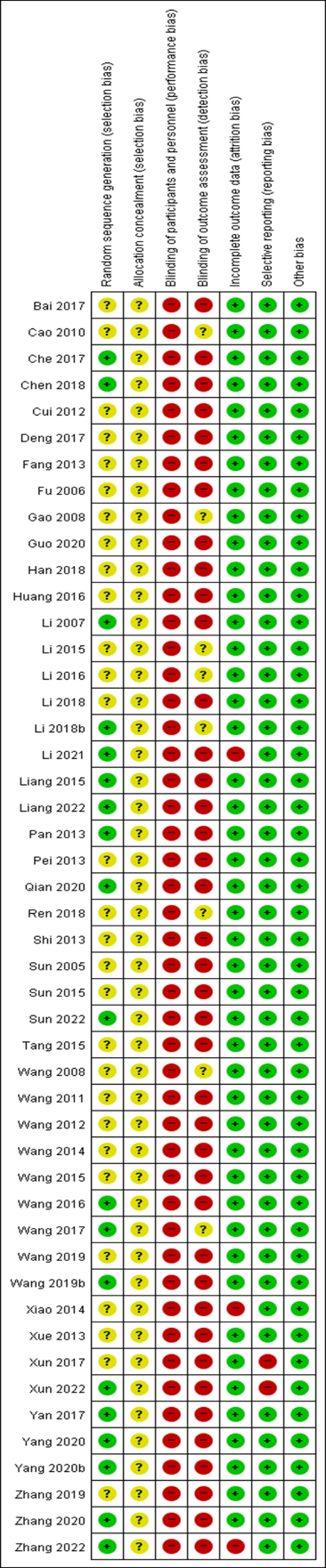

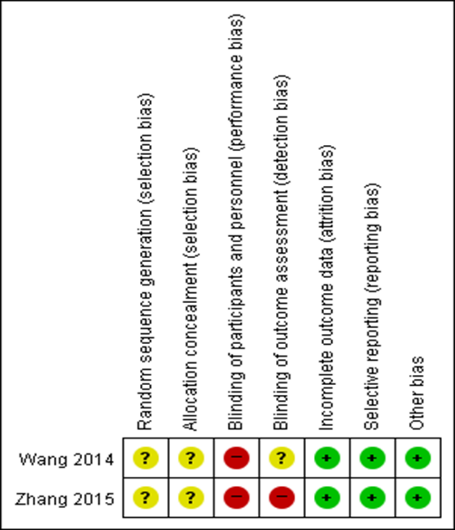
 **
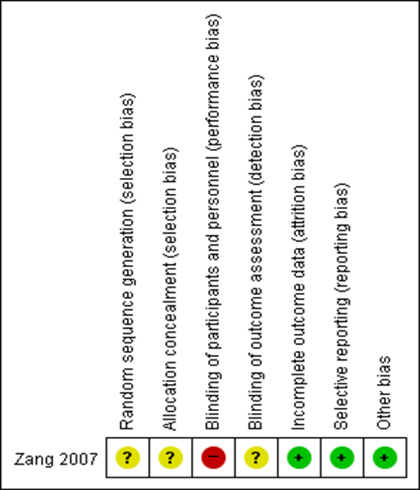
**

**Supplementary Figure 10.** Risk of bias summary of 67 studies comparing herbal medicine and Western medicine.

**Supplementary Figure 11.** Risk of bias summary for 48 studies comparing herbal and Western medicine combined treatment with Western medicine alone.

**Supplementary Figure 12.** Risk of bias summary for 2 studies comparing herbal medicine and acupuncture combined treatment with Western medicine alone.

**Supplementary Figure 13.** Risk of bias summary for 1 study comparing herbal medicine and acupuncture, Western medicine combined treatment with Western medicine alone.

**
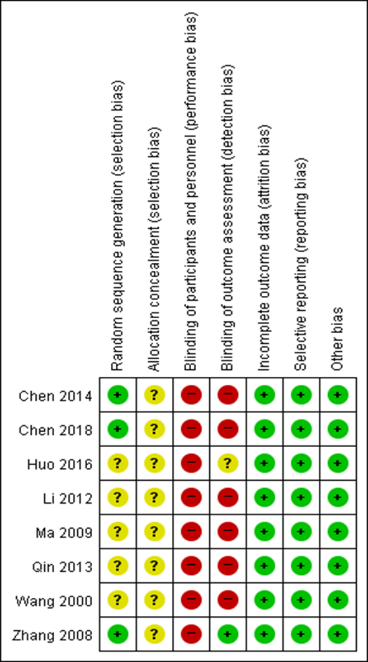
**
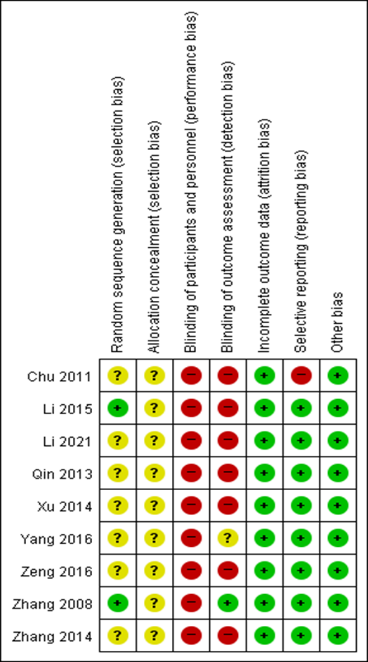

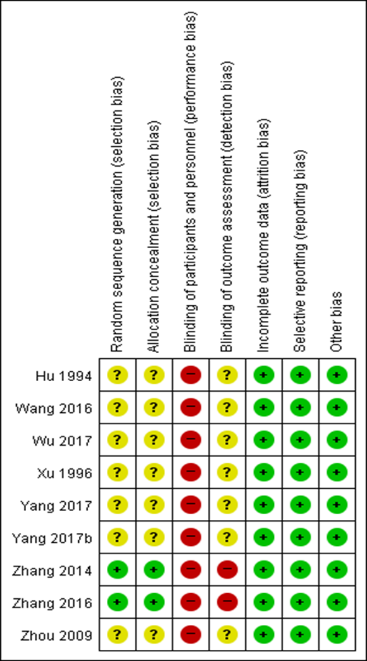

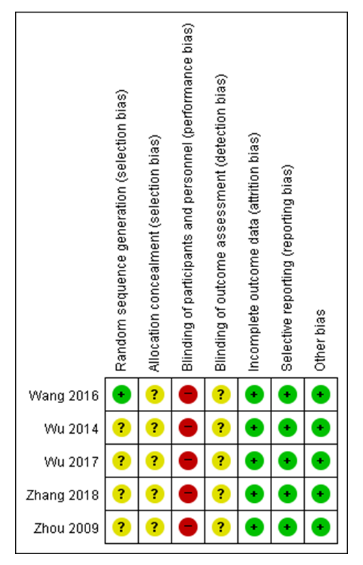


**Supplementary Figure 14.** Risk of bias summary for 8 studies comparing herbal medicine plus psychotherapy versus Western medicine plus psychotherapy.

**Supplementary Figure 15.** Risk of bias summary for 9 studies comparing herbal medicine and psychotherapy, Western medicine combined therapy versus Western medicine and psychotherapy.

**Supplementary Figure 16.** Risk of bias summary for 9 studies comparing acupuncture and Western medicine.

**Supplementary Figure 17.** Risk of bias summary for 5 studies comparing acupuncture and Western medicine combined treatment with Western medicine alone.

**
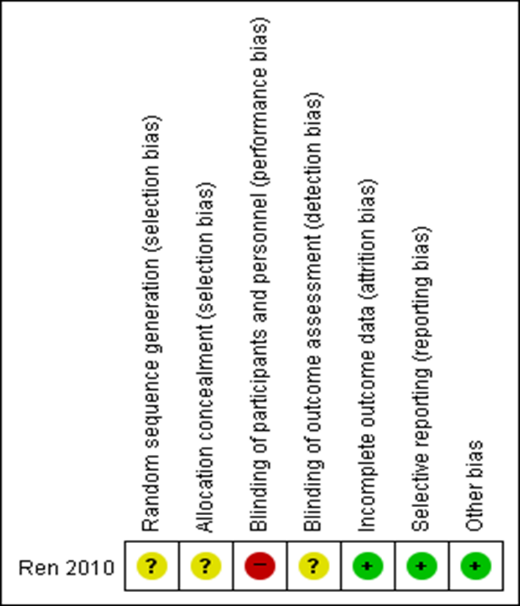

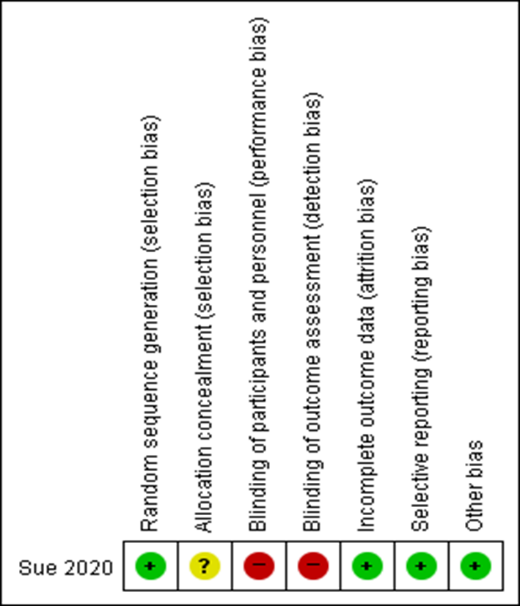
**

**Supplementary Figure 18.** Risk of bias summary for 1 study comparing acupuncture and chuna combined treatment with Western medicine alone.

**Supplementary Figure 19.** Risk of bias summary for 1 study comparing acupuncture and cupping combined treatment with Western medicine alone.


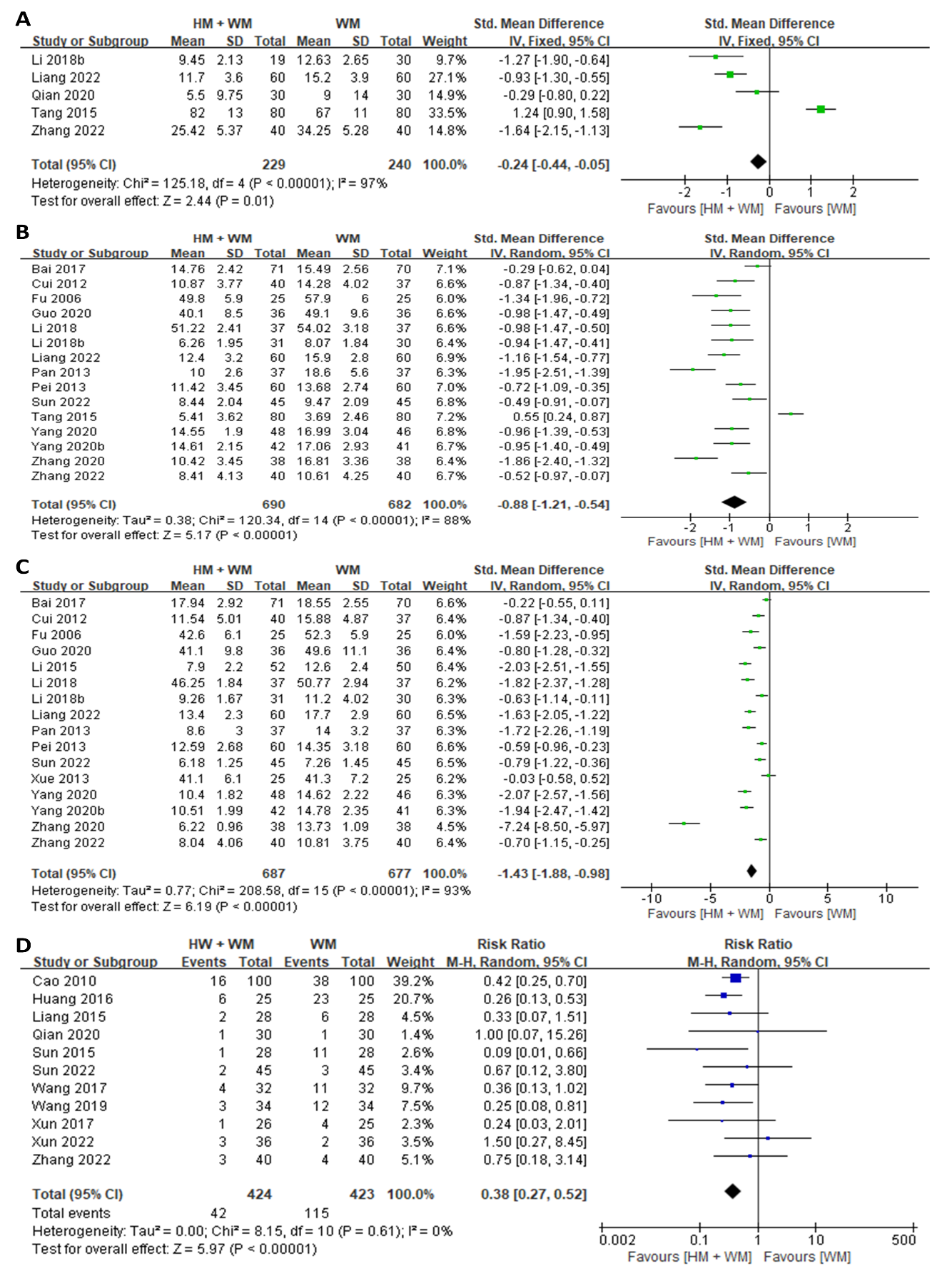


**Supplementary Figure 20.** Forest plot showing the effect of herbal and Western medicine combined treatment versus Western medicine alone. (A) Forest plot for somatization, (B) forest plot for depression, (C) forest plot for anxiety, (D) forest plot for adverse effect rates.


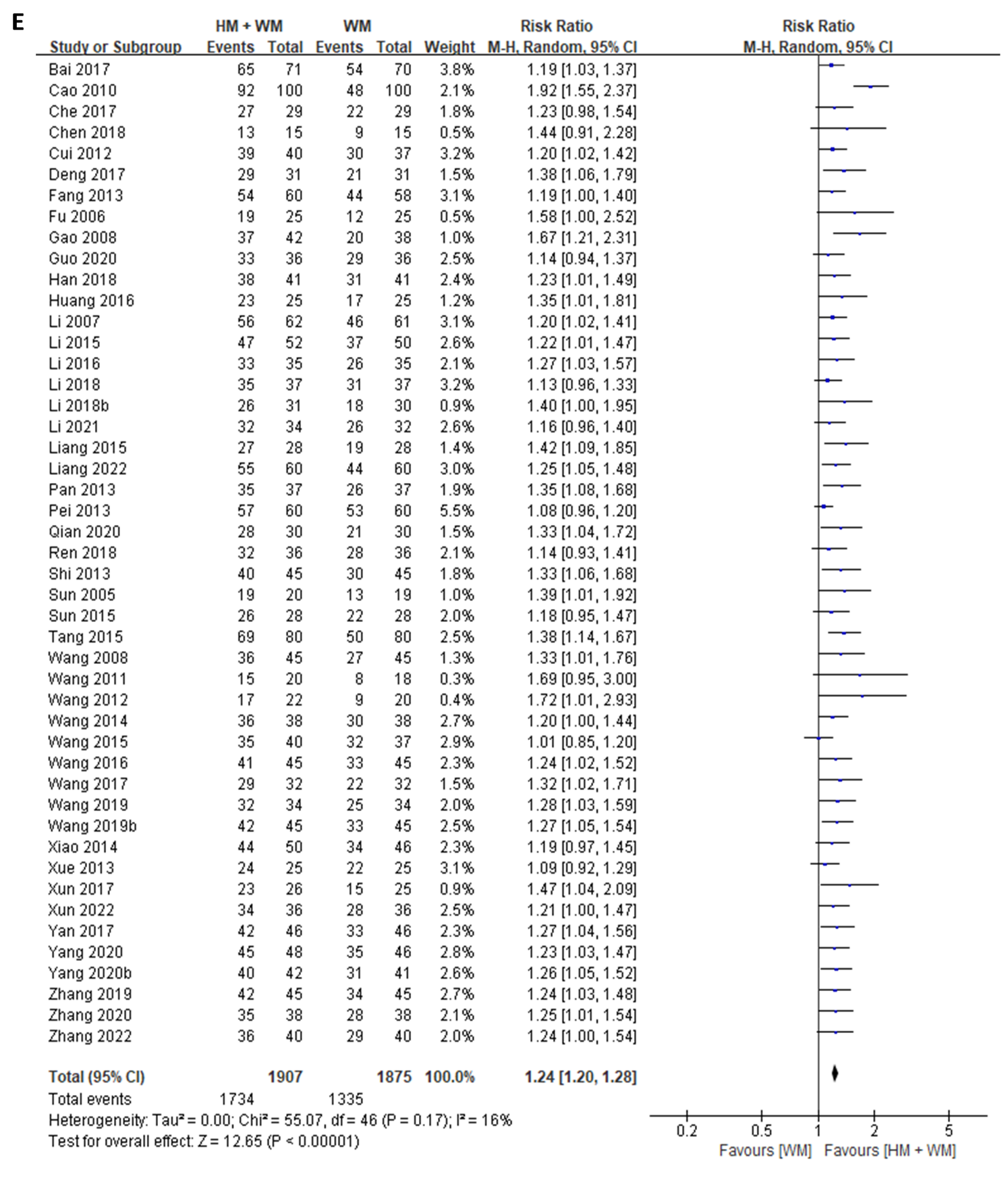


**Supplementary Figure 21.** Forest plot showing the effect of herbal and Western medicine combined treatment versus Western medicine alone. (E) Forest plot for effectiveness rates.


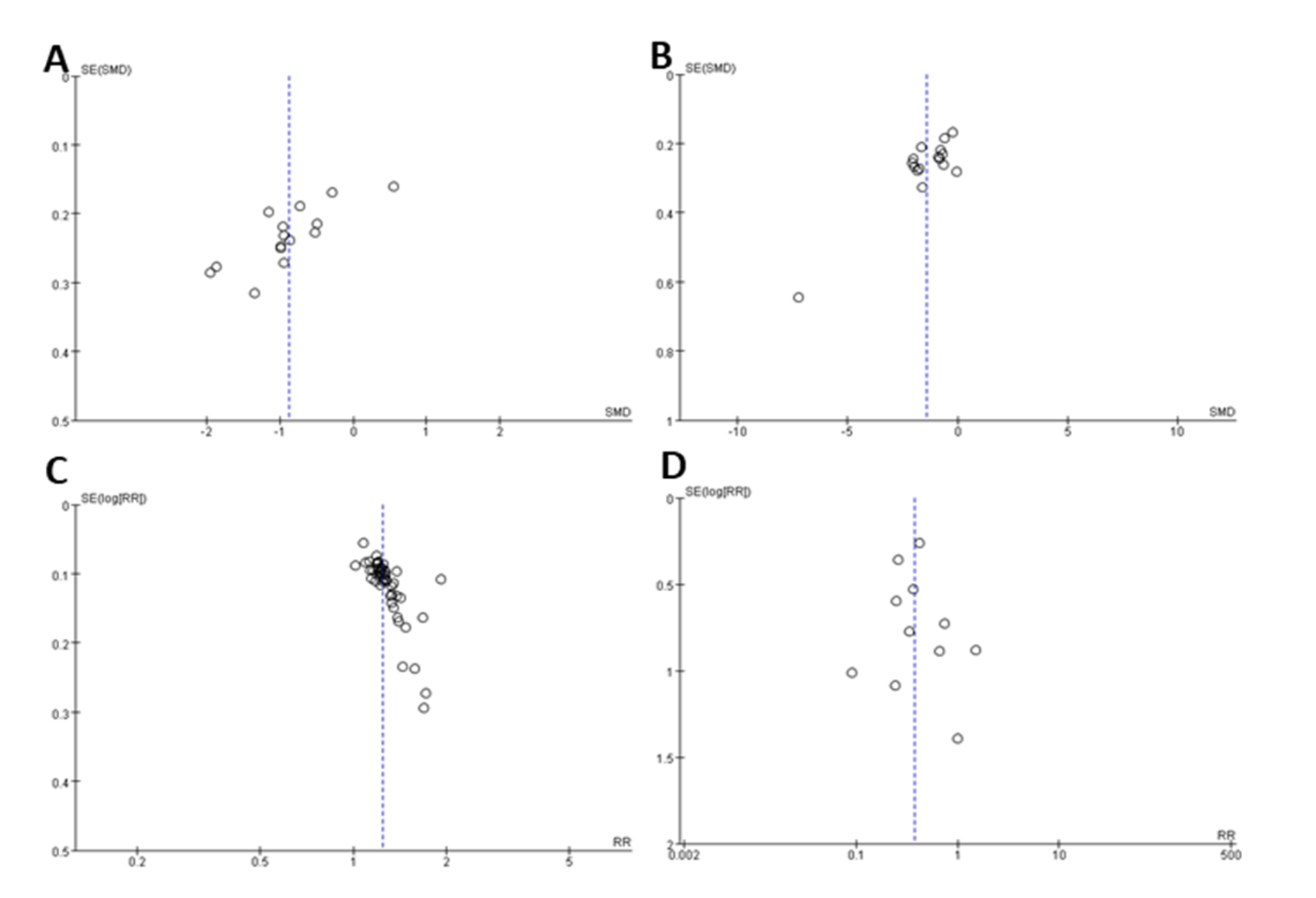


**Supplementary Figure 22.** Funnel plot of the studies comparing herbal and Western medicine combined treatment with Western medicine alone. (A) Funnel plot for depression, (B) funnel plot for anxiety, (C) funnel plot for effectiveness rates, (D) funnel plot for adverse effect rates.


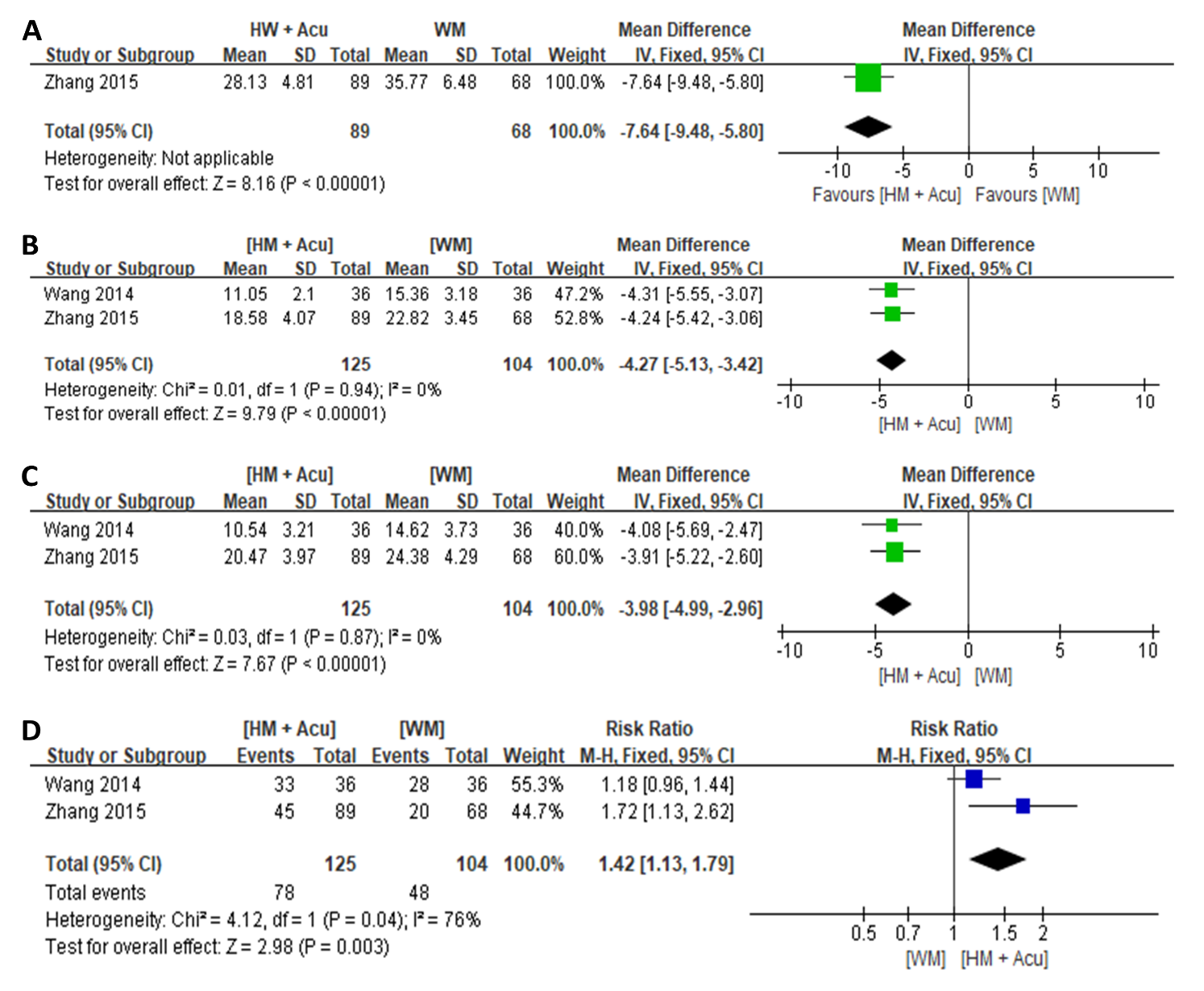


**Supplementary Figure 23.** Forest plot showing the effect of herbal medicine and acupuncture combined treatment versus Western medicine alone. (A) Forest plot for somatization, (B) forest plot for depression, (C) forest plot for anxiety, (D) forest plot for effectiveness rates.


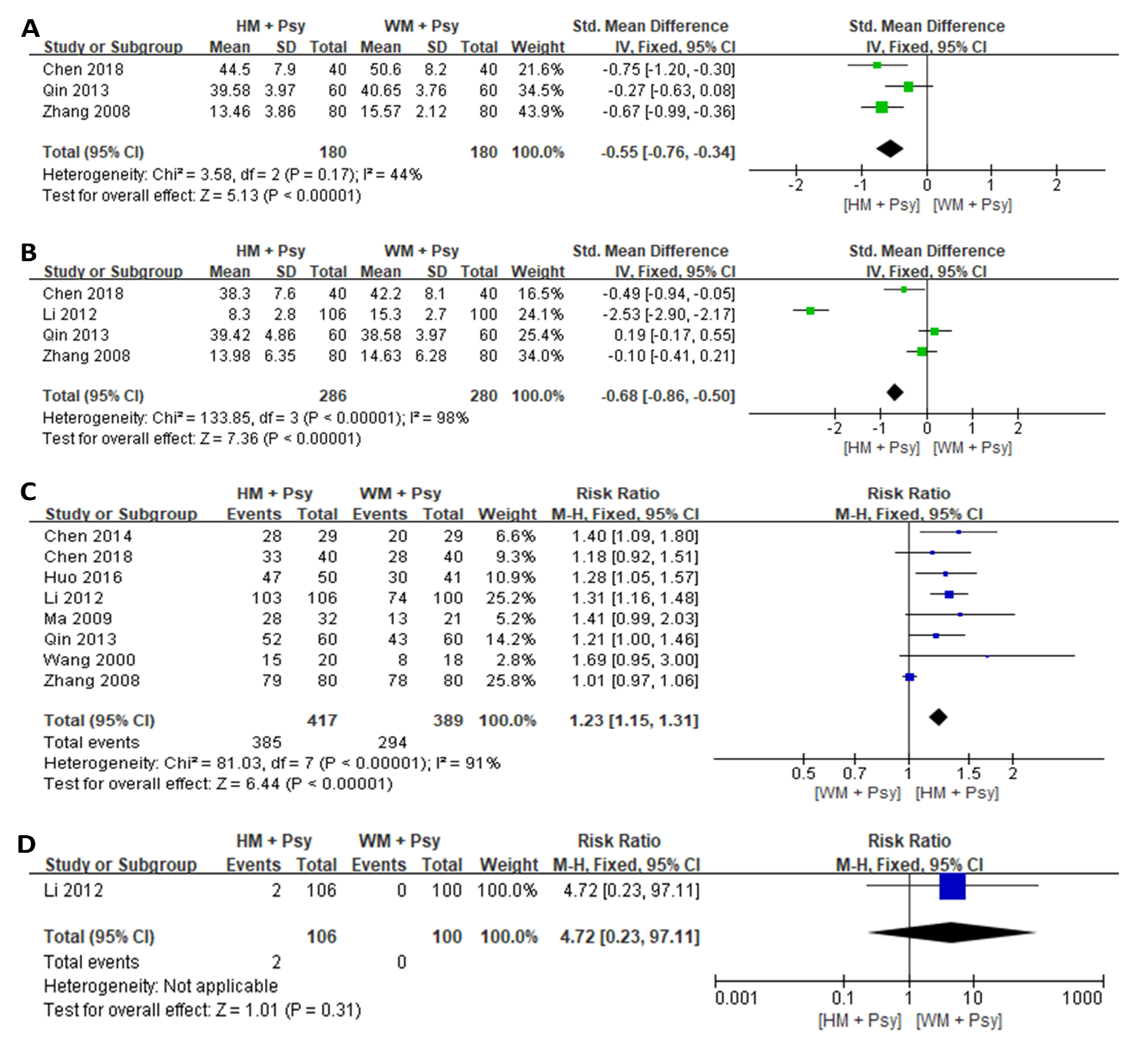


**Supplementary Figure 24.** Forest plot showing the effect of herbal medicine and psychology combined treatment versus Western medicine and psychology combined treatment. (A) Forest plot for depression, (B) forest plot for anxiety, (C) forest plot for effectiveness rates, (D) forest plot for adverse effect rates.


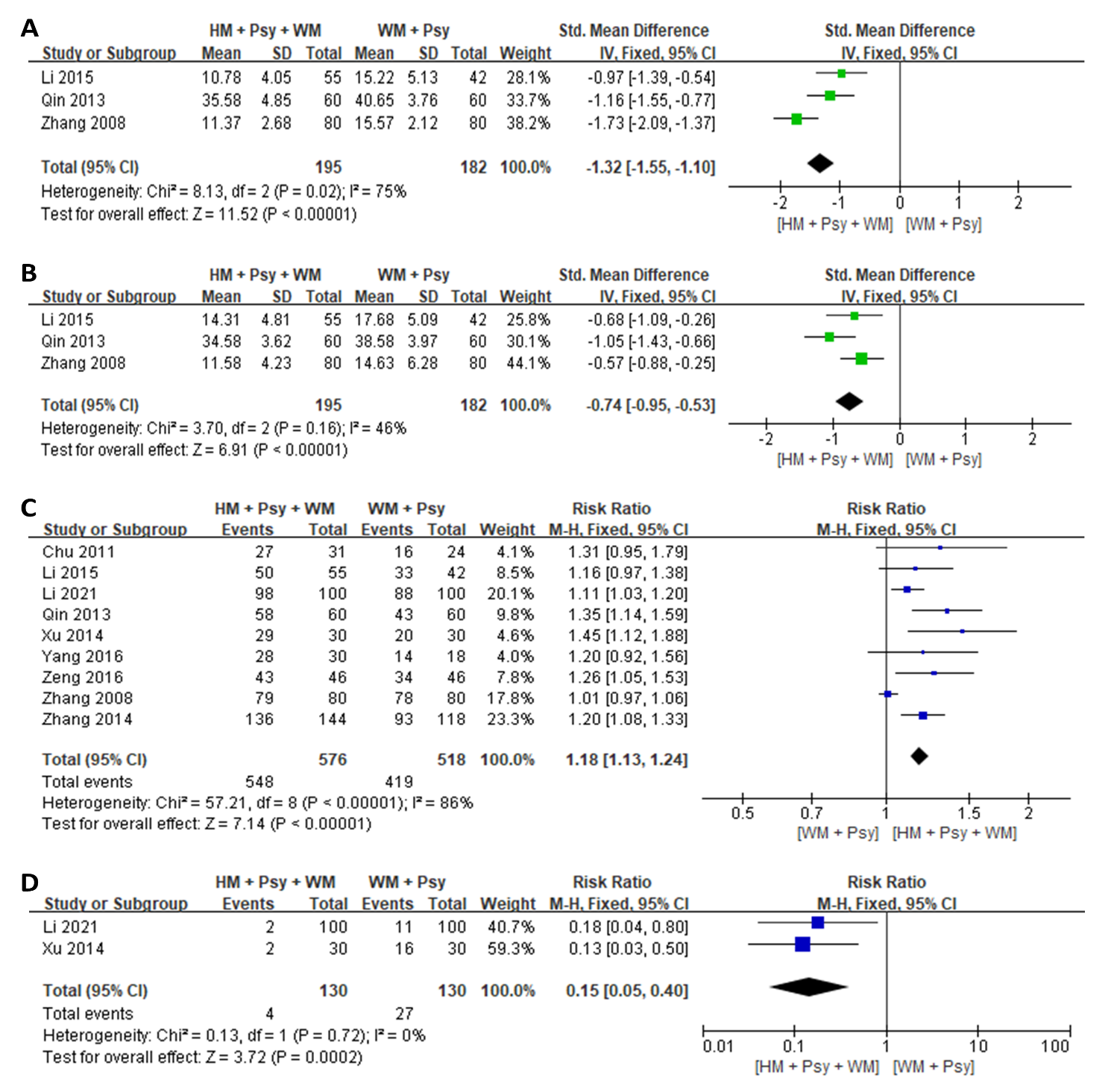


**Supplementary Figure 25.** Forest plot showing the effect of herbal medicine, psychology and Western medicine combined treatment versus Western medicine and psychology combined treatment. (A) Forest plot for depression, (B) forest plot for anxiety, (C) forest plot for effectiveness rates, (D) forest plot for adverse effect rates.


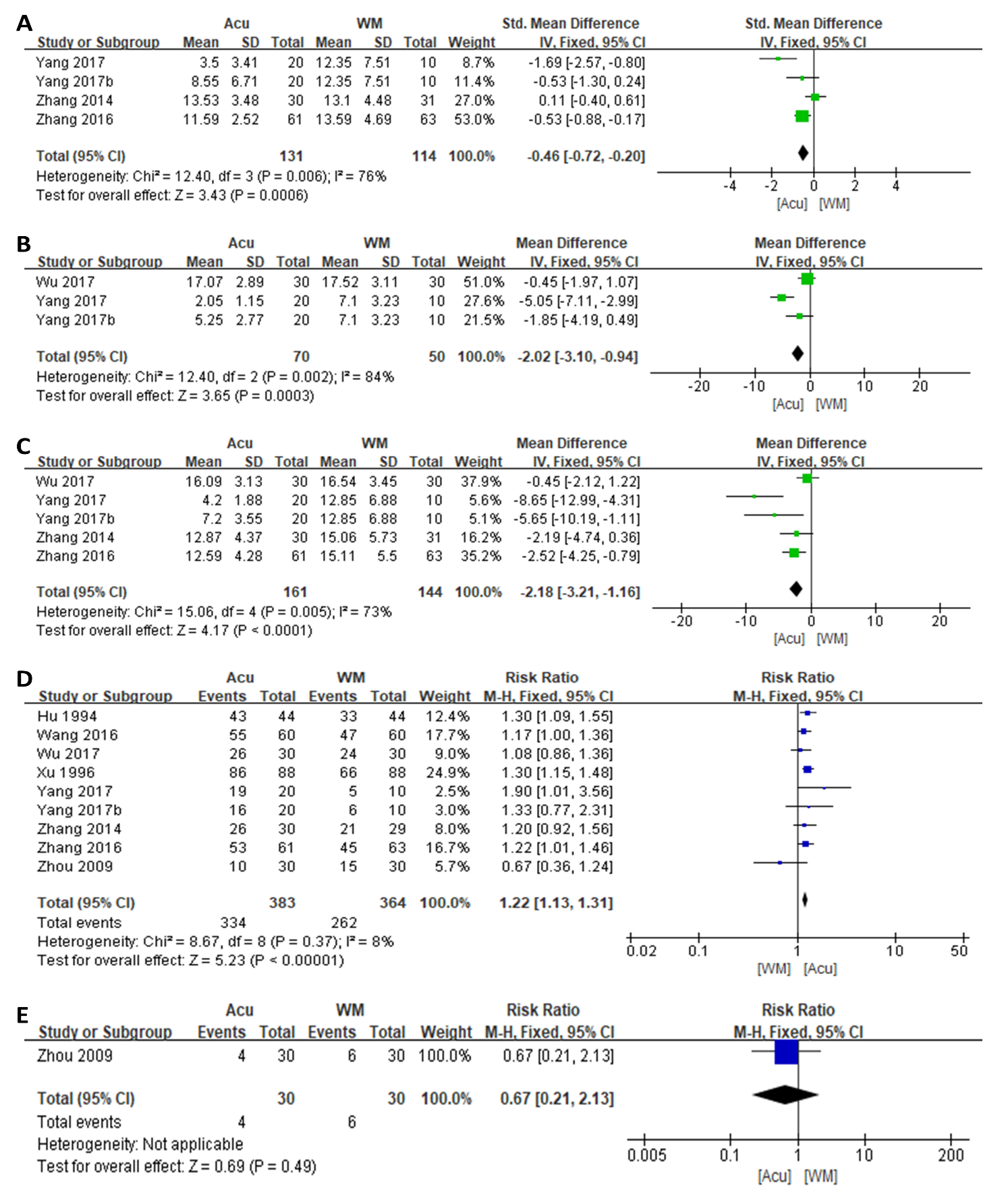


**Supplementary Figure 26.** Forest plot showing the effect of acupuncture versus Western medicine. (A) Forest plot for somatization, (B) forest plot for depression, (C) forest plot for anxiety, (D) forest plot for effectiveness rates, (E) forest plot for adverse effect rates.


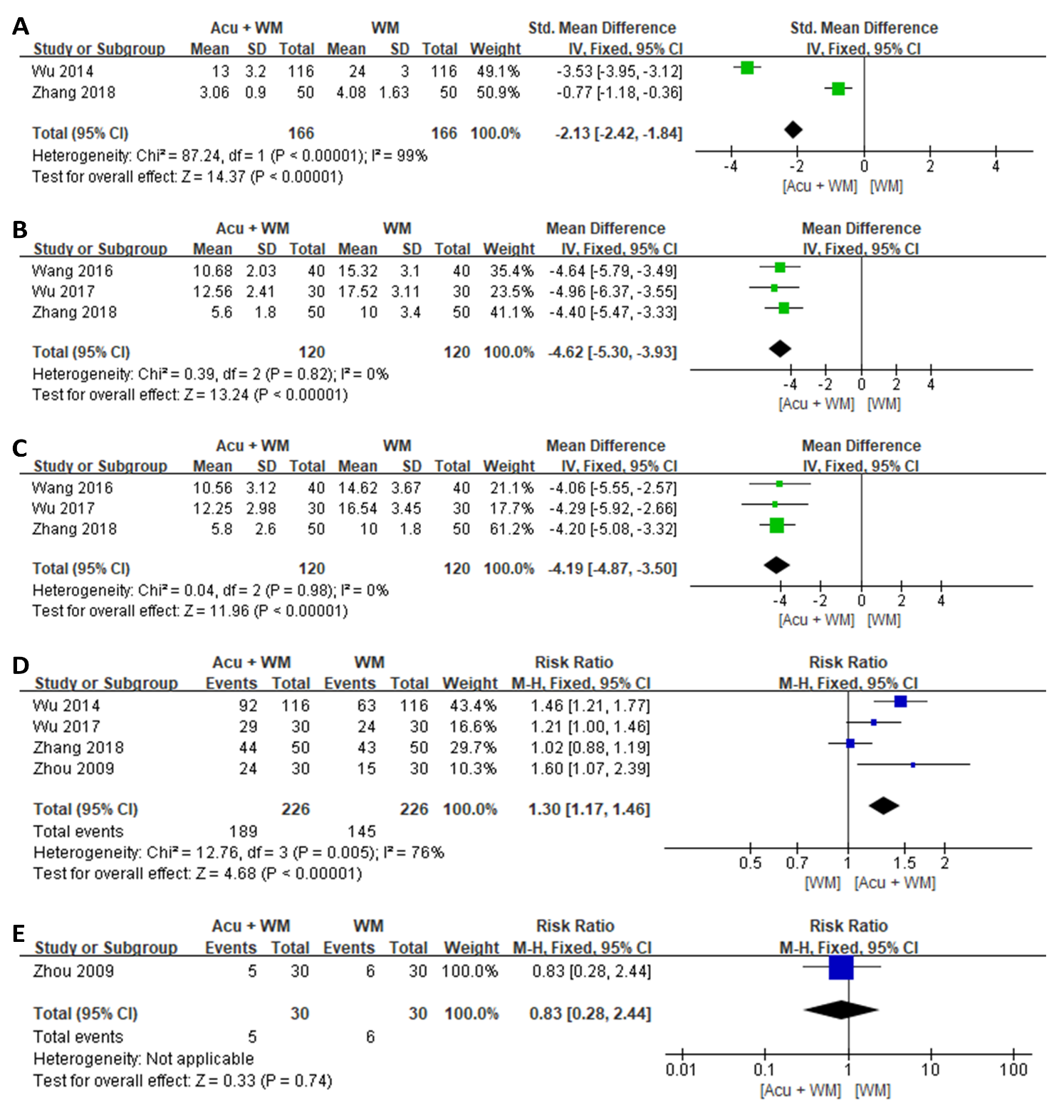


**Supplementary Figure 27.** Forest plot showing the effect of acupuncture and Western medicine combined treatment versus Western medicine alone. (A) Forest plot for somatization, (B) forest plot for depression, (C) forest plot for anxiety, (D) forest plot for effectiveness rate, (E) rorest plot for adverse effect rates.
